# How semantics works in Chinese relative clause processing: insights from eye tracking

**DOI:** 10.3389/fpsyg.2024.1294132

**Published:** 2024-02-19

**Authors:** Yan Liu, Chuanbin Ni

**Affiliations:** ^1^School of Foreign Languages and Cultures, Nanjing University of Posts and Telecommunications, Nanjing, China; ^2^School of Foreign Languages and Cultures, Nanjing Normal University, Nanjing, China

**Keywords:** language processing, semantics, Chinese relative clauses, eye tracking, concurrent processing model

## Abstract

Recent years have witnessed much research on semantic analysis and syntactic anatomy in ordinary language processing. However, it is still a matter of considerable debate about when and how the semantic integration of single word meanings works and interacts with syntax during on-line comprehension. This study, in an eye-tracking paradigm, took 38 native speakers of Mandarin Chinese as the participants and took Chinese relative clauses as stimuli to figure out the functions of semantics by investigating the conditioning semantic factors influencing and governing the word order variation of Chinese relative clauses during different processing stages. Accordingly, this study manipulated two syntactic variables, i.e., relative clause type and the position of the numeral-classifier sequence (NCL) in the relative clause, as well as a semantic variable, i.e., the abstractness of the head noun that the relative clause modified. Specifically, the study addressed two questions: (1) when semantics is activated and interacts with syntax and (2) how semantics affects syntax during the time course of Chinese relative clause processing. The results indicated that: (1) Semantics was activated and interacted with syntax during the early and late processing stages of Chinese relative clauses, which challenged the sequential order of syntactic and semantic processes, and supported the claims of the Concurrent Processing Model. (2) The syntactic order of the Chinese relative clause was affected by the semantic information of the head noun that the clause modified. Object-extraction relative clauses (ORCs) had a conjunction preference for the order “an object relative clause preceding the numeral-classifier sequence and the head noun.” Instead, the subject-extraction relative clause (SRC) which modified a concrete noun (CN) had a co-occurrence preference for the order “numeral-classifier sequence preceding the subject relative clause and the head noun,” while the subject-extraction relative clause which modified an abstract noun (AN) had a co-occurrence preference for the order “subject relative clause preceding the numeral-classifier sequence and the head noun.” The findings of this study were evaluated in light of the perspectives of truth value semantics of the syntactic components, the semantic compatibility of numeral-classifier sequence and its modified noun as well as the discourse functions of outer modifier nominals and inner modifier nominals.

## 1 Introduction

Understanding sentences and utterances is an effortless task in daily life, but it is still controversial concerning how the human language comprehension system processes a wide range of linguistic information within milliseconds. To solve it, a great deal of work on syntactic anatomy and semantic analysis in language comprehension has been done in the past. However, it remains a matter of considerable debate about when and how the semantic integration of single word meanings works and interacts with syntax during on-line comprehension. The purpose of this research is to investigate when semantics is activated and interacts with syntax during the time course of language comprehension, and how semantics affects syntax during the on-line processing of Chinese sentences.

### 1.1 Interactions of syntax and semantics

Two primary groups of psycholinguistic models arise from the divergence of competing processing theories: modular syntax-first models (van Gompel et al., 2005) and interactive models (Green and Mitchell, 2006). The modular syntax-first model argues that there is a language universal primacy of syntactic category processing over semantic processing (Friederici, 2011). Semantic integration can be influenced by syntactic analysis, but it does not contribute to the computation of syntactic structure. Initial syntactic analysis is autonomous; it appears prior to and independent of semantic processes, and dominates the process of sentence comprehension. Instead, semantic integration is dependent on the syntactic structure which is built by the modular parser. According to this model, language comprehension can be broadly conceived as consisting of three phases. During the first phase, local syntactic structures based on word category information are built independent of lexical-semantic information, but not vice versa. During the second stage, thematic role assignments proceed. If the initial syntax and the semantics do not match, reanalysis will take place in the third stage (Friederici, 2002). This model is largely influenced by traditional generative grammar which claims grammar is organized in the mind of the speaker as a number of hermetically sealed modules. Syntax, as an encapsulated brain system that operates automatically, can be separated from semantics (Chomsky, 1964).

Modular syntax-first models have been verified in a wide range of syntactic structures across languages (Gunter et al., 2000; Friederici, 2002, 2011; Hahne and Friederici, 2002). It was shown that among different types of information, syntactic information was processed at the earliest stage and semantic processing cannot proceed in the presence of syntactic violation. Instead, syntactic violations at this stage were shown to block semantic processing downstream (Friederici et al., 2004). Duncan et al. (1997) detected a strong attention-withdrawing effect in their experiment and concluded that syntax processing is autonomous from systems that use the same stimulus modality. In Hahne and Friederici’s (2002) research on German sentence comprehension, syntactic processing normally influenced semantic processes, but initial syntactic structure building occurred independent of semantic information. Pulvermüller et al. (2007) found that the syntactic process appeared in the early stage of sentence processing, was highly independent, and was not interfered with by other information. Bemis and Pylkkänen (2011) investigated the neural circuits underlying simple linguistic compositions and found the temporal ordering of the effects is consistent with many processing models that posit syntactic composition before semantic composition during the construction of linguistic, representations. However, the sequential syntax-first model has recently been challenged by plenty of evidence from eye-tracking and ERP studies.

As an alternative view, the interactive model, together with the constraint-based lexicalist model (MacDonald et al., 1994), the concurrent model (Boland, 1997), the non-syntactocentric dynamic model (Kuperberg, 2007), rejects the assumption of syntactic primacy. It divides language comprehension into three phases. During the first phase, the initial syntactic structure is constructed based on word category. During the second phase, lexical-semantic and morphosyntactic processing takes place together for the goal of thematic role assignment. During the third phase, varieties of information integrate with each other. Overall, semantic information can guide and contribute to the syntactic processing in language comprehension, which is manifested by such questions as how much information of word orders can be derived from semantics.

The interactive model is supported by a variety of evidence. Green and Mitchell (2006 found the reading of syntactically ambiguous sentences is affected by lexical semantics as well as global semantic information. Event-related potential studies have also suggested semantic processing can operate independently of morphosyntactic control (Kuperberg, 2007). Ye et al. (2006) concluded semantic analysis depended largely on local syntactic building in German, while syntactic and semantic processing proceeded independently and parallelly in the early stage of Chinese analysis. Syntactic analysis does not necessarily occur earlier than semantic operation in Chinese comprehension (Wang et al., 2013), and the time course of the interaction between syntax and semantics in Chinese sentence processing is also earlier compared with Indo-European languages (Ye et al., 2006). Besides, learners’ brain responses were inherently sensitive to the predictability of the incoming Chinese linguistic stimuli (Zhang et al., 2019). Take Chinese typical structures Subject–*Ba*–Object–VP structure (*Ba* structure) as examples, sentence meaning established prior to the onset of the processing reflected on N400 (Ye et al., 2006). However, the processing of the Chinese Object–*Bei*–Subject–VP structure (*Bei* structure) supported the claims of the syntax-first model (Zeng et al., 2020). The research on Chinese object-subject-verb construction suggested semantic integration proceeded even when the processing of syntactic category or syntactic subcategorization frame failed, and in this sense, syntactic processing was by no means an indispensable prerequisite for the activation of semantic integration in Mandarin Chinese (Zhang et al., 2013). The processing mechanism of the Chinese *Qing* structure was also consistent with the argument of the interactive model (Yang et al., 2021). However, the interactive model overemphasizes the role of the syntactic process and maintains the initial phase is a pure syntactic processing stage unaffected by any semantic information in sentence comprehension.

### 1.2 Syntax and semantics of Chinese relative clauses

Relative clauses (RCs) are crucial for understanding sentence structure analysis. Classifiers normally co-occur with demonstratives and/or quantifiers in Chinese discourse to modify and categorize noun phrases, forming numeral-classifier sequences (NCLs) and thus “NCL + head NP” construction in the study, such as “一个小偷” (*yi-ge xiaotou*, “one thief”). In Chinese, a relative clause, no matter whether a subject-extraction relative clause (SRC) or an object-extraction relative clause (ORC), can either be put preceding the head noun, or in other cases, separated from its head by a numeral-classifier sequence, resulting in two types of indefinite nominal constructions: a nominal with a pre-NCL modifier as outer modifier nominal (OMN), and a nominal with a post-NCL modifier as inner modifier nominal (IMN) (Zhang, 2006, p. 2), as shown in (1a) and (1b):

**(1) a. OMN (outer modifier nominal): RC + NCL + NP** (restrictive)

穿着黑衣的一个小偷

[*Ø chuanzhe heiyi de]*_RC_ [*yi-ge*]_NCL_ *xiaotou*

wear black clothes *DE* one-CL thief

“a thief who is wearing black clothes (not the one who is wearing blue clothes)”

**b. IMN (inner modifier nominal): NCL + RC + NP** (descriptive, nonrestrictive)

一个穿着黑衣的小偷

[*yi-ge*]_NCL_ [*Ø chuanzhe heiyi de]*_RC_ *xiaotou*

yi-CL wear black clothes *DE* thief

“a thief who is (incidentally) wearing black clothes”

Chinese RCs are subject to a peculiar ordering restriction not found with finite restrictive RCs. Chinese RCs expressing generic, individual-level properties must occur closer to the noun than those expressing episodic, temporally anchored, stage-level properties (Cinque, 2020). RC1 in (1a) is more appropriate for restrictive relatives than (1b) in that (1a) provides a better match between syntax and semantics for complex NPs with restrictive relatives. In (1a), the head noun and the relative clause are combined and serve together as the restrictor of the determiner, which can be a definite article or a quantifier.

Chinese RCs can be interpreted either non-restrictively or restrictively. This makes them similar to English post-numeral participial RCs, even if their non-restrictive interpretation should be kept distinct from that of English (or Romance) finite non-restrictives (Cinque, 2020). The pre-demonstrative position of RCs (and that of other modifiers) in Chinese appears to be a marked focus position. The markedness of this position seems confirmed by the counts reported in Hsu (2017, p. 75), where post-demonstrative RCs appear in corpora to be much more frequent than pre-demonstrative ones (Cinque, 2020).

**Figure 1:**
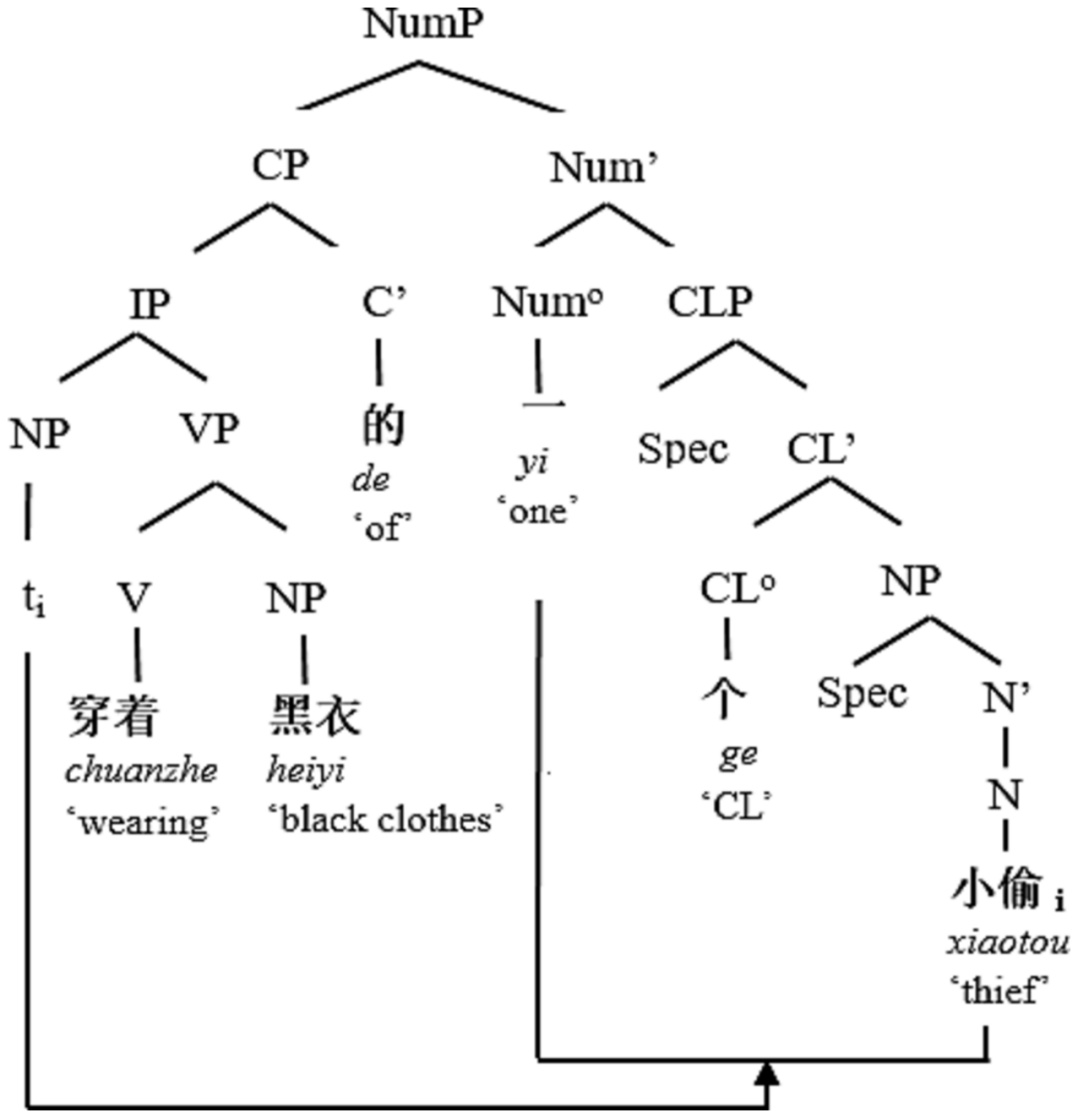
The syntax tree of “RC + NCL + CN” structure.

**Figure 2:**
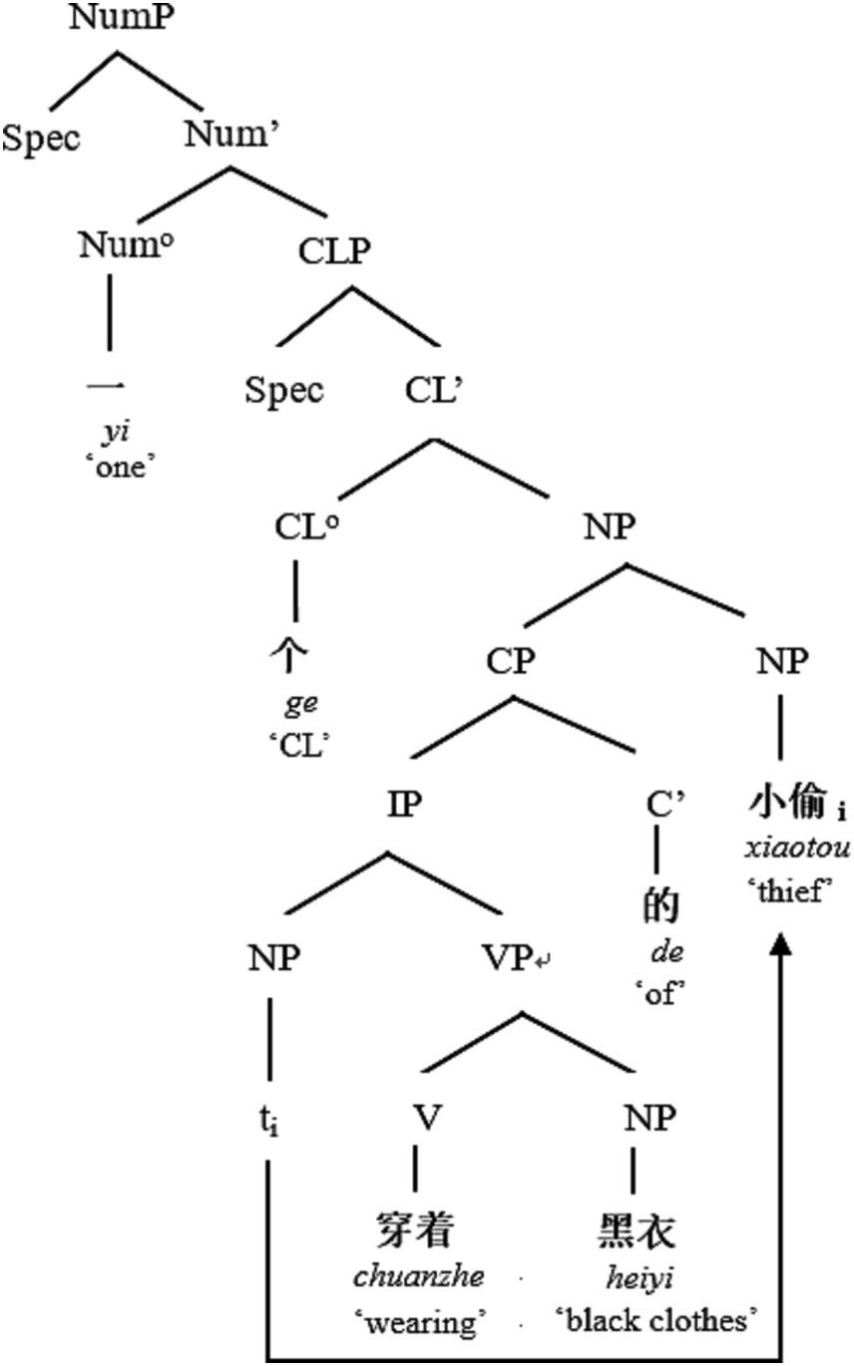
The syntax tree of “NCL + RC + NP” structure.

In this overall scenario, Chinese is puzzling that the post-demonstrative, post-numeral, post-classifier position of the RC is apparently open to a non-restrictive interpretation, which appears in conflict with the semantics of restrictives and non-restrictives (Lin, 2004). The finite/non-finite distinction is not overtly marked in Chinese, but they are both derived from the basic word order of the base structure “NP + VP + NP” (一个小偷穿着黑衣, *yi-ge xiaotou chuanzhe heiyi*, “A thief is wearing black clothes.”) by movement as shown below (see Figures 1, 2).

Over the last two decades, experts explored the interaction of syntax and semantics of Chinese relative clauses by analyzing two syntactic variables. The first one is the universality of the subject preference. Findings from Indo-European languages generally support a “universal” subject advantage: adults process SRCs more quickly and accurately than ORCs; children understand and produce SRCs earlier than ORCs. Research on typologically distinct languages and with groups other than monolingual native speakers, however, casts doubt on the universality of these patterns (Xu et al., 2019). Chinese relative clauses have word order properties that are distinctly rare across languages of the world; such properties provide a good testing ground to tease apart predictions regarding the relative complexity of subject and object RCs in acquisition and processing. While experts argued there may be no intrinsic processing asymmetry in Chinese RCs (Cheng et al., 2018), more studies in prenominal relative clauses of Mandarin Chinese supported the importance of structural locality and subject prominence for constructing gap-filler dependencies in prenominal relative clauses (Lin, 2018). Tsoi et al. (2019) found in the Chinese context, children showed a significant subject-over-object RC advantage. An error analysis suggested that children’s difficulty with object RCs reflected the tendency to interpret the sentential subject as the head noun. A subsequent corpus analysis suggested that children’s difficulty with object RCs may be in part due to distributional information favoring subject RC analyses. King and Marta (1995) reported a bilateral frontal slow negative potential for the entire RC region, and a phasic LAN effect immediately following the gap in English ORCs. They interpreted these results as evidence for ORCs incurring higher memory costs during processing. Much like other processing paradigms, ERP studies also report higher processing costs associated with ORCs in Chinese (Bulut et al., 2018).

The second variable is the preference for numeral-classifier sequence distribution in relative clauses, i.e., IMN (pre-RC classifiers) and OMN (post-RC classifiers) in terms of the distribution of numeral-classifier sequence. The two constructions differ in their semantic interpretation. Whereas the outer modifier nominal is exclusively specific, the inner modifier nominal can be either specific or non-specific. Sheng and Wu (2013) found that pre-RC classifiers tend to occur in subject-extraction relative clauses, while post-RC classifiers in object-extraction relative clauses. Moreover, the animacy of the head noun partially affects the distribution of classifiers, which tend to occur together with relatives embedded in the main clause. With the adoption of a word-based sentence production paradigm, Wu and Sheng (2014) revealed the production rate of demonstrative classifiers with object-extraction relative clauses was higher than that with subject-extraction relative clauses from the data. Demonstrative classifiers tended to occur in pre-RC positions, regardless of relative clause extraction types, and when demonstrative classifiers occurred in the post-RC position, object-extraction relative clauses had the production advantage.

This difference has been attributed variously to working memory limitations, syntactic factors, and perspective-shifting. Some researchers found that subject-extraction relative clauses were easier to process (e.g., Lin and Bever, 2006), whereas others supported the opposite (Hsiao and Gibson, 2003). In contrast to research on head-initial languages which has shown that English subject-extraction relative clauses are easier to process overall than object-extraction relative clauses, the findings for Mandarin are more mixed. The mechanism of the differences concerning the interaction of semantic integration and syntactic analysis is still unsettled, either.

### 1.3 Research gaps and research questions

The body of research demonstrates that there is ongoing debate about whether syntactic processing predominates and whether semantics functions in the initial processing stage across languages. The precise nature of the interaction between semantic integration and syntactic analysis is also difficult to draw from previous studies. What’s more, existing processing models are mostly constructed based on the analysis and experiments for Indo-European languages (Mitchell, 1994). Chinese language contrasts sharply with Indo-European languages in its morphosyntactic properties, which poses a challenge to Indo-European languages based psycholinguistic models. Plus, the laboratory results may be impacted by procedures used in previous studies (such as the self-paced reading and “violation” paradigm), which compelled participants to read language stimuli in an unnatural way, segment by segment (Osterhout et al., 1997).

This study, to get over the methodological flaws, applied the ecologically valid eye-tracking task to explore the processing mechanisms of the Chinese language in order to ensure participants read the linguistic items “in the wild.” The eye-tracking task assumes that the cognitive processing resources will be consumed when parsing either partial (i.e., targets in a sentence) or the whole sentence. First fixation duration and gaze duration are two parameters representing initial information processing difficulty (stage of lexical access) within a certain interest area, while regression path duration is a parameter for information processing in late stages (Yan et al., 2013). The study manipulated two syntactic variables and one semantic variable in stimulus sentences. We can investigate how semantics functions in the early processing stage by comparing the first fixation durations and gaze durations between conditions with different syntactic and semantic variables, while we explore the case in the late processing stage by comparing the regression path durations between conditions to see whether syntactic or semantic information was activated and how they interacted with each other in different processing stages, thus sketching a full picture of syntax-semantics interaction continuum in Chinese language comprehension.

Specifically, this study took Chinese native speakers as participants, and took Chinese relative clauses as stimuli to explore the function of semantics in Chinese on-line processing and evaluate the mechanism of semantic integration with syntax by means of dealing with two research questions. First, when will semantics be activated and interact with syntax during the time course of language comprehension? Second, how will semantics affect syntax during the on-line processing of Chinese sentences? We assume that even in the early stages of Chinese relative clauses comprehension, semantics will be activated and interact with syntax, continuing through the late processing stages, which is different from the case of Indo-European languages. Plus, Chinese sentence comprehension may rely on semantic cues to a greater degree than Indo-European languages do. Semantic information of the clause’s constituent words may have an impact on the syntactic order of Chinese relative clauses, but the time course of their interaction remains unsettled.

## 2 Methods

### 2.1 Participants

The eye-tracking experiment involved 38 students (23 female, 15 male, between 19 and 25 years old, *M* = 22.97 years, SD = 4.25), both undergraduates and graduates, from three universities in Jiangsu province of China. All participants were native speakers of Mandarin Chinese, right-handed, and had a normal or corrected-to-normal vision, had no known developmental, neurological, or psychiatric disorders. They gave written informed consent and received monetary compensation for their participation in the study.

### 2.2 Stimuli

Prior research showed inner modifier nominal constructions and outer modifier nominal constructions denote different meanings (Zhang, 2006). Additionally, prior research revealed a strong relationship between word order and the abstractness of head nouns, and people find it more difficult to understand English sentences with object-extraction relative clauses than those with subject-extraction relative clauses (e.g., Wanner and Maratsos, 1978), while the case may be different in Chinese language.

In order to explore the conditioning semantic factors that influence the syntactic variation of these two constructions in comprehension, this study manipulated two syntactic variables, i.e., subject-extraction relative clauses and object-extraction relative clauses, the position of a numeral-classifier sequence in relative clauses, i.e., inner modifier nominal and outer modifier nominal, as well as a semantic variable, abstractness of the head noun that the relative clause modifies, i.e., abstract noun (AN) and concrete noun (CN). Accordingly, a 2 × 2 × 2 ANOVAs for Repeated Measures design was adopted and thus yielded eight conditions (from condition “a” to “h”), i.e., “NCL + SRC + CN,” “NCL + ORC + CN,” “NCL + SRC + AN,” “NCL + ORC + AN,” “SRC + NCL + CN,” “ORC + NCL + CN,” “SRC + NCL + AN” and “ORC + NCL + AN.”

Materials for the eight groups of stimuli were selected following the three steps. First, 100 nouns (including 50 concrete nouns and 50 abstract nouns) were selected from the high-frequency category of the Center Chinese Linguistics corpus (logW>1, Cai and Brysbaert, 2010). The abstractness of the 100 nouns was then assessed by 48 participants who took part in experiments of material preparation on a 7-point Likert scale (“1” represented concreteness of nouns and “7” indicated abstractness of nouns, *M*_cn_ ≥ 6.854 ± 0.649, *M*_an_ ≤ 1.974 ± 1.821, *p* < 0.01). For example, the concept of “小偷” (*xiaotou*, “thief”), “老虎” (*laohu*, “tiger”), “风筝” (*fengzheng*, “kite”), “美貌” (*meimao*, “beauty”), “恐惧” (*kongju*, “fear”) and “精神” (*jingshen*, “spirit”) ranged along the continuum from concreteness to abstractness, extending from human through animate to inanimate. Thirty two most concrete nouns and 32 most abstract nouns were singled out this way, which were well balanced across conditions in terms of stroke numbers (*M*_cn_ = 10.823 ± 3.839, *M*_an_ = 11.141 ± 3.251, *p* = 0.522).

Second, a sentence completion survey followed by. Each of the 48 participants for material preparation was presented with four selected critical nouns, and was required to make a pair of two meaningful and grammatical sentences with a given syntactic structure for each noun, as exemplified in 2(a-b). Critical words were positioned inside to avoid sentence-final wrap-up effects.

Third, a separate group of 22 participants for stimuli selection was instructed to make judgments on the frequency, fluency, and naturalness of the newly devised pairs of sentences on a 7-point Likert scale (“7” represented the most frequent, fluent, and natural). A total of 128 pairs of two sentences with the highest scores were selected on the grounds of their judgment as exemplified in (2) Condition (a-d):

**(2) Condition a: NCL + SRC + CN**

记者看到一个穿着黑衣的小偷已逃离了现场。

*jizhe kandao yi-ge [*Ø *chuanzhe heiyi de] xiaotou yi taoli-le xianchang.*

journalist see one-CL wear black clothes *DE* thief already flee-PERF scene

“The journalist sees a thief in black has already fled the scene.”

**Condition b: NCL + ORC + CN**

记者看到一个子弹击中的小偷已逃离了现场。

*jizhe kandao yi-ge [zidan jizhong* Ø *de] xiaotou yi taoli-le xianchang.*

journalist see one-CL bullet hit *DE* thief already flee-PERF scene

“The journalist sees a thief that the bullet hit has already fled the scene.”

**Condition c: NCL + SRC + AN**

记者看到一片献给孩子的爱心已送到了学校。

*jizhe kandao yi-pian [*Ø *xiangei haizi de] aixin yi songdao-le xuexiao*

journalist see one-CL piece dedicate to children *DE* love already send-PERF to school

“The journalist sees the love which is dedicated to children has already been sent to the school.”

**Condition d: NCL + ORC + AN**

记者看到一片社会奉献的爱心已送到了学校。

*jizhe kandao yi-pian [shehui fengxian* Ø *de] aixin yi songdao-le xuexiao*

journalist see one-CL piece society dedicate *DE* love already send-PERF to school

“The journalist sees the love which the society dedicated has already been sent to the school”

The other pair of quadruplet sentences was created by reversing the position of the numeral-classifier sequence and the relative clause in sentences of Condition a-d, producing sentences of Condition e-h:

**Condition e: SRC + NCL + CN**

记者看到穿着黑衣的一个小偷已逃离了现场。

*jizhe kandao [*Ø *chuanzhe heiyi de] yi-ge xiaotou yi taoli-le xianchang*.

journalist see wear black clothes *DE* one-CL thief already flee-PERF scene

“The journalist sees a thief in black has already fled the scene.”

**Condition f: ORC + NCL + CN**

记者看到子弹击中的一个小偷已逃离了现场。

*jizhe kandao [zidan jizhong* Ø *de] yi-ge xiaotou yi taoli-le xianchang.*

journalist see bullet hit *DE* one-CL thief already flee-PERF scene

“The journalist sees a thief that the bullet hit has already fled the scene.”

**Condition g: SRC + NCL + AN**

记者看到献给孩子的一片爱心已送到了学校。

*jizhe kandao [*Ø *xiangei haizi de] yi-pian aixin yi songdao-le xuexiao.*

journalist see dedicate to children *DE* one-CL piece love send-PERF to school

“The journalist sees the love which is dedicated to children has already been sent to the school.”

**Condition h: ORC + NCL + AN**

记者看到社会奉献的一片爱心已送到了学校。

*jizhe kandao [shehui fengxian* Ø *de] yi-pian aixin yi songdao-le xuexiao.*

journalist see society dedicate *DE* one-CL piece love already send-PERF to school

“The journalist sees the love which the society dedicated has already been sent to the school.”

In this way, 256 target sentences, pertaining to the selected 64 nouns, were constructed as critical items (as illustrated in Table 1). These sentences were Latin-squared into four lists. Each list included 64 sentences, covering all eight conditions and only one sentence from each set. Each list was also incorporated with 64 filler sentences, each of which was embedded with a numeral-classifier sequence, but without any relative clauses, such as: “他在好朋友家里看到三个小巧玲珑的铅笔盒。” (*ta zai haopengyou jia li kandao san-ge xiaoqiaolinglong de qianbihe*, “He saw three small and delicate pencil cases at his best friend’s house”). The 128 trial items were divided into 4 blocks in the experiment.

**Table 1:** Research design of the experiment.

Conditions	Illustrations	Examples
a	NCL + SRC + CN	记者看到一个穿着黑衣的小偷已逃离了现场。 journalist see one-CL wear black clothes *DE* thief already flee-PERF scene “The journalist sees a thief in black has already fled the scene.”
b	NCL + ORC+CN	记者看到一个子弹击中的小偷已逃离了现场。 journalist see one-CL bullet hit *DE* thief already flee-PERF scene “The journalist sees a thief that the bullet hit has already fled the scene.”
c	NCL + SRC + AN	记者看到一片献给孩子的爱心已送到了学校。 journalist see one-CL piece dedicate to children *DE* love already send-PERF to school “The journalist sees the love which is dedicated to children has already been sent to the school.”
d	NCL + ORC+AN	记者看到一片社会奉献的爱心已送到了学校。 journalist see one-CL piece society dedicate *DE* love already send-PERF to school “The journalist sees the love which the society dedicated has already been sent to the school.”
e	SRC + NCL + CN	记者看到穿着黑衣的一个小偷已逃离了现场。 journalist see wear black clothes *DE* one-CL thief already flee-PERF scene “The journalist sees a thief in black has already fled the scene.”
f	ORC+NCL + CN	记者看到子弹击中的一个小偷已逃离了现场。 journalist see bullet hit *DE* one-CL thief already flee-PERF scene “The journalist sees a thief that the bullet hit has already fled the scene.”
g	SRC + NCL+ AN	记者看到献给孩子的一片爱心已送到了学校。 journalist see dedicate to children *DE* one-CL piece love send-PERF to school “The journalist sees the love which is dedicated to children has already been sent to the school.”
h	ORC+NCL+ AN	记者看到社会奉献的一片爱心已送到了学校。 journalist see society dedicate *DE* one-CL piece love already send-PERF to school “The journalist sees the love which the society dedicated has already been sent to the school.”

### 2.3 Procedure

First, 38 participants involved in the eye-tracking experiment were required to sign a pencil-and-paper consent form, and their background information was surveyed.

Then, they were seated in a comfortable chair approximately 65 cm off a computer screen with their chin resting on a pad to minimize head movements. Data were recorded by Eyelink 2000 eye tracker. Viewing was monocular tracking of the right pupil, and cornea was performed at the sampling rate of 1,000 Hz. Participants were instructed to do nine-point calibration and validation. The maximum and average errors were below 1° of visual angle. All stimuli were presented in Chinese Song typeface and in 28-point font with a gray background color, and displayed on the center of the screen from left to right.

Each participant was randomly assigned one list of stimuli and was instructed to make a dichotomic choice to judge a sentence to be acceptable or unacceptable grammatically. If acceptable, they should press the “F” key. If not, the “J” key. Some stimuli and filler sentences were followed by “yes”-or-“no” questions for comprehension (with a ratio of 1: 3.5), to ensure that the participants were attentive and alert during the whole process of the experiment. Participants should respond to them correctly. After that, participants were assigned a separate group of 24 practice trials prior to the experimental blocks.

The experiment lasted about 25 min, and participants took short breaks between blocks. After the break, drift correction was conducted so that the eye-tracking recording did not drift away.

### 2.4 Data analysis

**Figure 3:**
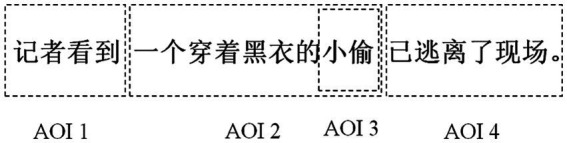
Illustrations of interest area templates.

As exemplified in Figure 3, the target sentence was divided into four parts and drawn as AOI 1 (Area of Interest 1), AOI 2, AOI 3, and AOI 4, respectively. AOI 2 and AOI 3 served as the critical parts, based on which the data were exported and analyzed as in previous relevant studies (Rayner, 2009).

Data from three participants (2 males and 1 female) were excluded from the analyses due to too much track loss of the critical regions (track loss accounted for 11.437%). The remaining 35 participants served as the basis of all the data. Average accuracy was 92.265% across eight critical conditions, suggesting participants read the sentences attentively. All incorrect responses, together with responses faster than 300 ms, were removed (82 observations in all). Furthermore, outliers above or below 2.5 *SD*s of the participants’ means for each index at each region were excluded from the analysis (Felser and Cunnings, 2012; Li et al., 2016).

## 3 Results

The acceptability of eight conditions could test whether the semantic function in comprehension. Comparison of eye-tracking data between different syntactic and semantic conditions could examine when semantic activation occurs and how semantics integrates with syntax in Chinese sentence processing stages. SPSS 21.0 was applied as the statistical analysis tool in the descriptive and inferential analysis of the data. The ANOVA for Repeated Measures and further pairwise comparison between conditions were also adopted to calculate.

### 3.1 Reaction time and acceptability

**Figure 4:**
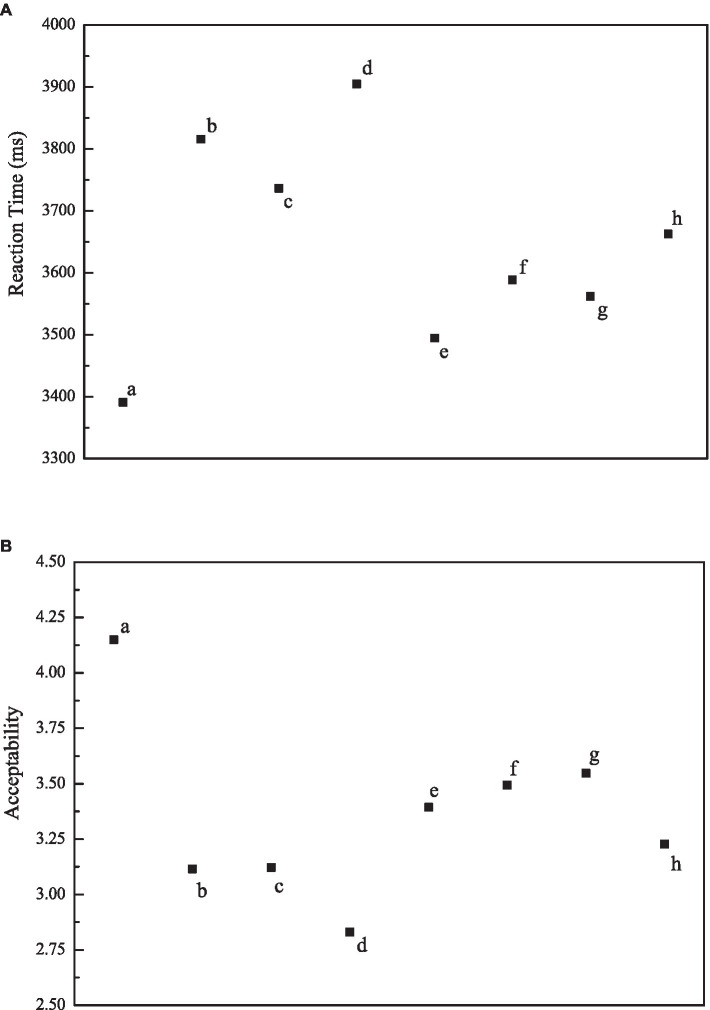
Illustration of average reaction time and acceptability by condition (a–h).

We numbered stimuli sentences with conditions “a” to “h” respectively for convenience. As shown in Figure 4, the reaction time to condition “a” was generally shorter than that to any other condition, while the reaction time to condition “d” was the longest, implying it took participants the longest time to evaluate the acceptability of sentences on condition “d.” There was a significant main effect on “abstractness of head nouns,” *F*_1_ (1, 34) = 6.121, *MSE* = 237335.751, *p* = 0.019, *η^2^_p_* = 0.153; *F*_2_*(*1, 31) = 4.823, *MSE* = 282494.425, *p* = 0.036, *η^2^_p_* = 0.135, demonstrating the abstractness of the head noun that the relative clause modified played a significant role in language processing, while no significant main effect on “relative-clause types” or “numeral-classifier sequence position” was detected, nor was the interactive effect between any two.

As for acceptability, items on condition “a” were the most acceptable, while those on condition “d” were the least acceptable. There was a significant main effect on “abstractness of head nouns,” *F*_1_(1, 34) = 8.169, *MSE* = 1.085, *p* = 0.007, *η^2^_p_* = 0.194; *F*_2_(1, 31) = 6.722, *MSE* = 1.187, *p* = 0.014, *η^2^_p_* = 0.178), as well as on “clause type,” *F*_1_(1, 34) = 13.077, *MSE* = 0.799, *p* = 0.001, *η^2^_p_* = 0.278; *F*_2_(1, 31) = 9.025, *MSE* = 1.256, *p* = 0.005, *η^2^_p_* = 0.225, demonstrating the abstractness of head nouns and relative-clause types both played significant roles in Chinese language comprehension. A significant interactive effect was also detected across conditions.

### 3.2 Eye-tracking data

#### 3.2.1 The time course of semantic activation

**Figure 5:**
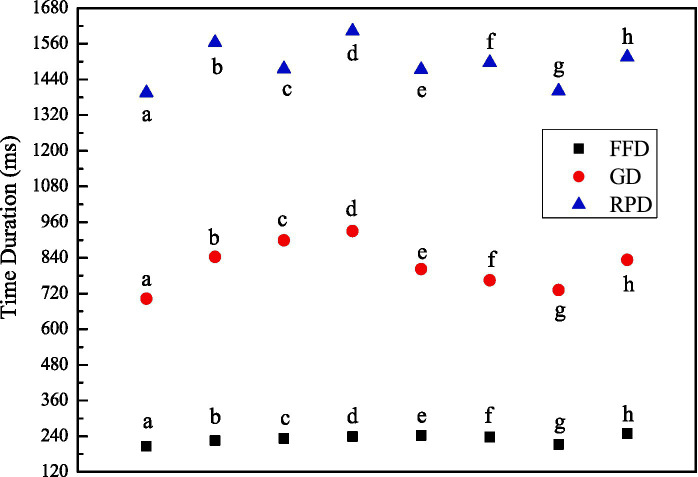
Illustration of eye-tracking results by condition (a–h).

We took three typical temporal dimension measures indicating the time course of eye movements as the indexes to investigate the language processing continuum in which semantics were activated. The indexes related to the early processing stage included first fixation duration (FFD) and gaze duration (GD), while the index related to the late stage involved regression path duration (RPD). Table 2 and Figure 5 showed participants’ eye-tracking results by condition.

**Table 2:** Summary of eye-tracking results by condition.

Condition	Early stage		Late stage
	FFD	GD	RPD
a	206.057 ± 41.410	701.943 ± 173.579	1395.114 ± 127.756
b	225.343 ± 50.667	842.971 ± 146.726	1563.914 ± 178.354
c	232.029 ± 41.273	898.400 ± 239.074	1475.771 ± 145.573
d	239.086 ± 41.067	929.829 ± 199.006	1602.171 ± 156.011
e	242.514 ± 46.968	801.743 ± 183.747	1473.143 ± 97.237
f	236.514 ± 46.084	764.457 ± 179.691	1496.914 ± 133.208
g	212.371 ± 35.874	731.143 ± 155.535	1400.543 ± 113.598
h	248.771 ± 66.422	832.629 ± 179.209	1515.143 ± 133.160

##### 3.2.1.1 The early processing stage

Participants’ mean first fixation duration on condition “a” was the shortest, while that on condition “f” was the longest. The first fixation duration yielded a significant main effect on SRC/ORC variable, *F*_1_(1, 34) =10.644, *MSE* = 1323.444, *p* = 0.003, *η^2^_p_* = 0.238, *F*_2_(1, 31) =6.836, *MSE* = 1330.745, *p* = 0.014, *η^2^_p_* = 0.181. No significant effect was observed on IMN/OMN variable, *F*_1_(1, 34) =3.154, *MSE* = 1967.103, *p* > 0.05, *η^2^_p_* = 0.085; *F*_2_(1, 31) =3.865, *MSE* = 1402.318, *p* = 0.058, *η^2^_p_* = 0.111 or CN/AN variable (*F*_1_ < 1; *F*_2_ < 1), while significant interactive effect was detected among the three variables.

Participants’ mean gaze duration on condition “a” was the shortest, while that on condition “d” was the longest. The gaze duration yielded a significant main effect on IMN/OMN variable, *F*_1_(1, 34) = 6.649, *MSE* = 38911.173, *p* = 0.014, *η^2^_p_* = 0.164; *F*_2_(1, 31) = 9.114, *MSE* = 22360.687, *p* = 0.005, *η^2^_p_* = 0.227, CN/AN variable, *F*_1_(1, 34) = 7.749, *MSE* = 44546.557, *p* = 0.009, *η^2^_p_* = 0.186; *F*_2_(1, 31) = 21.316, *MSE* = 12639.443, *p* < 0.01, *η^2^_p_* = 0.407 as well as SRC/ORC variable, *F*_1_(1, 34) = 10.184, *MSE* = 24059.558, *p* = 0.003, *η^2^_p_* = 0.230; *F*_2_(1, 31) = 5.963, *MSE* = 33164.957, *p* = 0.021, *η^2^_p_* = 0.161. A significant interactive effect was also detected among the three variables, demonstrating syntactic information and semantic information were both activated and interacted in the early processing stage of Chinese sentences.

##### 3.2.1.2 The late processing stage

Participants’ mean regression path duration on condition “a” was the shortest, while that on condition “d” was the longest. The regression path duration yielded a significant main effect on IMN/OMN variable by participants, *F*_1_(1, 34) = 8.807, *MSE* = 11361.023, *p* = 0.005, *η^2^_p_* = 0.206, on CN/AN variable by items, *F*_2_(1, 31) = 8.153, *MSE* = 6820.842, *p* = 0.008, *η^2^_p_* = 0.208 as well as on SRC/ORC variable, *F*_1_(1, 34) = 47.320, *MSE* = 17380.311, *p* < 0.01, *η^2^_p_* = 0.582; *F*_2_(1, 31) = 83.412, *MSE* = 8981.909, *p* < 0.01, *η^2^_p_* = 0.729. A significant interactive effect was detected between the IMN/OMN variable and the abstractness of the head noun, as well as between the IMN/OMN variable and relative-clause types, demonstrating that the syntactic and semantic information interacted, and the abstractness of head nouns influenced the distributional positions of numeral-classifier sequences in the late processing stage of Chinese sentences.

#### 3.2.2 The distribution preference of numeral-classifier sequences

More comparisons would be made to explore how semantics works by examining how the abstractness of head nouns influences the distributional position of numeral-classifier sequences in Chinese processing. When the abstractness of head nouns was taken as the focus, we took AOI 3 as the critical part, and four pairs of groups (summarized to be G1-G4) were yielded accordingly as shown in Table 3.

**Table 3:** Four groups with the abstractness of head nouns as the focus.

Group	Conditions	Examples
G1	a	记者看到一个穿着黑衣的**小偷**已逃离了现场。 journalist see one-CL wear black clothes *DE* thief already flee-PERF scene “The journalist sees a thief in black has already fled the scene.”
	e	记者看到穿着黑衣的一个**小偷**已逃离了现场。 journalist see wear black clothes *DE* one-CL thief already flee-PERF scene “The journalist sees a thief in black has already fled the scene.”
G2	c	记者看到一片献给孩子的**爱心**已送到了学校。 journalist see one-CL piece dedicate to children *DE* love already send-PERF to school “The journalist sees the love which is dedicated to children has already been sent to the school.”
	g	记者看到献给孩子的一片**爱心**已送到了学校。 journalist see dedicate to children *DE* one-CL piece love send-PERF to school “The journalist sees the love which is dedicated to children has already been sent to the school.”
G3	b	记者看到一个子弹击中的**小偷**已逃离了现场。 journalist see one-CL bullet hit *DE* thief already flee-PERF scene “The journalist sees a thief that the bullet hit has already fled the scene.”
	f	记者看到子弹击中的一个**小偷**已逃离了现场。 journalist see bullet hit *DE* one-CL thief already flee-PERF scene “The journalist sees a thief that the bullet hit has already fled the scene.”
G4	d	记者看到一片社会奉献的**爱心**已送到了学校。 journalist see one-CL piece society dedicate *DE* love already send-PERF to school “The journalist sees the love which the society dedicated has already been sent to the school.”
	h	记者看到社会奉献的一片**爱心**已送到了学校。 journalist see society dedicate *DE* one-CL piece love already send-PERF to school “The journalist sees the love which the society dedicated has already been sent to the school.”

##### 3.2.2.1 Acceptability

In SRC-embedded sentences, significant differences were detected in participants’ mean acceptability between the two paired items in Group 1 (*MD* = 0.757, *p* = 0.005, *Cohen’s D* = 0.694) and Group 2 (*MD* = 0.425, *p* = 0.036, *Cohen’s D* = 0.511). Specifically, participants had significantly higher acceptability of condition “a” than that of its paired condition “e,” but they had significantly lower acceptability of condition “c” than that of its paired condition “g,” indicating the distributional preference of numeral-classifier sequences was influenced by semantic information, i.e., the abstractness of head nouns that subject relative clauses modified, such as “小偷” (*xiaotou*, “thief”) and “爱心” (*aixin*, “love”).

In ORC-embedded sentences, participants had significantly higher acceptability of condition “f” than that of its paired condition “b,” and they had significantly higher acceptability of condition “h” than that of its paired condition “d” (*MD* = 0.395, *p* = 0.042, *Cohen’s D* = 0.496), indicating object-extraction relative clauses had a conjunction preference for a pre-NCL modifier as outer modifier nominal.

##### 3.2.2.2 First fixation duration

Significant differences were detected in participants’ mean FFD between the two paired items in Group 1 (*MD* = 36.457, *p* = 0.001, *Cohen’s D* = 0.823) and the two pairs in Group 2 (*MD* = 19.657, *p* = 0.037, *Cohen’s D* = 0.508). Specifically, the participants’ mean FFD on condition “a” was significantly shorter in Group 1, while their mean FFD on condition “g” was significantly shorter in Group 2, indicating the distributional position of numeral-classifier sequences was related to, or influenced by the abstractness of the head noun that the subject-extraction relative clause modified. No significant difference was observed in participants’ mean FFD between the two paired items of Group 3 (*p* = 0.338) or Group 4 (*p* = 0.466).

##### 3.2.2.3 Gaze duration

The participants’ mean GD had significant differences between the two paired items in Group 1 (*MD* = 99.800, *p* = 0.022, *Cohen’s D* = 0.558), Group 2 (*MD* = 167.257, *p* = 0.001, *Cohen’s D* = 0.829), Group 3 (*MD* = 78.514, *p* = 0.049, *Cohen’s D* = 0.479) and Group 4 (*MD* = 97.200, *p* = 0.035, *Cohen’s D* = 0.513). In Group 1, their mean GD on condition “a” was significantly shorter, while their mean GD on condition “g” was significantly shorter in Group 2. Similarly, participants’ mean GD on condition “f” was shorter than its counterpart condition in Group 3, while their mean GD on condition “h” was shorter than its counterpart condition in Group 4, all to a significant degree.

##### 3.2.2.4 Regression path duration

The participants’ mean RPD had significant differences between the two paired items in Group 1 (*MD* = 78.029, *p* = 0.005, *Cohen’s D* = 0.687), Group 2 (*MD* = 75.229, *p* = 0.019, *Cohen’s D* = 0.576) as well as Group 4 (*MD* = 87.029, *p* = 0.014, *Cohen’s D* = 0.600). Specifically, their mean RPD on condition “a” was significantly shorter in Group 1, while that on condition “g” was significantly shorter in Group 2. Similarly, their mean RPD on condition “f” was shorter than its counterpart condition in Group 3 (*MD* = 67.001, *p* = 0.079, *Cohen’s D* = 0.426), while that on condition “h” was significantly shorter than its counterpart in Group 4.

In view of the acceptability and eye-tracking results across conditions, object-extraction relative clauses and subject-extraction relative clauses had different preferences for numeral-classifier sequences’ distributional positions. Object-extraction relative clauses had a co-occurrence preference for outer modifier nominals, i.e., an object relative clause preceding a numeral-classifier sequences, forming the construction “ORC+NCL + AN/CN.” However, numeral-classifier sequences’ distributional position in subject relative clauses was affected by the semantic factor, i. e. the abstractness of the head noun which the subject-extraction relative clause modified. Specifically, the subject-extraction relative clause which modified a concrete head noun had a conjunction preference for inner modifier nominals, with the numeral-classifier sequence preceding the subject relative clause and head noun, forming construction “a,” as in “一个穿着黑衣的小偷” (*yi-ge chuanzhe heiyi de xiaotou*, “a thief in black”), while the subject-extraction relative clause which modified an abstract head noun had a co-occurrence preference for outer modifier nominals, forming construction “g,” as in “献给孩子的一片爱心” (*xiangei haizi de yi-pian aixin*, “the love which is dedicated to children”). The divergent preference was demonstrated in Table 4.

**Table 4:** Co-occurrence preference of relative clauses over OMN and IMN.

RC type	Head noun in RCs	Preference for OMN/IMN	Examples
ORC	Concrete head NPs	OMN	记者看到子弹击中的一个小偷已逃离了现场。 journalist see bullet hit *DE* one-CL thief already flee-PERF scene “The journalist sees a thief that the bullet hit has already fled the scene.”
	Abstract head NPs	OMN	记者看到社会奉献的一片爱心已送到了学校。 journalist see society dedicate *DE* one-CL piece love already send-PERF to school “The journalist sees the love which the society dedicated has already been sent to the school.”
SRC	Concrete head NPs	IMN	记者看到一个穿着黑衣的小偷已逃离了现场。 journalist see one-CL wear black clothes *DE* thief already flee-PERF scene “The journalist sees a thief in black has already fled the scene.”
	Abstract head NPs	OMN	记者看到献给孩子的一片爱心已送到了学校。 journalist see dedicate to children *DE* one-CL piece love send-PERF to school “The journalist sees the love which is dedicated to children has already been sent to the school.”

## 4 Discussion

This study adopted an eye-tracking paradigm to explore how semantics functions when Chinese native speakers process Chinese relative clauses by examining the semantic factors governing the word order variations. This study manipulated two syntactic variables and a semantic variable to explore when semantics is activated and interacts with syntax and how it affects syntax during the time course of Chinese relative clause processing. The results indicated that semantics was activated and interacted with syntax during the early and late processing stages of Chinese relative clauses. Moreover, the syntactic order of Chinese relative clauses was affected by semantic information of the head noun that the clause modified.

### 4.1 When semantics works

In previous studies, most models have taken their cue from linguistic theory that meaning is read off from syntactic structure (Chomsky, 1964; Jackendoff, 2007). However, it has been controversial whether semantic activation happens in the very initial phase across languages.

Syntax-first models argue that local syntactic structures are constructed based on syntactic category information and independent of lexical-semantic associative information, but not vice versa during the initial processing stage in the ERP paradigm (Friederici, 2002). They place less weight on the influence of lexical semantics during the build-up of syntactic structure (Kuperberg, 2007). Research on German and French sentence comprehension suggested syntactic information outweighed semantic information with failure to resolve syntactic category information “blocking” semantic integration processes (Hahne and Friederici, 2002; Friederici, 2011). This eye-tracking study, however, found that syntactic information and semantic information were both activated and interacted even from the very initial processing stage of Chinese sentences, as a counter to the syntax-first model. What’s more, this study also went against theories of the three-phase processing model (Friederici et al., 2004) and the extended argument dependency model (Bornkessel and Schlesewsky, 2006), which both argue no semantic information is activated in the initial processing phase and the initial stage involves purely syntax processing although they both challenge the sequential ordering of syntactic and semantic processes by feeding lexical information directly into both systems. However, the difference may be caused by methodological differences.

The findings of this study were in accord with previous studies in that semantic integration happens in the early processing stage (Yu and Zhang, 2008; Zhang et al., 2010; Wang et al., 2013). Ye et al. (2006) claimed that syntactic information and semantic information interacted in Chinese sentence processing, and the time course of this interplay is ahead of schedule compared with Indo-European. This scenario may be in connection with the specific properties of Chinese syntax, a generally non-inflectional language that differs significantly from Indo-European languages in both grammar and lexicons, where the syntactic category of lexicons can be identified not only via lexical semantics but also via grammatical morphology. In contrast, the lack of grammatical morphology in Chinese not only affects sentence comprehension and language processing in general (Luke et al., 2002; Li et al., 2004) but also leads to the conjecture that semantic analysis may play a more important part in Chinese language processing compared to Indo-European languages (e.g., Zhang et al., 2010, 2013; Yang et al., 2011). Yu and Zhang (2008) found that semantic integration proceeds even when syntactic category processing failed, and thus denied the functional primacy of syntactic category over semantic processes in Chinese sentence comprehension.

Zhang et al. (2010) added strong new evidence in support of the independence of semantic integration during Chinese sentence reading: semantic processing did not need a license from syntactic category processing for Chinese readers, even under the condition in which readers judged the sentence to be incorrect. From the perspective of linguistic typology, Huang et al. (2016) found syntactic information weighted much more than other information in such languages with stronger cues to syntactic structure as English and German, although the representational basis of language processing may be the same across languages with very different characteristics, with a fundamental distinction between the representation of syntactic information and semantic information.

**Figure 6:**
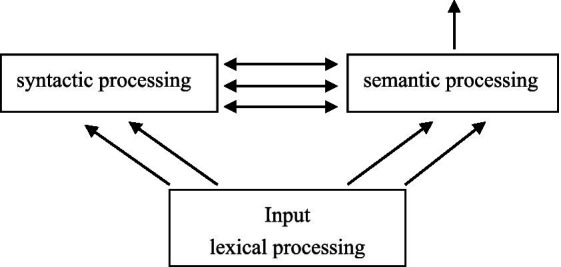
Syntactic-semantic information flow in the concurrent model.

Theoretically, the mechanism of Chinese sentence processing drawn from this study was in accordance with the claims of the concurrent model (Boland, 1997), which stated syntax and semantics were both activated and interacted even from the early processing stage. Specifically, when language comprehension happens, words are recognized and lexical information is fed directly into both syntactic and semantic systems in a concurrent manner. Syntactic and semantic processing were not rigidly ordered; rather, they ran concurrently, both receiving input from the lexicon, as illustrated in Figure 6. In the concurrent model, the semantic processor interacted with the syntactic processor and functioned by automatically generating all legal structures in parallel as each new word was perceived. The semantic system prevented the syntactic system from maintaining ungrammatical structures and guided reanalysis when necessary, so as to construct sentence representations.

In this study, numeral-classifier sequences’ distributional position in subject relative clauses was mediated by the abstractness of the head noun which the clause modified. Syntactic and semantic processing received the semantic information of the head noun concurrently. The semantic processor interacted with the syntactic processor and constructed legal syntactic representations automatically as each new word was perceived.

### 4.2 How semantics works

According to the eye-tracking results, object-extraction relative clauses and subject-extraction relative clauses had different preferences for numeral-classifier sequences’ distributional positions. Object-extraction relative clauses had a conjunction preference for outer modifier nominals, i.e., an object relative clause preceding a numeral-classifier sequence, forming “ORC+NCL + AN/CN” construction. However, subject-extraction relative clauses’ co-occurrence preference for numeral-classifier sequences’ distributional positions was affected by the abstractness of the head noun that the clause modified. The subject-extraction relative clauses which modified a concrete head noun had a co-occurrence preference for inner modifier nominals, with the numeral-classifier sequence preceding the subject relative clause and head noun, forming “NCL + SRC+ CN” construction, while the subject-extraction relative clause which modified an abstract head noun had a co-occurrence preference for outer modifier nominals, forming “SRC + NCL + AN” construction. It is a typical example in which syntactic order is driven not just by syntax processing itself, but also influenced by semantic accounts of the head noun that relative clause modified, and the influence is conversely embodied by the syntactic order, i.e., whether a numeral-classifier sequence is prior to or after the relative clause.

According to Zhang (2013), the inner modifier nominal was favored over the outer modifier nominal in relative clauses with human head nouns, which meant the overwhelming majority of relative clauses with human head nouns adopted “NCL + RC + human head noun” sequence. A similar pattern also existed for relative clauses with concrete head nouns, although the number was lower, while the outer modifier nominal was favored over the inner modifier nominal if the head NP referred to an abstract entity. In short, for abstract head nouns, the outer modifier nominal was favored, while for non-abstract head nouns, the inner modifier nominal was preferred. In addition, research also suggested semantic accounts such as animacy and discourse salience of head nouns both influenced the distribution of numeral-classifier sequence in relative clauses (Wu et al., 2011; He and Chen, 2013).

More generally, previous research on relative clause and word order variation has identified most of the potential factors which may affect the choice of one construction over the other. Goldberg (1997) proposed that lexical semantics played a significant role in constructions. Lexicon made the meaning of construction more specific and exerted a dynamic force upon constructions. Pollard and Sag (1994) put forward Head-Driven Phrase Structure Grammar. According to the theory, words carried rich syntactic and semantic information, which largely determined the syntactic and semantic structure of the clause in which they were located. The reason why sentences showed different syntactic and semantic structures was precisely because the key words contained in them were different.

Lu (2004) observed the relation among syntactic construction, conceptual structure, and sentence understanding, and illustrated that what kind of syntactic construction a specific sequence formed or what it meant exactly depended on the lexical meanings of the parts of the sequence. Li and Gao’s (2012) research was administered to examine the effects of syntactic, semantic, and pragmatic factors on Chinese clause processing. The experiments were carried out to compare Chinese native speakers with Korean and Japanese CFL learners. The results showed the semantic factor had stronger effects than the syntactic and pragmatic ones. By examining all possible word order variations in the world languages through the World Atlas of Language Structures, Luuk (2015) found that in languages with fixed word order, semantics had probably a minor role in it, while in languages with (relatively) free syntactic order, semantics (especially pragmatics) was responsible for at least some ordering principles.

Evidence from corpora also showed the animacy of the RC head noun, for example, is a strong indicator of RC type (Lau and Tanaka, 2021). In adult production, SRCs are more likely to have an animate head, and ORCs an inanimate head (Roland et al., 2007). Sentence continuation research also shows the effects of head animacy. Native English speakers tend to complete a given RC with an animate head as an SRC, and one with an inanimate head as an ORC (Gennari and MacDonald, 2008), which is in accordance with the findings in this study. Of particular interest is why semantic information influences syntactic order, i.e., why the abstractness of head nouns affects the distributional position of numeral-classifier sequences in this study. The following part will explore the issue from different perspectives.

#### 4.2.1 Truth value semantics of the syntactic components

This study demonstrated that subject-extraction relative clauses that modified a concrete head noun and an abstract head noun had different preferences for numeral-classifier sequences’ distributional positions. The divergent preference may be rooted in the semantic conditions of the numeral “一” (*yi*, “one”) in “NCL + SRC + CN” and “SRC + NCL + AN” constructions.

According to cognitive linguistics, the connection between words (or expressions) and reality falls into the truth-conditional semantic condition and non-truth value condition (Kearns, 2011). In the Chinese language, the semantic conditions of the numeral “一” (*yi*, “one”) vary dramatically with the contexts in which it is located. It may convey truth value meaning (logic meaning) or non-truth value meaning (non-logic meaning), influenced by syntactic and semantic variations. In general, the numeral “一” (*yi*, “one”) which expresses the meaning of logical quantity can be labeled as a truth-value numeral. Every truth-value numeral, in nature, can be freely replaced by other numerals without affecting the syntactic and grammatical function of the sentence, while a numeral that does not convey logic quantity falls into the category of non-truth-value ones.

Based on the distinction, truth-value classifier phrases (such as “一个小偷,” *yi-ge xiaotou*, “one thief”) and non-truth value classifier phrases (such as “一片爱心,” *yi-pian aixin*, “one piece of love”) are derived in the study. For the former “一个小偷” (*yi-ge xiaotou*, “one thief”), the numeral slot is not limited to the numeral “一” (*yi*, “one”). Instead, it can be replaced with other numerals, such as “三个小偷” (*san-ge xiaotou*, “three thieves”), while for the latter “一片爱心” (*yi-pian aixin*, “one piece of love”), “一” (*yi*, “one”) does not carry the truth value meaning, and the numeral slot is exclusively restricted to “一” (*yi*, “one”). Other numerals are ungrammatical to be fillers, such as “三片爱心” (*san-pian aixin*, “three pieces of love”). In this sense, “一” (*yi*, “one”) in “一个穿着黑衣的小偷” (*yi-ge chuanzhe heiyi de xiaotou*, “a thief in black”) acted as the numeral of a NumP, while “一” (*yi*, “one”) in “一片献给孩子的爱心” (*yi-pian xiangei haizi de aixin*, “the love which is dedicated to children”) is not served as a numeral, but as the specifier of a determiner phrase.

Essentially, the numeral-classifier sequence “一片” (*yi-pian*, “one piece”) in the construction “献给孩子的一片爱心” (*xiangei haizi de yi-pian aixin*, “the love which is dedicated to children”) is a functional and pragmatic marker rather than a quantity-measuring classifier, with its rhetorical function and prosody function being derived and salient, as a means to separate and activate the specific “爱心” (*aixin*, “love”) from the set. Acting as a functional marker, the numeral-classifier sequence “一片” (*yi-pian*, “one piece”) has a close connection with its head noun, so that it must be located in the syntactic position as close to its head noun as possible. It is not appropriate to split the phrase “一片爱心” (*yi-pian aixin*, “one piece of love”), and any filler between the two (such as a relative clause) will be an unacceptable interruption. In this sense, “一片献给孩子的爱心” (*yi-pian xiangei haizi de aixin*, “the love which is dedicated to children”) is less acceptable than “献给孩子的一片爱心” (*xiangei haizi de yi-pian aixin*, “the love which is dedicated to children”), because for the former, the subject-extraction relative clause, acting as a syntactic interruption, is inserted between a numeral-classifier sequence and its head noun. Naturally, participants consumed more cognitive processing resources and took longer regression path durations on “NCL + RC + AN” construction, such as “记者看到一片献给孩子的爱心已送到了学校” (*jizhe kandao yi-pian xiangei haizi de aixin yi songdao le xuexiao*, “The journalist sees the love which is dedicated to children has already been sent to the school”) and “记者看到一片社会奉献的爱心已送到了学校” (*jizhe kandao yi-pian shehui fengxian de aixin yi songdao le xuexiao*, “The journalist sees the love which the society dedicated has already been sent to the school”), while their processing load was lower on their counterpart constructions “RC + NCL + AN,” such as “记者看到献给孩子的一片爱心已送到了学校” (*jizhe kandao xiangei haizi de yi-pian aixin yi songdao le xuexiao*, “The journalist sees the love which is dedicated to children has already been sent to the school”) and “记者看到社会奉献的一片爱心已送到了学校” (*jizhe kandao shehui fengxian de yi-pian aixin yi songdao le xuexiao*, “The journalist sees the love which the society dedicated has already been sent to the school”).

#### 4.2.2 The semantic compatibility of numeral-classifier sequence and The head noun

In this study, subject-extraction relative clauses with different head nouns had different conjunction preferences for the distributional position of numeral-classifier sequences, which can be accounted for by the semantic compatibility of the numeral-classifier sequence and its modified noun. In cognitive linguistics, dual-coding theory (Paivio, 1991) argues that concrete words can activate information in a nonverbal “imagistic” system through referential connections. Superior associative connections and the use of mental imagery both contribute to the processing advantage of concrete words over abstract words (West and Holcomb, 2000). For human cognition, there is a tendency to concretize abstract nouns (Langacker, 2000).

Take “一片爱心” (*yi-pian aixin*, “one piece of love”) as an example, the head noun “爱心” (*aixin*, “love”) is boundless and abstract, which can neither be quantified nor outlined in shape, while its NCL modifier “一片” (*yi-pian*, “one piece”) is usually adopted to modify concrete and bounded nouns such as “树叶” (*shuye*, “leaf”) and “面包” (*mianbao*, “bread”). In linguistic world, humans frequently use a concrete classifier to give an abstract term a concrete sense, to concretize the abstract noun, so that the boundless and shapeless abstract noun can be concreted into conceptual and semantic “boundness.” In the study, “爱心” (*aixin*, “love”) becomes bounded and shaped with the help of the concrete numeral-classifier sequence “一片” (*yi-pian*, “one piece”). “一片爱心” (*yi-pian aixin*, “one piece of love”) is thus “structuralized or grammaticalized into a temporary syntactic structure (with highly fixed form-meaning sentence pattern) in linguistic world” (Langacker, 1991). Accordingly, the temporary association and the semantic compatibility between the numeral-classifier sequence “一片” (*yi-pian*, “one piece”) and the modified head noun “爱心” (*aixin*, “love”) are quite weak. The flexibility of the syntactic closeness between the two is limited. Consequently, the numeral-classifier sequence “一片” (*yi-pian*, “one piece”) must be located in the syntactic position as close to the head noun as possible. The weak connection and compatibility between the numeral-classifier sequence “一片” (*yi-pian*, “one piece”) and the modified noun “爱心” (*aixin*, “love”) will be threatened if an additional element, like a relative clause, is placed between them. An example of this would be “一片献给孩子的爱心” (*yi-pian xiangei haizi de aixin*, “the love which is dedicated to children”), which is less acceptable than “献给孩子的一片爱心” (*xiangei haizi de yi-pian aixin*, “the love which is dedicated to children”).

#### 4.2.3 Discourse functions of outer and inner modifier nominal constructions

According to Ming and Chen (2010), outer modifier nominal and inner modifier nominal constructions are deployed in discourse to serve different discourse purposes. Specifically, outer modifier nominal construction is mainly used in connection with abstract entities with low discourse salience, serving the function of identifying the new head NP. In contrast, inner modifier nominal construction is mainly used in conjunction with concrete entities (human and concrete objects) with high discourse salience, serving the function of characterization.

In this study, both subject-extraction relative clauses and object-extraction relative clauses with abstract head nouns have a conjunction preference for outer modifier nominal, which is in accordance with the findings of Ming and Chen (2010), i.e., outer modifier nominal is mainly used in connection with abstract entities with low discourse salience, as in “献给孩子的一片爱心” (*xiangei haizi de yi-pian aixin*, “the love which is dedicated to children”), serving the discourse function of identifying the new head noun “一片爱心” (*yi-pian aixin*, “one piece of love”) from other given information. In contrast, inner modifier nominal is mainly used in conjunction with concrete entities with high discourse salience, as in “记者看到一个穿着黑衣的小偷已逃离了现场” (*jizhe kandao yi-ge chuanzhe heiyi de xiaotou yi taoli le xianchang,* “The journalist sees a thief in black has already fled the scene”), fulfilling the discourse function of characterization and giving a more detailed description of the head noun “小偷” (*xiaotou*, “thief”).

## 5 Conclusion

This study provided new evidence for when and how semantics functions in language processing. The mechanism drawn from this eye-tracking task is a necessary and beneficial complement to previous findings of ERP paradigms.

First, this study confirmed the activation and interaction of syntax and semantics in Chinese RC-embedded sentences during the early and late processing stages, which supported the claims of the concurrent model that syntactic and semantic systems both make use of the information of lexical entry in a concurrent manner so as to construct sentence representations interactively even from the early processing stages.

Second, this study confirmed that lexical semantic factors governed the word order variation that is possible for a subset of relative clauses in Mandarin Chinese. Object-extraction relative clauses and subject-extraction relative clauses had different preferences for the numeral-classifier sequence distributional positions during on-line sentence comprehension. Object-extraction relative clauses had a co-occurrence preference for outer modifier nominal, i.e., an object relative clause preceding the numeral-classifier sequence and the head noun. However, the numeral-classifier sequence’s distributional position in subject-extraction relative clauses was affected by the semantic factor, i. e. the abstractness of the head noun that the subject-extraction relative clause modified. Specifically, the subject-extraction relative clause which modified a concrete head noun had a co-occurrence preference for inner modifier nominal, with the numeral-classifier sequence preceding the subject relative clause and the head noun, forming constructions as “NCL + SRC+ CN,” while the subject-extraction relative clause which modified an abstract head noun had a co-occurrence preference for outer modifier nominal, forming such a construction as “SRC + NCL + AN,” indicating the change of word order is accompanied and influenced by the change of semantics, and the influence was conversely embodied by the syntactic order, consequently forming a “syntax-semantics” interface.

Previous research suggests that the processing of the Chinese language may be more intricate. It is possible that distinct processing mechanisms apply to different syntactic forms. However, there may be methodological limitations to the study’s conclusions, which would require further corpus-based analysis and empirical tests under the ERP paradigm for verification in further studies. It would be fascinating to explore how cross-linguistic differences arise and whether these findings from the Chinese language can be applied to typologically distinct languages.

## Data availability statement

The original contributions presented in the study are included in the article/Supplementary material, further inquiries can be directed to the corresponding author.

## Ethics statement

The studies involving human participants were reviewed and approved by the Academic Committee of Nanjing University of Posts and Telecommunications. The participants provided their written informed consent to participate in this study.

## Author contributions

YL: Data curation, Resources, Writing – original draft, Writing – review & editing. CN: Methodology, Writing – review & editing.
