# Supplementary material for: How semantics works in Chinese relative clause processing: insights from eye tracking
Source: Front Psychol. 2024 Feb 19;15:1294132. doi: 10.3389/fpsyg.2024.1294132 (PMC10910112; doi:10.3389/fpsyg.2024.1294132)
Supplement: Supplementary file 2 [file Table_2.docx]

数据统计原表

行为数据：

**Table 。。。** Summary of the reaction time and acceptability of eight conditions in Experiment 3

| **Type** | **Conditions** | **Illustrations** | **Examples** | **N** | **Reaction Time** | **Acceptability** |
| --- | --- | --- | --- | --- | --- | --- |
| **IMN** | A1B1C1 | CL+SRC+CN | 记者看到一个穿着黑衣的小偷已逃离了现场。 | 35 | 3390.486±473.641 | 4.778±0. 155 |
|  | A1B1C2 | CL+ORC+CN | 记者看到一个子弹击中的小偷已逃离了现场。 | 35 | 3815.486±588.944 | 3.715±0.418 |
|  | A1B2C1 | CL+SRC+AN | 记者看到一片献给孩子的爱心已送到了学校。 | 35 | 3735.971±501.847 | 3.836±0.490 |
|  | A1B2C2 | CL+ORC+AN | 记者看到一片社会奉献的爱心已送到了学校。 | 35 | 3904.886±553.686 | 3.517±0.482 |
| **OMN** | A2B1C1 | SRC+CL+CN | 记者看到穿着黑衣的一个小偷已逃离了现场。 | 35 | 3494.429±619.153 | 4.312±0.414 |
|  | A2B1C2 | ORC+CL+CN | 记者看到子弹击中的一个小偷已逃离了现场。 | 35 | 3588.057±567.836 | 4.150±0.541 |
|  | A2B2C1 | SRC+CL+ AN | 记者看到献给孩子的一片爱心已送到了学校。 | 35 | 3561.457±388.444 | 4.519±0.384 |
|  | A2B2C2 | ORC+CL+ AN | 记者看到社会奉献的一片爱心已送到了学校。 | 35 | 3662.371±650.682 | 3.964±0.651 |

注：Traditionally, inferential statistics of ANOVAs should report such results both by participants, i.e., *F*1; and by items, i.e., *F*2 放在实验1数据后

|  |  |  |
| --- | --- | --- |

Table. main effects and interactive effects of sources in Experiment 3

| **Source(Type)** | **Reaction Time** | | | **Acceptability** | | |
| --- | --- | --- | --- | --- | --- | --- |
|  | **F(df)** | ***p*** | **η^2^_p_(partial eta．squared)** | **F(df)** | ***P*** | **η^2^_p_** |
| **A(IMN/OMN)** | F_1_(1,34)= 3.677 | .064 | .098 | F_1_(1,34)=27.056 | .000 | .443 |
|  | F_2_(1,31)= 3.898 | .057 | .112 | F_2_(1,31)=20.510 | .000 | .398 |
| **B(CN/AN)** | F_1_(1,34)= 6.121 | .019 | .153 | F_1_(1,34)=22.535 | .000 | .399 |
|  | F_2_(1,31)= 4.823 | .036 | .135 | F_2_(1,31)=25.454 | .000 | .451 |
| **C(SRC/ORC)** | F_1_(1,34)= 8.821 | .005 | .206 | F_1_(1,34)=87.611 | .000 | .720 |
|  | F_2_(1,31)= 9.739 | .004 | .239 | F_2_(1,31)=90.393 | .000 | .745 |
| **A * B** | F_1_(1,34)= 1.301 | .262 | .037 | F_1_(1,34)=20.077 | .000 | .371 |
|  | F_2_(1,31)= .846 | .365 | .027 | F_2_(1,31)=15.243 | .000 | .330 |
| **A * C** | F_1_(1,34)= 2.909 | .097 | .079 | F_1_(1,34)=11.228 | .002 | .248 |
|  | F_2_(1,31)= 1.334 | .257 | .041 | F_2_(1,31)=9.176 | .005 | .228 |
| **B * C** | F_1_(1,34)= .771 | .386 | .022 | F_1_(1,34)=3.882 | .057 | .102 |
|  | F_2_(1,31)= 1.996 | .168 | .060 | F_2_(1,31)=6.105 | .019 | .165 |
| **A * B * C** | F_1_(1,34)= .853 | .362 | .024 | F_1_(1,34)=30.070 | .000 | .469 |
|  | F_2_(1,31)= 1.089 | .305 | .034 | F_2_(1,31)=37.418 | .000 | .547 |

**Table 。。。** Summary of the eye-tracking results of eight conditions in Experiment 3

| **Type** | **Illustrations** | **Examples** | **N** | **First Stage** | | **Late Stage** |
| --- | --- | --- | --- | --- | --- | --- |
|  |  |  |  | **1st Fixation durations** | **Gaze durations** | **Regression durations** |
| **IMN** | CL+SRC+CN | 记者看到一个穿着黑衣的小偷已逃离了现场。 | 35 | 206.057±41.410 | 747.657±62.367 | 1395.114±127.756 |
|  | CL+ORC+CN | 记者看到一个子弹击中的小偷已逃离了现场。 | 35 | 225.343±50.667 | 1212.686±158.241 | 1563.914±178.354 |
|  | CL+SRC+AN | 记者看到一片献给孩子的爱心已送到了学校。 | 35 | 232.029±41.273 | 1197.257±129.562 | 1475.771±145.573 |
|  | CL+ORC+AN | 记者看到一片社会奉献的爱心已送到了学校。 | 35 | 239.086±41.067 | 1358.400±110.439 | 1602.171±156.011 |
| **OMN** | SRC+CL+CN | 记者看到穿着黑衣的一个小偷已逃离了现场。 | 35 | 242.514±46.968 | 1004.600±98.863 | 1473.143±97.237 |
|  | ORC+CL+CN | 记者看到子弹击中的一个小偷已逃离了现场。 | 35 | 236.514±46.084 | 924.457±111.343 | 1496.914±133.208 |
|  | SRC+CL+ AN | 记者看到献给孩子的一片爱心已送到了学校。 | 35 | 212.371±35.874 | 1098.343±87.061 | 1400.543±113.598 |
|  | ORC+CL+ AN | 记者看到社会奉献的一片爱心已送到了学校。 | 35 | 248.771±66.422 | 816.857±103.284 | 1515.143±133.160 |

**Results of the acceptability task**

Participants’ behavioral data were recorded which showed that the average ACC was 97.430% for the advanced group and 94.544% for the intermediate group, suggesting that participants were actively attending the experiment. The descriptive statistic results of acceptability concerning four conditions among the relatively advanced level of participants (n=63) were presented in Table 。。。. Obviously, the mean acceptability of 5-point Likert scale among those four conditions

were 4.925, 3.988, 4.841 and 2.350, respectively, which could be roughly interpreted as the fact that “condition a” was the most acceptable, “condition c” the second, “condition b” the third, and “condition d” the lest acceptable.

接受度：(用这个表作为阐释的模板，每个指标都是按照句法指标一段 语义指标一段 句法语义交互一段来阐释数据）

|  | Semantic Level | | | | | Syntactic Level | | | |
| --- | --- | --- | --- | --- | --- | --- | --- | --- | --- |
|  |  |  | t(df) | sig. | Cohen's d (effect size) |  | t(df) | sig. | Cohen's d (effect size) |
| **IMN** | A1B1C1 | CL+SRC+CN |  |  |  | CL+SRC+N | 7.887(125.015) | .000 |  |
|  | A1B2C1 | CL+SRC+AN |  |  |  |  |  |  |  |
|  | A1B1C2 | CL+ORC+CN |  |  |  | CL+ORC+N |  |  |  |
|  | A1B2C2 | CL+ORC+AN |  |  |  |  |  |  |  |
| **OMN** | A2B1C1 | SRC+CL+CN |  |  |  | SRC+CL+N | 4.121(121.699) | .000 |  |
|  | A2B2C1 | SRC+CL+ AN |  |  |  |  |  |  |  |
|  | A2B1C2 | ORC+CL+CN |  |  |  | ORC+CL+N |  |  |  |
|  | A2B2C2 | ORC+CL+ AN |  |  |  |  |  |  |  |

接受度：

8个条件的主效应和交互效应（被试内）：（已统计过的数据加上灰色阴影表示）

| **描述性统计量** | | | |
| --- | --- | --- | --- |
|  | 均值 | 标准 偏差 | N |
| A1B1C1 | 4.7780 | .15541 | 35 |
| A1B1C2 | 3.7151 | .41780 | 35 |
| A1B2C1 | 3.8363 | .49020 | 35 |
| A1B2C2 | 3.5171 | .48208 | 35 |
| A2B1C1 | 4.3123 | .41383 | 35 |
| A2B1C2 | 4.1500 | .54057 | 35 |
| A2B2C1 | 4.5186 | .38375 | 35 |
| A2B2C2 | 3.9643 | .65076 | 35 |

| **Mauchly 的球形度检验^a^** | | | | | | | |
| --- | --- | --- | --- | --- | --- | --- | --- |
| 度量: MEASURE_1 | | | | | | | |
| 主体内效应 | Mauchly 的 W | 近似卡方 | df | Sig. | Epsilon^b^ | | |
|  |  |  |  |  | Greenhouse-Geisser | Huynh-Feldt | 下限 |
| A | 1.000 | .000 | 0 | . | 1.000 | 1.000 | 1.000 |
| B | 1.000 | .000 | 0 | . | 1.000 | 1.000 | 1.000 |
| C | 1.000 | .000 | 0 | . | 1.000 | 1.000 | 1.000 |
| A * B | 1.000 | .000 | 0 | . | 1.000 | 1.000 | 1.000 |
| A * C | 1.000 | .000 | 0 | . | 1.000 | 1.000 | 1.000 |
| B * C | 1.000 | .000 | 0 | . | 1.000 | 1.000 | 1.000 |
| A * B * C | 1.000 | .000 | 0 | . | 1.000 | 1.000 | 1.000 |
| 检验零假设，即标准正交转换因变量的误差协方差矩阵与一个单位矩阵成比例。 | | | | | | | |
| a. 设计 : 截距  主体内设计: A + B + C + A * B + A * C + B * C + A * B * C | | | | | | | |
| b. 可用于调整显著性平均检验的自由度。 在"主体内效应检验"表格中显示修正后的检验。 | | | | | | | |

| **主体内效应的检验** | | | | | | | | | |
| --- | --- | --- | --- | --- | --- | --- | --- | --- | --- |
| 度量: MEASURE_1 | | | | | | | | | |
| 源 | | III 型平方和 | df | 均方 | F | Sig. | 偏 Eta 方 | 非中心 参数 | 观测到的幂^a^ |
| A | 采用的球形度 | 5.280 | 1 | 5.280 | 27.056 | .000 | .443 | 27.056 | .999 |
|  | Greenhouse-Geisser | 5.280 | 1.000 | 5.280 | 27.056 | .000 | .443 | 27.056 | .999 |
|  | Huynh-Feldt | 5.280 | 1.000 | 5.280 | 27.056 | .000 | .443 | 27.056 | .999 |
|  | 下限 | 5.280 | 1.000 | 5.280 | 27.056 | .000 | .443 | 27.056 | .999 |
| 误差 (A) | 采用的球形度 | 6.635 | 34 | .195 |  |  |  |  |  |
|  | Greenhouse-Geisser | 6.635 | 34.000 | .195 |  |  |  |  |  |
|  | Huynh-Feldt | 6.635 | 34.000 | .195 |  |  |  |  |  |
|  | 下限 | 6.635 | 34.000 | .195 |  |  |  |  |  |
| B | 采用的球形度 | 5.480 | 1 | 5.480 | 22.535 | .000 | .399 | 22.535 | .996 |
|  | Greenhouse-Geisser | 5.480 | 1.000 | 5.480 | 22.535 | .000 | .399 | 22.535 | .996 |
|  | Huynh-Feldt | 5.480 | 1.000 | 5.480 | 22.535 | .000 | .399 | 22.535 | .996 |
|  | 下限 | 5.480 | 1.000 | 5.480 | 22.535 | .000 | .399 | 22.535 | .996 |
| 误差 (B) | 采用的球形度 | 8.267 | 34 | .243 |  |  |  |  |  |
|  | Greenhouse-Geisser | 8.267 | 34.000 | .243 |  |  |  |  |  |
|  | Huynh-Feldt | 8.267 | 34.000 | .243 |  |  |  |  |  |
|  | 下限 | 8.267 | 34.000 | .243 |  |  |  |  |  |
| C | 采用的球形度 | 19.268 | 1 | 19.268 | 87.611 | .000 | .720 | 87.611 | 1.000 |
|  | Greenhouse-Geisser | 19.268 | 1.000 | 19.268 | 87.611 | .000 | .720 | 87.611 | 1.000 |
|  | Huynh-Feldt | 19.268 | 1.000 | 19.268 | 87.611 | .000 | .720 | 87.611 | 1.000 |
|  | 下限 | 19.268 | 1.000 | 19.268 | 87.611 | .000 | .720 | 87.611 | 1.000 |
| 误差 (C) | 采用的球形度 | 7.477 | 34 | .220 |  |  |  |  |  |
|  | Greenhouse-Geisser | 7.477 | 34.000 | .220 |  |  |  |  |  |
|  | Huynh-Feldt | 7.477 | 34.000 | .220 |  |  |  |  |  |
|  | 下限 | 7.477 | 34.000 | .220 |  |  |  |  |  |
| A * B | 采用的球形度 | 5.890 | 1 | 5.890 | 20.077 | .000 | .371 | 20.077 | .992 |
|  | Greenhouse-Geisser | 5.890 | 1.000 | 5.890 | 20.077 | .000 | .371 | 20.077 | .992 |
|  | Huynh-Feldt | 5.890 | 1.000 | 5.890 | 20.077 | .000 | .371 | 20.077 | .992 |
|  | 下限 | 5.890 | 1.000 | 5.890 | 20.077 | .000 | .371 | 20.077 | .992 |
| 误差 (A*B) | 采用的球形度 | 9.974 | 34 | .293 |  |  |  |  |  |
|  | Greenhouse-Geisser | 9.974 | 34.000 | .293 |  |  |  |  |  |
|  | Huynh-Feldt | 9.974 | 34.000 | .293 |  |  |  |  |  |
|  | 下限 | 9.974 | 34.000 | .293 |  |  |  |  |  |
| A * C | 采用的球形度 | 1.937 | 1 | 1.937 | 11.228 | .002 | .248 | 11.228 | .902 |
|  | Greenhouse-Geisser | 1.937 | 1.000 | 1.937 | 11.228 | .002 | .248 | 11.228 | .902 |
|  | Huynh-Feldt | 1.937 | 1.000 | 1.937 | 11.228 | .002 | .248 | 11.228 | .902 |
|  | 下限 | 1.937 | 1.000 | 1.937 | 11.228 | .002 | .248 | 11.228 | .902 |
| 误差 (A*C) | 采用的球形度 | 5.866 | 34 | .173 |  |  |  |  |  |
|  | Greenhouse-Geisser | 5.866 | 34.000 | .173 |  |  |  |  |  |
|  | Huynh-Feldt | 5.866 | 34.000 | .173 |  |  |  |  |  |
|  | 下限 | 5.866 | 34.000 | .173 |  |  |  |  |  |
| B * C | 采用的球形度 | .541 | 1 | .541 | 3.882 | .057 | .102 | 3.882 | .482 |
|  | Greenhouse-Geisser | .541 | 1.000 | .541 | 3.882 | .057 | .102 | 3.882 | .482 |
|  | Huynh-Feldt | .541 | 1.000 | .541 | 3.882 | .057 | .102 | 3.882 | .482 |
|  | 下限 | .541 | 1.000 | .541 | 3.882 | .057 | .102 | 3.882 | .482 |
| 误差 (B*C) | 采用的球形度 | 4.740 | 34 | .139 |  |  |  |  |  |
|  | Greenhouse-Geisser | 4.740 | 34.000 | .139 |  |  |  |  |  |
|  | Huynh-Feldt | 4.740 | 34.000 | .139 |  |  |  |  |  |
|  | 下限 | 4.740 | 34.000 | .139 |  |  |  |  |  |
| A * B * C | 采用的球形度 | 5.643 | 1 | 5.643 | 30.070 | .000 | .469 | 30.070 | 1.000 |
|  | Greenhouse-Geisser | 5.643 | 1.000 | 5.643 | 30.070 | .000 | .469 | 30.070 | 1.000 |
|  | Huynh-Feldt | 5.643 | 1.000 | 5.643 | 30.070 | .000 | .469 | 30.070 | 1.000 |
|  | 下限 | 5.643 | 1.000 | 5.643 | 30.070 | .000 | .469 | 30.070 | 1.000 |
| 误差 (A*B*C) | 采用的球形度 | 6.381 | 34 | .188 |  |  |  |  |  |
|  | Greenhouse-Geisser | 6.381 | 34.000 | .188 |  |  |  |  |  |
|  | Huynh-Feldt | 6.381 | 34.000 | .188 |  |  |  |  |  |
|  | 下限 | 6.381 | 34.000 | .188 |  |  |  |  |  |
| a. 使用 alpha 的计算结果 = .05 | | | | | | | | | |

8个条件的主效应和交互效应（项目内）：

| **Mauchly 的球形度检验^a^** | | | | | | | |
| --- | --- | --- | --- | --- | --- | --- | --- |
| 度量: MEASURE_1 | | | | | | | |
| 主体内效应 | Mauchly 的 W | 近似卡方 | df | Sig. | Epsilon^b^ | | |
|  |  |  |  |  | Greenhouse-Geisser | Huynh-Feldt | 下限 |
| A | 1.000 | .000 | 0 | . | 1.000 | 1.000 | 1.000 |
| B | 1.000 | .000 | 0 | . | 1.000 | 1.000 | 1.000 |
| C | 1.000 | .000 | 0 | . | 1.000 | 1.000 | 1.000 |
| A * B | 1.000 | .000 | 0 | . | 1.000 | 1.000 | 1.000 |
| A * C | 1.000 | .000 | 0 | . | 1.000 | 1.000 | 1.000 |
| B * C | 1.000 | .000 | 0 | . | 1.000 | 1.000 | 1.000 |
| A * B * C | 1.000 | .000 | 0 | . | 1.000 | 1.000 | 1.000 |
| 检验零假设，即标准正交转换因变量的误差协方差矩阵与一个单位矩阵成比例。 | | | | | | | |
| a. 设计 : 截距  主体内设计: A + B + C + A * B + A * C + B * C + A * B * C | | | | | | | |
| b. 可用于调整显著性平均检验的自由度。 在"主体内效应检验"表格中显示修正后的检验。 | | | | | | | |

| **主体内效应的检验** | | | | | | | | | |
| --- | --- | --- | --- | --- | --- | --- | --- | --- | --- |
| 度量: MEASURE_1 | | | | | | | | | |
| 源 | | III 型平方和 | df | 均方 | F | Sig. | 偏 Eta 方 | 非中心 参数 | 观测到的幂^a^ |
| A | 采用的球形度 | 4.460 | 1 | 4.460 | 20.510 | .000 | .398 | 20.510 | .992 |
|  | Greenhouse-Geisser | 4.460 | 1.000 | 4.460 | 20.510 | .000 | .398 | 20.510 | .992 |
|  | Huynh-Feldt | 4.460 | 1.000 | 4.460 | 20.510 | .000 | .398 | 20.510 | .992 |
|  | 下限 | 4.460 | 1.000 | 4.460 | 20.510 | .000 | .398 | 20.510 | .992 |
| 误差 (A) | 采用的球形度 | 6.741 | 31 | .217 |  |  |  |  |  |
|  | Greenhouse-Geisser | 6.741 | 31.000 | .217 |  |  |  |  |  |
|  | Huynh-Feldt | 6.741 | 31.000 | .217 |  |  |  |  |  |
|  | 下限 | 6.741 | 31.000 | .217 |  |  |  |  |  |
| B | 采用的球形度 | 6.085 | 1 | 6.085 | 25.454 | .000 | .451 | 25.454 | .998 |
|  | Greenhouse-Geisser | 6.085 | 1.000 | 6.085 | 25.454 | .000 | .451 | 25.454 | .998 |
|  | Huynh-Feldt | 6.085 | 1.000 | 6.085 | 25.454 | .000 | .451 | 25.454 | .998 |
|  | 下限 | 6.085 | 1.000 | 6.085 | 25.454 | .000 | .451 | 25.454 | .998 |
| 误差 (B) | 采用的球形度 | 7.411 | 31 | .239 |  |  |  |  |  |
|  | Greenhouse-Geisser | 7.411 | 31.000 | .239 |  |  |  |  |  |
|  | Huynh-Feldt | 7.411 | 31.000 | .239 |  |  |  |  |  |
|  | 下限 | 7.411 | 31.000 | .239 |  |  |  |  |  |
| C | 采用的球形度 | 17.114 | 1 | 17.114 | 90.393 | .000 | .745 | 90.393 | 1.000 |
|  | Greenhouse-Geisser | 17.114 | 1.000 | 17.114 | 90.393 | .000 | .745 | 90.393 | 1.000 |
|  | Huynh-Feldt | 17.114 | 1.000 | 17.114 | 90.393 | .000 | .745 | 90.393 | 1.000 |
|  | 下限 | 17.114 | 1.000 | 17.114 | 90.393 | .000 | .745 | 90.393 | 1.000 |
| 误差 (C) | 采用的球形度 | 5.869 | 31 | .189 |  |  |  |  |  |
|  | Greenhouse-Geisser | 5.869 | 31.000 | .189 |  |  |  |  |  |
|  | Huynh-Feldt | 5.869 | 31.000 | .189 |  |  |  |  |  |
|  | 下限 | 5.869 | 31.000 | .189 |  |  |  |  |  |
| A * B | 采用的球形度 | 4.695 | 1 | 4.695 | 15.243 | .000 | .330 | 15.243 | .966 |
|  | Greenhouse-Geisser | 4.695 | 1.000 | 4.695 | 15.243 | .000 | .330 | 15.243 | .966 |
|  | Huynh-Feldt | 4.695 | 1.000 | 4.695 | 15.243 | .000 | .330 | 15.243 | .966 |
|  | 下限 | 4.695 | 1.000 | 4.695 | 15.243 | .000 | .330 | 15.243 | .966 |
| 误差 (A*B) | 采用的球形度 | 9.549 | 31 | .308 |  |  |  |  |  |
|  | Greenhouse-Geisser | 9.549 | 31.000 | .308 |  |  |  |  |  |
|  | Huynh-Feldt | 9.549 | 31.000 | .308 |  |  |  |  |  |
|  | 下限 | 9.549 | 31.000 | .308 |  |  |  |  |  |
| A * C | 采用的球形度 | 1.747 | 1 | 1.747 | 9.176 | .005 | .228 | 9.176 | .835 |
|  | Greenhouse-Geisser | 1.747 | 1.000 | 1.747 | 9.176 | .005 | .228 | 9.176 | .835 |
|  | Huynh-Feldt | 1.747 | 1.000 | 1.747 | 9.176 | .005 | .228 | 9.176 | .835 |
|  | 下限 | 1.747 | 1.000 | 1.747 | 9.176 | .005 | .228 | 9.176 | .835 |
| 误差 (A*C) | 采用的球形度 | 5.903 | 31 | .190 |  |  |  |  |  |
|  | Greenhouse-Geisser | 5.903 | 31.000 | .190 |  |  |  |  |  |
|  | Huynh-Feldt | 5.903 | 31.000 | .190 |  |  |  |  |  |
|  | 下限 | 5.903 | 31.000 | .190 |  |  |  |  |  |
| B * C | 采用的球形度 | .571 | 1 | .571 | 6.105 | .019 | .165 | 6.105 | .668 |
|  | Greenhouse-Geisser | .571 | 1.000 | .571 | 6.105 | .019 | .165 | 6.105 | .668 |
|  | Huynh-Feldt | .571 | 1.000 | .571 | 6.105 | .019 | .165 | 6.105 | .668 |
|  | 下限 | .571 | 1.000 | .571 | 6.105 | .019 | .165 | 6.105 | .668 |
| 误差 (B*C) | 采用的球形度 | 2.899 | 31 | .094 |  |  |  |  |  |
|  | Greenhouse-Geisser | 2.899 | 31.000 | .094 |  |  |  |  |  |
|  | Huynh-Feldt | 2.899 | 31.000 | .094 |  |  |  |  |  |
|  | 下限 | 2.899 | 31.000 | .094 |  |  |  |  |  |
| A * B * C | 采用的球形度 | 5.644 | 1 | 5.644 | 37.418 | .000 | .547 | 37.418 | 1.000 |
|  | Greenhouse-Geisser | 5.644 | 1.000 | 5.644 | 37.418 | .000 | .547 | 37.418 | 1.000 |
|  | Huynh-Feldt | 5.644 | 1.000 | 5.644 | 37.418 | .000 | .547 | 37.418 | 1.000 |
|  | 下限 | 5.644 | 1.000 | 5.644 | 37.418 | .000 | .547 | 37.418 | 1.000 |
| 误差 (A*B*C) | 采用的球形度 | 4.676 | 31 | .151 |  |  |  |  |  |
|  | Greenhouse-Geisser | 4.676 | 31.000 | .151 |  |  |  |  |  |
|  | Huynh-Feldt | 4.676 | 31.000 | .151 |  |  |  |  |  |
|  | 下限 | 4.676 | 31.000 | .151 |  |  |  |  |  |
| a. 使用 alpha 的计算结果 = .05 | | | | | | | | | |

改后：第一步 （用公式算效应量）

固定从句类型：(不区分抽象度)

记者看到一个穿着黑衣的小偷逃离了现场。90 4.78

记者看到一片献给孩子的爱心送到了学校。67 3.84

VS.

记者看到一个子弹击中的小偷逃离了现场。65 3.71

记者看到一片社会奉献的爱心送到了学校。60 3.62

| **组统计量** | | | | | |
| --- | --- | --- | --- | --- | --- |
|  | 从句类型 | N | 均值 | 标准差 | 均值的标准误 |
| VAR00003 | 1.00 | 70 | 4.3071 | .59601 | .07124 |
|  | 2.00 | 70 | 3.6161 | .42669 | .05100 |

| **独立样本检验** | | | | | | | | | | |
| --- | --- | --- | --- | --- | --- | --- | --- | --- | --- | --- |
|  | | 方差方程的 Levene 检验 | | 均值方程的 t 检验 | | | | | | |
|  |  | F | Sig. | t | df | Sig.(双侧) | 均值差值 | 标准误差值 | 差分的 95% 置信区间 | |
|  |  |  |  |  |  |  |  |  | 下限 | 上限 |
| VAR00003 | 假设方差相等 | 11.017 | .001 | 7.887 | 138 | .000 | .69100 | .08761 | .51777 | .86423 |
|  | 假设方差不相等 |  |  | 7.887 | 125.015 | .000 | .69100 | .08761 | .51761 | .86439 |

记者看到穿着黑衣的一个小偷逃离了现场。80 4.205

记者看到献给孩子的一片爱心送到了学校。89 4.52

VS.

记者看到子弹击中的一个小偷逃离了现场。75 4.15

记者看到社会奉献的一片爱心送到了学校。73 3.96

| **组统计量** | | | | | |
| --- | --- | --- | --- | --- | --- |
|  | 从句类型 | N | 均值 | 标准差 | 均值的标准误 |
| VAR00004 | 1.00 | 70 | 4.4154 | .40957 | .04895 |
|  | 2.00 | 70 | 4.0571 | .60118 | .07185 |

| **独立样本检验** | | | | | | | | | | |
| --- | --- | --- | --- | --- | --- | --- | --- | --- | --- | --- |
|  | | 方差方程的 Levene 检验 | | 均值方程的 t 检验 | | | | | | |
|  |  | F | Sig. | t | df | Sig.(双侧) | 均值差值 | 标准误差值 | 差分的 95% 置信区间 | |
|  |  |  |  |  |  |  |  |  | 下限 | 上限 |
| VAR00004 | 假设方差相等 | 16.910 | .000 | 4.121 | 138 | .000 | .35829 | .08694 | .18637 | .53020 |
|  | 假设方差不相等 |  |  | 4.121 | 121.699 | .000 | .35829 | .08694 | .18617 | .53041 |

第二步：

**固定从句类型：**

**抽象度：**

1 A1B1C1记者看到一个穿着黑衣的小偷逃离了现场。90 4.78

A1B2C1记者看到一片献给孩子的爱心送到了学校。67 3.84

| **组统计量** | | | | | |
| --- | --- | --- | --- | --- | --- |
|  | 主从测抽象度 | N | 均值 | 标准差 | 均值的标准误 |
| VAR00003 | 1.00 | 35 | 4.7780 | .15541 | .02627 |
|  | 2.00 | 35 | 3.8363 | .49020 | .08286 |

| **独立样本检验** | | | | | | | | | | |
| --- | --- | --- | --- | --- | --- | --- | --- | --- | --- | --- |
|  | | 方差方程的 Levene 检验 | | 均值方程的 t 检验 | | | | | | |
|  |  | F | Sig. | t | df | Sig.(双侧) | 均值差值 | 标准误差值 | 差分的 95% 置信区间 | |
|  |  |  |  |  |  |  |  |  | 下限 | 上限 |
| VAR00003 | 假设方差相等 | 22.500 | .000 | 10.834 | 68 | .000 | .94171 | .08692 | .76826 | 1.11517 |
|  | 假设方差不相等 |  |  | 10.834 | 40.766 | .000 | .94171 | .08692 | .76614 | 1.11729 |

2 A2B1C1记者看到穿着黑衣的一个小偷逃离了现场。80 4.31

A2B2C1记者看到献给孩子的一片爱心送到了学校。89 4.52

| **组统计量** | | | | | |
| --- | --- | --- | --- | --- | --- |
|  | 主从测抽象度 | N | 均值 | 标准差 | 均值的标准误 |
| VAR00004 | 1.00 | 35 | 4.3123 | .41383 | .06995 |
|  | 2.00 | 35 | 4.5186 | .38375 | .06487 |

| **独立样本检验** | | | | | | | | | | |
| --- | --- | --- | --- | --- | --- | --- | --- | --- | --- | --- |
|  | | 方差方程的 Levene 检验 | | 均值方程的 t 检验 | | | | | | |
|  |  | F | Sig. | t | df | Sig.(双侧) | 均值差值 | 标准误差值 | 差分的 95% 置信区间 | |
|  |  |  |  |  |  |  |  |  | 下限 | 上限 |
| VAR00004 | 假设方差相等 | 1.174 | .282 | -2.162 | 68 | .034 | -.20629 | .09540 | -.39665 | -.01592 |
|  | 假设方差不相等 |  |  | -2.162 | 67.616 | .034 | -.20629 | .09540 | -.39667 | -.01590 |

3 A2B1C2记者看到子弹击中的一个小偷逃离了现场。75 4.15

A2B2C2记者看到社会奉献的一片爱心送到了学校。73 3.96

| **组统计量** | | | | | |
| --- | --- | --- | --- | --- | --- |
|  | 宾从测抽象度 | N | 均值 | 标准差 | 均值的标准误 |
| VAR00005 | 1.00 | 35 | 4.1500 | .54057 | .09137 |
|  | 2.00 | 35 | 3.9643 | .65076 | .11000 |

| **独立样本检验** | | | | | | | | | | |
| --- | --- | --- | --- | --- | --- | --- | --- | --- | --- | --- |
|  | | 方差方程的 Levene 检验 | | 均值方程的 t 检验 | | | | | | |
|  |  | F | Sig. | t | df | Sig.(双侧) | 均值差值 | 标准误差值 | 差分的 95% 置信区间 | |
|  |  |  |  |  |  |  |  |  | 下限 | 上限 |
| VAR00005 | 假设方差相等 | .548 | .462 | 1.299 | 68 | .198 | .18571 | .14300 | -.09964 | .47106 |
|  | 假设方差不相等 |  |  | 1.299 | 65.787 | .199 | .18571 | .14300 | -.09981 | .47124 |

4 A2B1C2记者看到一个子弹击中的小偷逃离了现场。65 3.71（接受度无差异）

A1B2C2记者看到一片社会奉献的爱心送到了学校。60 3.62

| **组统计量** | | | | | |
| --- | --- | --- | --- | --- | --- |
|  | 宾从测抽象度 | N | 均值 | 标准差 | 均值的标准误 |
| VAR00006 | 1.00 | 35 | 3.7151 | .41780 | .07062 |
|  | 2.00 | 35 | 3.5171 | .48208 | .08149 |

| **独立样本检验** | | | | | | | | | | |
| --- | --- | --- | --- | --- | --- | --- | --- | --- | --- | --- |
|  | | 方差方程的 Levene 检验 | | 均值方程的 t 检验 | | | | | | |
|  |  | F | Sig. | t | df | Sig.(双侧) | 均值差值 | 标准误差值 | 差分的 95% 置信区间 | |
|  |  |  |  |  |  |  |  |  | 下限 | 上限 |
| VAR00006 | 假设方差相等 | .125 | .725 | 1.836 | 68 | .071 | .19800 | .10783 | -.01717 | .41317 |
|  | 假设方差不相等 |  |  | 1.836 | 66.654 | .071 | .19800 | .10783 | -.01725 | .41325 |

反应时：

8个条件的主效应和交互（被试内）

| **描述性统计量** | | | |
| --- | --- | --- | --- |
|  | 均值 | 标准 偏差 | N |
| A1B1C1 | 3390.4857 | 473.64086 | 35 |
| A1B1C2 | 3815.4857 | 588.94374 | 35 |
| A1B2C1 | 3735.9714 | 501.84709 | 35 |
| A1B2C2 | 3904.8857 | 553.68614 | 35 |
| A2B1C1 | 3494.4286 | 619.15337 | 35 |
| A2B1C2 | 3588.0571 | 567.83619 | 35 |
| A2B2C1 | 3561.4571 | 388.44398 | 35 |
| A2B2C2 | 3662.3714 | 650.68204 | 35 |

| **Mauchly 的球形度检验^a^** | | | | | | | |
| --- | --- | --- | --- | --- | --- | --- | --- |
| 度量: MEASURE_1 | | | | | | | |
| 主体内效应 | Mauchly 的 W | 近似卡方 | df | Sig. | Epsilon^b^ | | |
|  |  |  |  |  | Greenhouse-Geisser | Huynh-Feldt | 下限 |
| A | 1.000 | .000 | 0 | . | 1.000 | 1.000 | 1.000 |
| B | 1.000 | .000 | 0 | . | 1.000 | 1.000 | 1.000 |
| C | 1.000 | .000 | 0 | . | 1.000 | 1.000 | 1.000 |
| A * B | 1.000 | .000 | 0 | . | 1.000 | 1.000 | 1.000 |
| A * C | 1.000 | .000 | 0 | . | 1.000 | 1.000 | 1.000 |
| B * C | 1.000 | .000 | 0 | . | 1.000 | 1.000 | 1.000 |
| A * B * C | 1.000 | .000 | 0 | . | 1.000 | 1.000 | 1.000 |
| 检验零假设，即标准正交转换因变量的误差协方差矩阵与一个单位矩阵成比例。 | | | | | | | |
| a. 设计 : 截距  主体内设计: A + B + C + A * B + A * C + B * C + A * B * C | | | | | | | |
| b. 可用于调整显著性平均检验的自由度。 在"主体内效应检验"表格中显示修正后的检验。 | | | | | | | |

| **主体内效应的检验** | | | | | | | | | |
| --- | --- | --- | --- | --- | --- | --- | --- | --- | --- |
| 度量: MEASURE_1 | | | | | | | | | |
| 源 | | III 型平方和 | df | 均方 | F | Sig. | 偏 Eta 方 | 非中心 参数 | 观测到的幂^a^ |
| A | 采用的球形度 | 1278181.157 | 1 | 1278181.157 | 3.677 | .064 | .098 | 3.677 | .462 |
|  | Greenhouse-Geisser | 1278181.157 | 1.000 | 1278181.157 | 3.677 | .064 | .098 | 3.677 | .462 |
|  | Huynh-Feldt | 1278181.157 | 1.000 | 1278181.157 | 3.677 | .064 | .098 | 3.677 | .462 |
|  | 下限 | 1278181.157 | 1.000 | 1278181.157 | 3.677 | .064 | .098 | 3.677 | .462 |
| 误差 (A) | 采用的球形度 | 11820259.593 | 34 | 347654.694 |  |  |  |  |  |
|  | Greenhouse-Geisser | 11820259.593 | 34.000 | 347654.694 |  |  |  |  |  |
|  | Huynh-Feldt | 11820259.593 | 34.000 | 347654.694 |  |  |  |  |  |
|  | 下限 | 11820259.593 | 34.000 | 347654.694 |  |  |  |  |  |
| B | 采用的球形度 | 1452672.229 | 1 | 1452672.229 | 6.121 | .019 | .153 | 6.121 | .671 |
|  | Greenhouse-Geisser | 1452672.229 | 1.000 | 1452672.229 | 6.121 | .019 | .153 | 6.121 | .671 |
|  | Huynh-Feldt | 1452672.229 | 1.000 | 1452672.229 | 6.121 | .019 | .153 | 6.121 | .671 |
|  | 下限 | 1452672.229 | 1.000 | 1452672.229 | 6.121 | .019 | .153 | 6.121 | .671 |
| 误差 (B) | 采用的球形度 | 8069415.521 | 34 | 237335.751 |  |  |  |  |  |
|  | Greenhouse-Geisser | 8069415.521 | 34.000 | 237335.751 |  |  |  |  |  |
|  | Huynh-Feldt | 8069415.521 | 34.000 | 237335.751 |  |  |  |  |  |
|  | 下限 | 8069415.521 | 34.000 | 237335.751 |  |  |  |  |  |
| C | 采用的球形度 | 2719782.914 | 1 | 2719782.914 | 8.821 | .005 | .206 | 8.821 | .823 |
|  | Greenhouse-Geisser | 2719782.914 | 1.000 | 2719782.914 | 8.821 | .005 | .206 | 8.821 | .823 |
|  | Huynh-Feldt | 2719782.914 | 1.000 | 2719782.914 | 8.821 | .005 | .206 | 8.821 | .823 |
|  | 下限 | 2719782.914 | 1.000 | 2719782.914 | 8.821 | .005 | .206 | 8.821 | .823 |
| 误差 (C) | 采用的球形度 | 10483533.836 | 34 | 308339.230 |  |  |  |  |  |
|  | Greenhouse-Geisser | 10483533.836 | 34.000 | 308339.230 |  |  |  |  |  |
|  | Huynh-Feldt | 10483533.836 | 34.000 | 308339.230 |  |  |  |  |  |
|  | 下限 | 10483533.836 | 34.000 | 308339.230 |  |  |  |  |  |
| A * B | 采用的球形度 | 376982.414 | 1 | 376982.414 | 1.301 | .262 | .037 | 1.301 | .198 |
|  | Greenhouse-Geisser | 376982.414 | 1.000 | 376982.414 | 1.301 | .262 | .037 | 1.301 | .198 |
|  | Huynh-Feldt | 376982.414 | 1.000 | 376982.414 | 1.301 | .262 | .037 | 1.301 | .198 |
|  | 下限 | 376982.414 | 1.000 | 376982.414 | 1.301 | .262 | .037 | 1.301 | .198 |
| 误差 (A*B) | 采用的球形度 | 9851966.336 | 34 | 289763.716 |  |  |  |  |  |
|  | Greenhouse-Geisser | 9851966.336 | 34.000 | 289763.716 |  |  |  |  |  |
|  | Huynh-Feldt | 9851966.336 | 34.000 | 289763.716 |  |  |  |  |  |
|  | 下限 | 9851966.336 | 34.000 | 289763.716 |  |  |  |  |  |
| A * C | 采用的球形度 | 697801.729 | 1 | 697801.729 | 2.909 | .097 | .079 | 2.909 | .381 |
|  | Greenhouse-Geisser | 697801.729 | 1.000 | 697801.729 | 2.909 | .097 | .079 | 2.909 | .381 |
|  | Huynh-Feldt | 697801.729 | 1.000 | 697801.729 | 2.909 | .097 | .079 | 2.909 | .381 |
|  | 下限 | 697801.729 | 1.000 | 697801.729 | 2.909 | .097 | .079 | 2.909 | .381 |
| 误差 (A*C) | 采用的球形度 | 8154936.021 | 34 | 239851.059 |  |  |  |  |  |
|  | Greenhouse-Geisser | 8154936.021 | 34.000 | 239851.059 |  |  |  |  |  |
|  | Huynh-Feldt | 8154936.021 | 34.000 | 239851.059 |  |  |  |  |  |
|  | 下限 | 8154936.021 | 34.000 | 239851.059 |  |  |  |  |  |
| B * C | 采用的球形度 | 270818.800 | 1 | 270818.800 | .771 | .386 | .022 | .771 | .137 |
|  | Greenhouse-Geisser | 270818.800 | 1.000 | 270818.800 | .771 | .386 | .022 | .771 | .137 |
|  | Huynh-Feldt | 270818.800 | 1.000 | 270818.800 | .771 | .386 | .022 | .771 | .137 |
|  | 下限 | 270818.800 | 1.000 | 270818.800 | .771 | .386 | .022 | .771 | .137 |
| 误差 (B*C) | 采用的球形度 | 11945109.950 | 34 | 351326.763 |  |  |  |  |  |
|  | Greenhouse-Geisser | 11945109.950 | 34.000 | 351326.763 |  |  |  |  |  |
|  | Huynh-Feldt | 11945109.950 | 34.000 | 351326.763 |  |  |  |  |  |
|  | 下限 | 11945109.950 | 34.000 | 351326.763 |  |  |  |  |  |
| A * B * C | 采用的球形度 | 303469.729 | 1 | 303469.729 | .853 | .362 | .024 | .853 | .146 |
|  | Greenhouse-Geisser | 303469.729 | 1.000 | 303469.729 | .853 | .362 | .024 | .853 | .146 |
|  | Huynh-Feldt | 303469.729 | 1.000 | 303469.729 | .853 | .362 | .024 | .853 | .146 |
|  | 下限 | 303469.729 | 1.000 | 303469.729 | .853 | .362 | .024 | .853 | .146 |
| 误差 (A*B*C) | 采用的球形度 | 12091553.021 | 34 | 355633.912 |  |  |  |  |  |
|  | Greenhouse-Geisser | 12091553.021 | 34.000 | 355633.912 |  |  |  |  |  |
|  | Huynh-Feldt | 12091553.021 | 34.000 | 355633.912 |  |  |  |  |  |
|  | 下限 | 12091553.021 | 34.000 | 355633.912 |  |  |  |  |  |
| a. 使用 alpha 的计算结果 = .05 | | | | | | | | | |

抽象度和从句类型有主效应 关内、关外无主效应

项目内：

| **Mauchly 的球形度检验^a^** | | | | | | | |
| --- | --- | --- | --- | --- | --- | --- | --- |
| 度量: MEASURE_1 | | | | | | | |
| 主体内效应 | Mauchly 的 W | 近似卡方 | df | Sig. | Epsilon^b^ | | |
|  |  |  |  |  | Greenhouse-Geisser | Huynh-Feldt | 下限 |
| A | 1.000 | .000 | 0 | . | 1.000 | 1.000 | 1.000 |
| B | 1.000 | .000 | 0 | . | 1.000 | 1.000 | 1.000 |
| C | 1.000 | .000 | 0 | . | 1.000 | 1.000 | 1.000 |
| A * B | 1.000 | .000 | 0 | . | 1.000 | 1.000 | 1.000 |
| A * C | 1.000 | .000 | 0 | . | 1.000 | 1.000 | 1.000 |
| B * C | 1.000 | .000 | 0 | . | 1.000 | 1.000 | 1.000 |
| A * B * C | 1.000 | .000 | 0 | . | 1.000 | 1.000 | 1.000 |
| 检验零假设，即标准正交转换因变量的误差协方差矩阵与一个单位矩阵成比例。 | | | | | | | |
| a. 设计 : 截距  主体内设计: A + B + C + A * B + A * C + B * C + A * B * C | | | | | | | |
| b. 可用于调整显著性平均检验的自由度。 在"主体内效应检验"表格中显示修正后的检验。 | | | | | | | |

| **主体内效应的检验** | | | | | | | | | |
| --- | --- | --- | --- | --- | --- | --- | --- | --- | --- |
| 度量: MEASURE_1 | | | | | | | | | |
| 源 | | III 型平方和 | df | 均方 | F | Sig. | 偏 Eta 方 | 非中心 参数 | 观测到的幂^a^ |
| A | 采用的球形度 | 1213026.891 | 1 | 1213026.891 | 3.898 | .057 | .112 | 3.898 | .481 |
|  | Greenhouse-Geisser | 1213026.891 | 1.000 | 1213026.891 | 3.898 | .057 | .112 | 3.898 | .481 |
|  | Huynh-Feldt | 1213026.891 | 1.000 | 1213026.891 | 3.898 | .057 | .112 | 3.898 | .481 |
|  | 下限 | 1213026.891 | 1.000 | 1213026.891 | 3.898 | .057 | .112 | 3.898 | .481 |
| 误差 (A) | 采用的球形度 | 9645891.359 | 31 | 311157.786 |  |  |  |  |  |
|  | Greenhouse-Geisser | 9645891.359 | 31.000 | 311157.786 |  |  |  |  |  |
|  | Huynh-Feldt | 9645891.359 | 31.000 | 311157.786 |  |  |  |  |  |
|  | 下限 | 9645891.359 | 31.000 | 311157.786 |  |  |  |  |  |
| B | 采用的球形度 | 1362472.563 | 1 | 1362472.563 | 4.823 | .036 | .135 | 4.823 | .567 |
|  | Greenhouse-Geisser | 1362472.563 | 1.000 | 1362472.563 | 4.823 | .036 | .135 | 4.823 | .567 |
|  | Huynh-Feldt | 1362472.563 | 1.000 | 1362472.563 | 4.823 | .036 | .135 | 4.823 | .567 |
|  | 下限 | 1362472.563 | 1.000 | 1362472.563 | 4.823 | .036 | .135 | 4.823 | .567 |
| 误差 (B) | 采用的球形度 | 8757327.188 | 31 | 282494.425 |  |  |  |  |  |
|  | Greenhouse-Geisser | 8757327.188 | 31.000 | 282494.425 |  |  |  |  |  |
|  | Huynh-Feldt | 8757327.188 | 31.000 | 282494.425 |  |  |  |  |  |
|  | 下限 | 8757327.188 | 31.000 | 282494.425 |  |  |  |  |  |
| C | 采用的球形度 | 2389729.516 | 1 | 2389729.516 | 9.739 | .004 | .239 | 9.739 | .856 |
|  | Greenhouse-Geisser | 2389729.516 | 1.000 | 2389729.516 | 9.739 | .004 | .239 | 9.739 | .856 |
|  | Huynh-Feldt | 2389729.516 | 1.000 | 2389729.516 | 9.739 | .004 | .239 | 9.739 | .856 |
|  | 下限 | 2389729.516 | 1.000 | 2389729.516 | 9.739 | .004 | .239 | 9.739 | .856 |
| 误差 (C) | 采用的球形度 | 7606410.234 | 31 | 245368.072 |  |  |  |  |  |
|  | Greenhouse-Geisser | 7606410.234 | 31.000 | 245368.072 |  |  |  |  |  |
|  | Huynh-Feldt | 7606410.234 | 31.000 | 245368.072 |  |  |  |  |  |
|  | 下限 | 7606410.234 | 31.000 | 245368.072 |  |  |  |  |  |
| A * B | 采用的球形度 | 331344.141 | 1 | 331344.141 | .846 | .365 | .027 | .846 | .145 |
|  | Greenhouse-Geisser | 331344.141 | 1.000 | 331344.141 | .846 | .365 | .027 | .846 | .145 |
|  | Huynh-Feldt | 331344.141 | 1.000 | 331344.141 | .846 | .365 | .027 | .846 | .145 |
|  | 下限 | 331344.141 | 1.000 | 331344.141 | .846 | .365 | .027 | .846 | .145 |
| 误差 (A*B) | 采用的球形度 | 12140444.609 | 31 | 391627.245 |  |  |  |  |  |
|  | Greenhouse-Geisser | 12140444.609 | 31.000 | 391627.245 |  |  |  |  |  |
|  | Huynh-Feldt | 12140444.609 | 31.000 | 391627.245 |  |  |  |  |  |
|  | 下限 | 12140444.609 | 31.000 | 391627.245 |  |  |  |  |  |
| A * C | 采用的球形度 | 544644.000 | 1 | 544644.000 | 1.334 | .257 | .041 | 1.334 | .201 |
|  | Greenhouse-Geisser | 544644.000 | 1.000 | 544644.000 | 1.334 | .257 | .041 | 1.334 | .201 |
|  | Huynh-Feldt | 544644.000 | 1.000 | 544644.000 | 1.334 | .257 | .041 | 1.334 | .201 |
|  | 下限 | 544644.000 | 1.000 | 544644.000 | 1.334 | .257 | .041 | 1.334 | .201 |
| 误差 (A*C) | 采用的球形度 | 12653567.750 | 31 | 408179.605 |  |  |  |  |  |
|  | Greenhouse-Geisser | 12653567.750 | 31.000 | 408179.605 |  |  |  |  |  |
|  | Huynh-Feldt | 12653567.750 | 31.000 | 408179.605 |  |  |  |  |  |
|  | 下限 | 12653567.750 | 31.000 | 408179.605 |  |  |  |  |  |
| B * C | 采用的球形度 | 280767.516 | 1 | 280767.516 | 1.996 | .168 | .060 | 1.996 | .278 |
|  | Greenhouse-Geisser | 280767.516 | 1.000 | 280767.516 | 1.996 | .168 | .060 | 1.996 | .278 |
|  | Huynh-Feldt | 280767.516 | 1.000 | 280767.516 | 1.996 | .168 | .060 | 1.996 | .278 |
|  | 下限 | 280767.516 | 1.000 | 280767.516 | 1.996 | .168 | .060 | 1.996 | .278 |
| 误差 (B*C) | 采用的球形度 | 4360939.734 | 31 | 140675.475 |  |  |  |  |  |
|  | Greenhouse-Geisser | 4360939.734 | 31.000 | 140675.475 |  |  |  |  |  |
|  | Huynh-Feldt | 4360939.734 | 31.000 | 140675.475 |  |  |  |  |  |
|  | 下限 | 4360939.734 | 31.000 | 140675.475 |  |  |  |  |  |
| A * B * C | 采用的球形度 | 211830.062 | 1 | 211830.062 | 1.089 | .305 | .034 | 1.089 | .173 |
|  | Greenhouse-Geisser | 211830.062 | 1.000 | 211830.062 | 1.089 | .305 | .034 | 1.089 | .173 |
|  | Huynh-Feldt | 211830.062 | 1.000 | 211830.062 | 1.089 | .305 | .034 | 1.089 | .173 |
|  | 下限 | 211830.062 | 1.000 | 211830.062 | 1.089 | .305 | .034 | 1.089 | .173 |
| 误差 (A*B*C) | 采用的球形度 | 6028838.188 | 31 | 194478.651 |  |  |  |  |  |
|  | Greenhouse-Geisser | 6028838.188 | 31.000 | 194478.651 |  |  |  |  |  |
|  | Huynh-Feldt | 6028838.188 | 31.000 | 194478.651 |  |  |  |  |  |
|  | 下限 | 6028838.188 | 31.000 | 194478.651 |  |  |  |  |  |
| a. 使用 alpha 的计算结果 = .05 | | | | | | | | | |

同上！！！

改后：第一步

固定从句类型：(不区分抽象度)

记者看到一个穿着黑衣的小偷逃离了现场。90 4.78

记者看到一片献给孩子的爱心送到了学校。67 3.84

VS.

记者看到一个子弹击中的小偷逃离了现场。65 3.71

记者看到一片社会奉献的爱心送到了学校。60 3.62

| **组统计量** | | | | | |
| --- | --- | --- | --- | --- | --- |
|  | 从句类型反应时 | N | 均值 | 标准差 | 均值的标准误 |
| VAR00004 | 1.00 | 70 | 3563.2286 | 514.69896 | 61.51829 |
|  | 2.00 | 70 | 3860.1857 | 569.21317 | 68.03399 |

| **独立样本检验** | | | | | | | | | | |
| --- | --- | --- | --- | --- | --- | --- | --- | --- | --- | --- |
|  | | 方差方程的 Levene 检验 | | 均值方程的 t 检验 | | | | | | |
|  |  | F | Sig. | t | df | Sig.(双侧) | 均值差值 | 标准误差值 | 差分的 95% 置信区间 | |
|  |  |  |  |  |  |  |  |  | 下限 | 上限 |
| VAR00004 | 假设方差相等 | .685 | .409 | -3.238 | 138 | .002 | -296.95714 | 91.72308 | -478.32152 | -115.59277 |
|  | 假设方差不相等 |  |  | -3.238 | 136.625 | .002 | -296.95714 | 91.72308 | -478.33767 | -115.57662 |

记者看到穿着黑衣的一个小偷逃离了现场。80 4.205

记者看到献给孩子的一片爱心送到了学校。89 4.52

VS.

记者看到子弹击中的一个小偷逃离了现场。75 4.15

记者看到社会奉献的一片爱心送到了学校。73 3.96

| **组统计量** | | | | | |
| --- | --- | --- | --- | --- | --- |
|  | 从句类型反应时 | N | 均值 | 标准差 | 均值的标准误 |
| VAR00003 | 1.00 | 70 | 3447.9429 | 555.61601 | 66.40882 |
|  | 2.00 | 70 | 3668.0714 | 636.12095 | 76.03100 |

| **独立样本检验** | | | | | | | | | | |
| --- | --- | --- | --- | --- | --- | --- | --- | --- | --- | --- |
|  | | 方差方程的 Levene 检验 | | 均值方程的 t 检验 | | | | | | |
|  |  | F | Sig. | t | df | Sig.(双侧) | 均值差值 | 标准误差值 | 差分的 95% 置信区间 | |
|  |  |  |  |  |  |  |  |  | 下限 | 上限 |
| VAR00003 | 假设方差相等 | 2.084 | .151 | -2.181 | 138 | .031 | -220.12857 | 100.94971 | -419.73678 | -20.52036 |
|  | 假设方差不相等 |  |  | -2.181 | 135.548 | .031 | -220.12857 | 100.94971 | -419.76872 | -20.48842 |

结论：量词位置置内、外不会影响句子接受度

第二步 加入语义因素

**固定从句类型：**

**抽象度：**

1 A1B1C1记者看到一个穿着黑衣的小偷逃离了现场。90 4.78

A1B2C1记者看到一片献给孩子的爱心送到了学校。67 3.84

| **组统计量** | | | | | |
| --- | --- | --- | --- | --- | --- |
|  | 抽象度分组 | N | 均值 | 标准差 | 均值的标准误 |
| VAR00002 | 1.00 | 35 | 3390.4857 | 473.64086 | 80.05992 |
|  | 2.00 | 35 | 3735.9714 | 501.84709 | 84.82764 |

| **独立样本检验** | | | | | | | | | | |
| --- | --- | --- | --- | --- | --- | --- | --- | --- | --- | --- |
|  | | 方差方程的 Levene 检验 | | 均值方程的 t 检验 | | | | | | |
|  |  | F | Sig. | t | df | Sig.(双侧) | 均值差值 | 标准误差值 | 差分的 95% 置信区间 | |
|  |  |  |  |  |  |  |  |  | 下限 | 上限 |
| VAR00002 | 假设方差相等 | .111 | .740 | -2.962 | 68 | .004 | -345.48571 | 116.64184 | -578.24088 | -112.73054 |
|  | 假设方差不相等 |  |  | -2.962 | 67.774 | .004 | -345.48571 | 116.64184 | -578.25496 | -112.71647 |

vs

2 A2B1C1记者看到穿着黑衣的一个小偷逃离了现场。80 4.31

A2B2C1记者看到献给孩子的一片爱心送到了学校。89 4.52

| **组统计量** | | | | | |
| --- | --- | --- | --- | --- | --- |
|  | 抽象度分组 | N | 均值 | 标准差 | 均值的标准误 |
| VAR00003 | 1.00 | 35 | 3494.4286 | 619.15337 | 104.65602 |
|  | 2.00 | 35 | 3561.4571 | 388.44398 | 65.65902 |

| **独立样本检验** | | | | | | | | | | |
| --- | --- | --- | --- | --- | --- | --- | --- | --- | --- | --- |
|  | | 方差方程的 Levene 检验 | | 均值方程的 t 检验 | | | | | | |
|  |  | F | Sig. | t | df | Sig.(双侧) | 均值差值 | 标准误差值 | 差分的 95% 置信区间 | |
|  |  |  |  |  |  |  |  |  | 下限 | 上限 |
| VAR00003 | 假设方差相等 | 3.669 | .060 | -.543 | 68 | .589 | -67.02857 | 123.54752 | -313.56381 | 179.50666 |
|  | 假设方差不相等 |  |  | -.543 | 57.175 | .590 | -67.02857 | 123.54752 | -314.41182 | 180.35468 |

3 A2B1C2记者看到子弹击中的一个小偷逃离了现场。75 4.15

A2B2C2记者看到社会奉献的一片爱心送到了学校。73 3.96

| **组统计量** | | | | | |
| --- | --- | --- | --- | --- | --- |
|  | 抽象度分组 | N | 均值 | 标准差 | 均值的标准误 |
| VAR00004 | 1.00 | 35 | 3588.0571 | 567.83619 | 95.98183 |
|  | 2.00 | 35 | 3662.3714 | 650.68204 | 109.98534 |

| **独立样本检验** | | | | | | | | | | |
| --- | --- | --- | --- | --- | --- | --- | --- | --- | --- | --- |
|  | | 方差方程的 Levene 检验 | | 均值方程的 t 检验 | | | | | | |
|  |  | F | Sig. | t | df | Sig.(双侧) | 均值差值 | 标准误差值 | 差分的 95% 置信区间 | |
|  |  |  |  |  |  |  |  |  | 下限 | 上限 |
| VAR00004 | 假设方差相等 | .235 | .629 | -.509 | 68 | .612 | -74.31429 | 145.97701 | -365.60687 | 216.97830 |
|  | 假设方差不相等 |  |  | -.509 | 66.777 | .612 | -74.31429 | 145.97701 | -365.70353 | 217.07496 |

4 A1B1C2记者看到一个子弹击中的小偷逃离了现场。65 3.71（接受度无差异）

A1B2C2记者看到一片社会奉献的爱心送到了学校。60 3.62

| **组统计量** | | | | | |
| --- | --- | --- | --- | --- | --- |
|  | 抽象度分组 | N | 均值 | 标准差 | 均值的标准误 |
| VAR00005 | 1.00 | 35 | 3815.4857 | 588.94374 | 99.54966 |
|  | 2.00 | 35 | 3904.8857 | 553.68614 | 93.59004 |

| **独立样本检验** | | | | | | | | | | |
| --- | --- | --- | --- | --- | --- | --- | --- | --- | --- | --- |
|  | | 方差方程的 Levene 检验 | | 均值方程的 t 检验 | | | | | | |
|  |  | F | Sig. | t | df | Sig.(双侧) | 均值差值 | 标准误差值 | 差分的 95% 置信区间 | |
|  |  |  |  |  |  |  |  |  | 下限 | 上限 |
| VAR00005 | 假设方差相等 | .282 | .597 | -.654 | 68 | .515 | -89.40000 | 136.63539 | -362.05168 | 183.25168 |
|  | 假设方差不相等 |  |  | -.654 | 67.742 | .515 | -89.40000 | 136.63539 | -362.07045 | 183.27045 |

说明：只有第一个条件差异最大 抽象度影响数量词的位置！！！

首次注视时间：

被试内主效应和交互：

| **描述性统计量** | | | |
| --- | --- | --- | --- |
|  | 均值 | 标准 偏差 | N |
| A1B1C1 | 206.0571 | 41.40968 | 35 |
| A1B1C2 | 225.3429 | 50.66728 | 35 |
| A1B2C1 | 232.0286 | 41.27276 | 35 |
| A1B2C2 | 239.0857 | 41.06693 | 35 |
| A2B1C1 | 242.5143 | 46.96831 | 35 |
| A2B1C2 | 236.5143 | 46.08392 | 35 |
| A2B2C1 | 212.3714 | 35.87403 | 35 |
| A2B2C2 | 248.7714 | 66.42248 | 35 |

| **Mauchly 的球形度检验^a^** | | | | | | | |
| --- | --- | --- | --- | --- | --- | --- | --- |
| 度量: MEASURE_1 | | | | | | | |
| 主体内效应 | Mauchly 的 W | 近似卡方 | df | Sig. | Epsilon^b^ | | |
|  |  |  |  |  | Greenhouse-Geisser | Huynh-Feldt | 下限 |
| A | 1.000 | .000 | 0 | . | 1.000 | 1.000 | 1.000 |
| B | 1.000 | .000 | 0 | . | 1.000 | 1.000 | 1.000 |
| C | 1.000 | .000 | 0 | . | 1.000 | 1.000 | 1.000 |
| A * B | 1.000 | .000 | 0 | . | 1.000 | 1.000 | 1.000 |
| A * C | 1.000 | .000 | 0 | . | 1.000 | 1.000 | 1.000 |
| B * C | 1.000 | .000 | 0 | . | 1.000 | 1.000 | 1.000 |
| A * B * C | 1.000 | .000 | 0 | . | 1.000 | 1.000 | 1.000 |
| 检验零假设，即标准正交转换因变量的误差协方差矩阵与一个单位矩阵成比例。 | | | | | | | |
| a. 设计 : 截距  主体内设计: A + B + C + A * B + A * C + B * C + A * B * C | | | | | | | |
| b. 可用于调整显著性平均检验的自由度。 在"主体内效应检验"表格中显示修正后的检验。 | | | | | | | |

| **主体内效应的检验** | | | | | | | | | |
| --- | --- | --- | --- | --- | --- | --- | --- | --- | --- |
| 度量: MEASURE_1 | | | | | | | | | |
| 源 | | III 型平方和 | df | 均方 | F | Sig. | 偏 Eta 方 | 非中心 参数 | 观测到的幂^a^ |
| A | 采用的球形度 | 6204.014 | 1 | 6204.014 | 3.154 | .085 | .085 | 3.154 | .408 |
|  | Greenhouse-Geisser | 6204.014 | 1.000 | 6204.014 | 3.154 | .085 | .085 | 3.154 | .408 |
|  | Huynh-Feldt | 6204.014 | 1.000 | 6204.014 | 3.154 | .085 | .085 | 3.154 | .408 |
|  | 下限 | 6204.014 | 1.000 | 6204.014 | 3.154 | .085 | .085 | 3.154 | .408 |
| 误差 (A) | 采用的球形度 | 66881.486 | 34 | 1967.103 |  |  |  |  |  |
|  | Greenhouse-Geisser | 66881.486 | 34.000 | 1967.103 |  |  |  |  |  |
|  | Huynh-Feldt | 66881.486 | 34.000 | 1967.103 |  |  |  |  |  |
|  | 下限 | 66881.486 | 34.000 | 1967.103 |  |  |  |  |  |
| B | 采用的球形度 | 2084.629 | 1 | 2084.629 | .880 | .355 | .025 | .880 | .149 |
|  | Greenhouse-Geisser | 2084.629 | 1.000 | 2084.629 | .880 | .355 | .025 | .880 | .149 |
|  | Huynh-Feldt | 2084.629 | 1.000 | 2084.629 | .880 | .355 | .025 | .880 | .149 |
|  | 下限 | 2084.629 | 1.000 | 2084.629 | .880 | .355 | .025 | .880 | .149 |
| 误差 (B) | 采用的球形度 | 80510.371 | 34 | 2367.952 |  |  |  |  |  |
|  | Greenhouse-Geisser | 80510.371 | 34.000 | 2367.952 |  |  |  |  |  |
|  | Huynh-Feldt | 80510.371 | 34.000 | 2367.952 |  |  |  |  |  |
|  | 下限 | 80510.371 | 34.000 | 2367.952 |  |  |  |  |  |
| C | 采用的球形度 | 14086.414 | 1 | 14086.414 | 10.644 | .003 | .238 | 10.644 | .887 |
|  | Greenhouse-Geisser | 14086.414 | 1.000 | 14086.414 | 10.644 | .003 | .238 | 10.644 | .887 |
|  | Huynh-Feldt | 14086.414 | 1.000 | 14086.414 | 10.644 | .003 | .238 | 10.644 | .887 |
|  | 下限 | 14086.414 | 1.000 | 14086.414 | 10.644 | .003 | .238 | 10.644 | .887 |
| 误差 (C) | 采用的球形度 | 44997.086 | 34 | 1323.444 |  |  |  |  |  |
|  | Greenhouse-Geisser | 44997.086 | 34.000 | 1323.444 |  |  |  |  |  |
|  | Huynh-Feldt | 44997.086 | 34.000 | 1323.444 |  |  |  |  |  |
|  | 下限 | 44997.086 | 34.000 | 1323.444 |  |  |  |  |  |
| A * B | 采用的球形度 | 14515.200 | 1 | 14515.200 | 5.966 | .020 | .149 | 5.966 | .660 |
|  | Greenhouse-Geisser | 14515.200 | 1.000 | 14515.200 | 5.966 | .020 | .149 | 5.966 | .660 |
|  | Huynh-Feldt | 14515.200 | 1.000 | 14515.200 | 5.966 | .020 | .149 | 5.966 | .660 |
|  | 下限 | 14515.200 | 1.000 | 14515.200 | 5.966 | .020 | .149 | 5.966 | .660 |
| 误差 (A*B) | 采用的球形度 | 82720.300 | 34 | 2432.950 |  |  |  |  |  |
|  | Greenhouse-Geisser | 82720.300 | 34.000 | 2432.950 |  |  |  |  |  |
|  | Huynh-Feldt | 82720.300 | 34.000 | 2432.950 |  |  |  |  |  |
|  | 下限 | 82720.300 | 34.000 | 2432.950 |  |  |  |  |  |
| A * C | 采用的球形度 | 72.014 | 1 | 72.014 | .023 | .880 | .001 | .023 | .053 |
|  | Greenhouse-Geisser | 72.014 | 1.000 | 72.014 | .023 | .880 | .001 | .023 | .053 |
|  | Huynh-Feldt | 72.014 | 1.000 | 72.014 | .023 | .880 | .001 | .023 | .053 |
|  | 下限 | 72.014 | 1.000 | 72.014 | .023 | .880 | .001 | .023 | .053 |
| 误差 (A*C) | 采用的球形度 | 105223.986 | 34 | 3094.823 |  |  |  |  |  |
|  | Greenhouse-Geisser | 105223.986 | 34.000 | 3094.823 |  |  |  |  |  |
|  | Huynh-Feldt | 105223.986 | 34.000 | 3094.823 |  |  |  |  |  |
|  | 下限 | 105223.986 | 34.000 | 3094.823 |  |  |  |  |  |
| B * C | 采用的球形度 | 3982.629 | 1 | 3982.629 | 1.467 | .234 | .041 | 1.467 | .218 |
|  | Greenhouse-Geisser | 3982.629 | 1.000 | 3982.629 | 1.467 | .234 | .041 | 1.467 | .218 |
|  | Huynh-Feldt | 3982.629 | 1.000 | 3982.629 | 1.467 | .234 | .041 | 1.467 | .218 |
|  | 下限 | 3982.629 | 1.000 | 3982.629 | 1.467 | .234 | .041 | 1.467 | .218 |
| 误差 (B*C) | 采用的球形度 | 92323.871 | 34 | 2715.408 |  |  |  |  |  |
|  | Greenhouse-Geisser | 92323.871 | 34.000 | 2715.408 |  |  |  |  |  |
|  | Huynh-Feldt | 92323.871 | 34.000 | 2715.408 |  |  |  |  |  |
|  | 下限 | 92323.871 | 34.000 | 2715.408 |  |  |  |  |  |
| A * B * C | 采用的球形度 | 13056.229 | 1 | 13056.229 | 5.530 | .025 | .140 | 5.530 | .627 |
|  | Greenhouse-Geisser | 13056.229 | 1.000 | 13056.229 | 5.530 | .025 | .140 | 5.530 | .627 |
|  | Huynh-Feldt | 13056.229 | 1.000 | 13056.229 | 5.530 | .025 | .140 | 5.530 | .627 |
|  | 下限 | 13056.229 | 1.000 | 13056.229 | 5.530 | .025 | .140 | 5.530 | .627 |
| 误差 (A*B*C) | 采用的球形度 | 80270.771 | 34 | 2360.905 |  |  |  |  |  |
|  | Greenhouse-Geisser | 80270.771 | 34.000 | 2360.905 |  |  |  |  |  |
|  | Huynh-Feldt | 80270.771 | 34.000 | 2360.905 |  |  |  |  |  |
|  | 下限 | 80270.771 | 34.000 | 2360.905 |  |  |  |  |  |
| a. 使用 alpha 的计算结果 = .05 | | | | | | | | | |

项目内主效应和交互：

| **主体内效应的检验** | | | | | | | | | |
| --- | --- | --- | --- | --- | --- | --- | --- | --- | --- |
| 度量: MEASURE_1 | | | | | | | | | |
| 源 | | III 型平方和 | df | 均方 | F | Sig. | 偏 Eta 方 | 非中心 参数 | 观测到的幂^a^ |
| A | 采用的球形度 | 5420.641 | 1 | 5420.641 | 3.865 | .058 | .111 | 3.865 | .478 |
|  | Greenhouse-Geisser | 5420.641 | 1.000 | 5420.641 | 3.865 | .058 | .111 | 3.865 | .478 |
|  | Huynh-Feldt | 5420.641 | 1.000 | 5420.641 | 3.865 | .058 | .111 | 3.865 | .478 |
|  | 下限 | 5420.641 | 1.000 | 5420.641 | 3.865 | .058 | .111 | 3.865 | .478 |
| 误差 (A) | 采用的球形度 | 43471.859 | 31 | 1402.318 |  |  |  |  |  |
|  | Greenhouse-Geisser | 43471.859 | 31.000 | 1402.318 |  |  |  |  |  |
|  | Huynh-Feldt | 43471.859 | 31.000 | 1402.318 |  |  |  |  |  |
|  | 下限 | 43471.859 | 31.000 | 1402.318 |  |  |  |  |  |
| B | 采用的球形度 | 213.891 | 1 | 213.891 | .174 | .679 | .006 | .174 | .069 |
|  | Greenhouse-Geisser | 213.891 | 1.000 | 213.891 | .174 | .679 | .006 | .174 | .069 |
|  | Huynh-Feldt | 213.891 | 1.000 | 213.891 | .174 | .679 | .006 | .174 | .069 |
|  | 下限 | 213.891 | 1.000 | 213.891 | .174 | .679 | .006 | .174 | .069 |
| 误差 (B) | 采用的球形度 | 38061.109 | 31 | 1227.778 |  |  |  |  |  |
|  | Greenhouse-Geisser | 38061.109 | 31.000 | 1227.778 |  |  |  |  |  |
|  | Huynh-Feldt | 38061.109 | 31.000 | 1227.778 |  |  |  |  |  |
|  | 下限 | 38061.109 | 31.000 | 1227.778 |  |  |  |  |  |
| C | 采用的球形度 | 9096.391 | 1 | 9096.391 | 6.836 | .014 | .181 | 6.836 | .717 |
|  | Greenhouse-Geisser | 9096.391 | 1.000 | 9096.391 | 6.836 | .014 | .181 | 6.836 | .717 |
|  | Huynh-Feldt | 9096.391 | 1.000 | 9096.391 | 6.836 | .014 | .181 | 6.836 | .717 |
|  | 下限 | 9096.391 | 1.000 | 9096.391 | 6.836 | .014 | .181 | 6.836 | .717 |
| 误差 (C) | 采用的球形度 | 41253.109 | 31 | 1330.745 |  |  |  |  |  |
|  | Greenhouse-Geisser | 41253.109 | 31.000 | 1330.745 |  |  |  |  |  |
|  | Huynh-Feldt | 41253.109 | 31.000 | 1330.745 |  |  |  |  |  |
|  | 下限 | 41253.109 | 31.000 | 1330.745 |  |  |  |  |  |
| A * B | 采用的球形度 | 5130.141 | 1 | 5130.141 | 3.057 | .090 | .090 | 3.057 | .395 |
|  | Greenhouse-Geisser | 5130.141 | 1.000 | 5130.141 | 3.057 | .090 | .090 | 3.057 | .395 |
|  | Huynh-Feldt | 5130.141 | 1.000 | 5130.141 | 3.057 | .090 | .090 | 3.057 | .395 |
|  | 下限 | 5130.141 | 1.000 | 5130.141 | 3.057 | .090 | .090 | 3.057 | .395 |
| 误差 (A*B) | 采用的球形度 | 52019.359 | 31 | 1678.044 |  |  |  |  |  |
|  | Greenhouse-Geisser | 52019.359 | 31.000 | 1678.044 |  |  |  |  |  |
|  | Huynh-Feldt | 52019.359 | 31.000 | 1678.044 |  |  |  |  |  |
|  | 下限 | 52019.359 | 31.000 | 1678.044 |  |  |  |  |  |
| A * C | 采用的球形度 | 165.766 | 1 | 165.766 | .088 | .769 | .003 | .088 | .059 |
|  | Greenhouse-Geisser | 165.766 | 1.000 | 165.766 | .088 | .769 | .003 | .088 | .059 |
|  | Huynh-Feldt | 165.766 | 1.000 | 165.766 | .088 | .769 | .003 | .088 | .059 |
|  | 下限 | 165.766 | 1.000 | 165.766 | .088 | .769 | .003 | .088 | .059 |
| 误差 (A*C) | 采用的球形度 | 58587.234 | 31 | 1889.911 |  |  |  |  |  |
|  | Greenhouse-Geisser | 58587.234 | 31.000 | 1889.911 |  |  |  |  |  |
|  | Huynh-Feldt | 58587.234 | 31.000 | 1889.911 |  |  |  |  |  |
|  | 下限 | 58587.234 | 31.000 | 1889.911 |  |  |  |  |  |
| B * C | 采用的球形度 | 922.641 | 1 | 922.641 | .605 | .443 | .019 | .605 | .117 |
|  | Greenhouse-Geisser | 922.641 | 1.000 | 922.641 | .605 | .443 | .019 | .605 | .117 |
|  | Huynh-Feldt | 922.641 | 1.000 | 922.641 | .605 | .443 | .019 | .605 | .117 |
|  | 下限 | 922.641 | 1.000 | 922.641 | .605 | .443 | .019 | .605 | .117 |
| 误差 (B*C) | 采用的球形度 | 47301.859 | 31 | 1525.866 |  |  |  |  |  |
|  | Greenhouse-Geisser | 47301.859 | 31.000 | 1525.866 |  |  |  |  |  |
|  | Huynh-Feldt | 47301.859 | 31.000 | 1525.866 |  |  |  |  |  |
|  | 下限 | 47301.859 | 31.000 | 1525.866 |  |  |  |  |  |
| A * B * C | 采用的球形度 | 9579.516 | 1 | 9579.516 | 4.867 | .035 | .136 | 4.867 | .570 |
|  | Greenhouse-Geisser | 9579.516 | 1.000 | 9579.516 | 4.867 | .035 | .136 | 4.867 | .570 |
|  | Huynh-Feldt | 9579.516 | 1.000 | 9579.516 | 4.867 | .035 | .136 | 4.867 | .570 |
|  | 下限 | 9579.516 | 1.000 | 9579.516 | 4.867 | .035 | .136 | 4.867 | .570 |
| 误差 (A*B*C) | 采用的球形度 | 61016.484 | 31 | 1968.274 |  |  |  |  |  |
|  | Greenhouse-Geisser | 61016.484 | 31.000 | 1968.274 |  |  |  |  |  |
|  | Huynh-Feldt | 61016.484 | 31.000 | 1968.274 |  |  |  |  |  |
|  | 下限 | 61016.484 | 31.000 | 1968.274 |  |  |  |  |  |
| a. 使用 alpha 的计算结果 = .05 | | | | | | | | | |

都有交互 说明在加工早期句法和语义均得以激活，并存在交互作用！

区分主宾从---句法

记者看到一个穿着黑衣的小偷逃离了现场。90 4.78

记者看到一片献给孩子的爱心送到了学校。67 3.84

VS.

记者看到一个子弹击中的小偷逃离了现场。65 3.71

记者看到一片社会奉献的爱心送到了学校。60 3.62

| **组统计量** | | | | | |
| --- | --- | --- | --- | --- | --- |
|  | 从句类型首次注视 | N | 均值 | 标准差 | 均值的标准误 |
| VAR00008 | 1.00 | 70 | 219.0429 | 43.07440 | 5.14838 |
|  | 2.00 | 70 | 232.2143 | 46.30238 | 5.53419 |

| **独立样本检验** | | | | | | | | | | |
| --- | --- | --- | --- | --- | --- | --- | --- | --- | --- | --- |
|  | | 方差方程的 Levene 检验 | | 均值方程的 t 检验 | | | | | | |
|  |  | F | Sig. | t | df | Sig.(双侧) | 均值差值 | 标准误差值 | 差分的 95% 置信区间 | |
|  |  |  |  |  |  |  |  |  | 下限 | 上限 |
| VAR00008 | 假设方差相等 | .309 | .579 | -1.743 | 138 | .084 | -13.17143 | 7.55864 | -28.11716 | 1.77430 |
|  | 假设方差不相等 |  |  | -1.743 | 137.286 | .084 | -13.17143 | 7.55864 | -28.11784 | 1.77499 |

记者看到穿着黑衣的一个小偷逃离了现场。80 4.205

记者看到献给孩子的一片爱心送到了学校。89 4.52

VS.

记者看到子弹击中的一个小偷逃离了现场。75 4.15

记者看到社会奉献的一片爱心送到了学校。73 3.96

| **组统计量** | | | | | |
| --- | --- | --- | --- | --- | --- |
|  | 从句类型首次注视 | N | 均值 | 标准差 | 均值的标准误 |
| VAR00007 | 1.00 | 70 | 227.4429 | 44.17706 | 5.28017 |
|  | 2.00 | 70 | 242.6429 | 57.08398 | 6.82284 |

| **独立样本检验** | | | | | | | | | | |
| --- | --- | --- | --- | --- | --- | --- | --- | --- | --- | --- |
|  | | 方差方程的 Levene 检验 | | 均值方程的 t 检验 | | | | | | |
|  |  | F | Sig. | t | df | Sig.(双侧) | 均值差值 | 标准误差值 | 差分的 95% 置信区间 | |
|  |  |  |  |  |  |  |  |  | 下限 | 上限 |
| VAR00007 | 假设方差相等 | 1.398 | .239 | -1.762 | 138 | .080 | -15.20000 | 8.62736 | -32.25891 | 1.85891 |
|  | 假设方差不相等 |  |  | -1.762 | 129.830 | .080 | -15.20000 | 8.62736 | -32.26841 | 1.86841 |

区分抽象度---语义

1 A1B1C1记者看到一个穿着黑衣的小偷逃离了现场。

A1B2C1记者看到一片献给孩子的爱心送到了学校。

| **组统计量** | | | | | |
| --- | --- | --- | --- | --- | --- |
|  | 首次注视抽象度分组 | N | 均值 | 标准差 | 均值的标准误 |
| VAR00006 | 1.00 | 35 | 206.0571 | 41.40968 | 6.99951 |
|  | 2.00 | 35 | 232.0286 | 41.27276 | 6.97637 |

| **独立样本检验** | | | | | | | | | | |
| --- | --- | --- | --- | --- | --- | --- | --- | --- | --- | --- |
|  | | 方差方程的 Levene 检验 | | 均值方程的 t 检验 | | | | | | |
|  |  | F | Sig. | t | df | Sig.(双侧) | 均值差值 | 标准误差值 | 差分的 95% 置信区间 | |
|  |  |  |  |  |  |  |  |  | 下限 | 上限 |
| VAR00006 | 假设方差相等 | .016 | .901 | -2.628 | 68 | .011 | -25.97143 | 9.88245 | -45.69156 | -6.25130 |
|  | 假设方差不相等 |  |  | -2.628 | 67.999 | .011 | -25.97143 | 9.88245 | -45.69156 | -6.25129 |

A2B1C1记者看到穿着黑衣的一个小偷逃离了现场。

A2B2C1记者看到献给孩子的一片爱心送到了学校。

| **组统计量** | | | | | |
| --- | --- | --- | --- | --- | --- |
|  | 首次注视抽象度分组 | N | 均值 | 标准差 | 均值的标准误 |
| VAR00007 | 1.00 | 35 | 242.5143 | 46.96831 | 7.93909 |
|  | 2.00 | 35 | 212.3714 | 35.87403 | 6.06382 |

| **独立样本检验** | | | | | | | | | | |
| --- | --- | --- | --- | --- | --- | --- | --- | --- | --- | --- |
|  | | 方差方程的 Levene 检验 | | 均值方程的 t 检验 | | | | | | |
|  |  | F | Sig. | t | df | Sig.(双侧) | 均值差值 | 标准误差值 | 差分的 95% 置信区间 | |
|  |  |  |  |  |  |  |  |  | 下限 | 上限 |
| VAR00007 | 假设方差相等 | .883 | .351 | 3.017 | 68 | .004 | 30.14286 | 9.98995 | 10.20822 | 50.07749 |
|  | 假设方差不相等 |  |  | 3.017 | 63.597 | .004 | 30.14286 | 9.98995 | 10.18320 | 50.10251 |

A2B1C2记者看到子弹击中的一个小偷逃离了现场。

A2B2C2记者看到社会奉献的一片爱心送到了学校。

| **组统计量** | | | | | |
| --- | --- | --- | --- | --- | --- |
|  | 首次注视抽象度分组 | N | 均值 | 标准差 | 均值的标准误 |
| VAR00008 | 1.00 | 35 | 236.5143 | 46.08392 | 7.78960 |
|  | 2.00 | 35 | 248.7714 | 66.42248 | 11.22745 |

| **独立样本检验** | | | | | | | | | | |
| --- | --- | --- | --- | --- | --- | --- | --- | --- | --- | --- |
|  | | 方差方程的 Levene 检验 | | 均值方程的 t 检验 | | | | | | |
|  |  | F | Sig. | t | df | Sig.(双侧) | 均值差值 | 标准误差值 | 差分的 95% 置信区间 | |
|  |  |  |  |  |  |  |  |  | 下限 | 上限 |
| VAR00008 | 假设方差相等 | 1.115 | .295 | -.897 | 68 | .373 | -12.25714 | 13.66505 | -39.52532 | 15.01104 |
|  | 假设方差不相等 |  |  | -.897 | 60.575 | .373 | -12.25714 | 13.66505 | -39.58597 | 15.07169 |

A1B1C2记者看到一个子弹击中的小偷逃离了现场。

A1B2C2记者看到一片社会奉献的爱心送到了学校。

| **组统计量** | | | | | |
| --- | --- | --- | --- | --- | --- |
|  | 首次注视抽象度分组 | N | 均值 | 标准差 | 均值的标准误 |
| VAR00009 | 1.00 | 35 | 225.3429 | 50.66728 | 8.56433 |
|  | 2.00 | 35 | 239.0857 | 41.06693 | 6.94158 |

| **独立样本检验** | | | | | | | | | | |
| --- | --- | --- | --- | --- | --- | --- | --- | --- | --- | --- |
|  | | 方差方程的 Levene 检验 | | 均值方程的 t 检验 | | | | | | |
|  |  | F | Sig. | t | df | Sig.(双侧) | 均值差值 | 标准误差值 | 差分的 95% 置信区间 | |
|  |  |  |  |  |  |  |  |  | 下限 | 上限 |
| VAR00009 | 假设方差相等 | 1.938 | .168 | -1.247 | 68 | .217 | -13.74286 | 11.02421 | -35.74133 | 8.25562 |
|  | 假设方差不相等 |  |  | -1.247 | 65.205 | .217 | -13.74286 | 11.02421 | -35.75842 | 8.27271 |

**凝视：**

**被试内：**

| **描述性统计量** | | | |
| --- | --- | --- | --- |
|  | 均值 | 标准 偏差 | N |
| A1B1C1 | 747.6571 | 62.36654 | 35 |
| A1B1C2 | 1212.6857 | 158.24121 | 35 |
| A1B2C1 | 1197.2571 | 129.56178 | 35 |
| A1B2C2 | 1358.4000 | 110.43928 | 35 |
| A2B1C1 | 1004.6000 | 98.86271 | 35 |
| A2B1C2 | 924.4571 | 111.34270 | 35 |
| A2B2C1 | 1098.3429 | 87.06149 | 35 |
| A2B2C2 | 816.8571 | 103.28434 | 35 |

| **Mauchly 的球形度检验^a^** | | | | | | | |
| --- | --- | --- | --- | --- | --- | --- | --- |
| 度量: MEASURE_1 | | | | | | | |
| 主体内效应 | Mauchly 的 W | 近似卡方 | df | Sig. | Epsilon^b^ | | |
|  |  |  |  |  | Greenhouse-Geisser | Huynh-Feldt | 下限 |
| A | 1.000 | .000 | 0 | . | 1.000 | 1.000 | 1.000 |
| B | 1.000 | .000 | 0 | . | 1.000 | 1.000 | 1.000 |
| C | 1.000 | .000 | 0 | . | 1.000 | 1.000 | 1.000 |
| A * B | 1.000 | .000 | 0 | . | 1.000 | 1.000 | 1.000 |
| A * C | 1.000 | .000 | 0 | . | 1.000 | 1.000 | 1.000 |
| B * C | 1.000 | .000 | 0 | . | 1.000 | 1.000 | 1.000 |
| A * B * C | 1.000 | .000 | 0 | . | 1.000 | 1.000 | 1.000 |
| 检验零假设，即标准正交转换因变量的误差协方差矩阵与一个单位矩阵成比例。 | | | | | | | |
| a. 设计 : 截距  主体内设计: A + B + C + A * B + A * C + B * C + A * B * C | | | | | | | |
| b. 可用于调整显著性平均检验的自由度。 在"主体内效应检验"表格中显示修正后的检验。 | | | | | | | |

| **主体内效应的检验** | | | | | | | | | |
| --- | --- | --- | --- | --- | --- | --- | --- | --- | --- |
| 度量: MEASURE_1 | | | | | | | | | |
| 源 | | III 型平方和 | df | 均方 | F | Sig. | 偏 Eta 方 | 非中心 参数 | 观测到的幂^a^ |
| A | 采用的球形度 | 1974168.289 | 1 | 1974168.289 | 122.281 | .000 | .782 | 122.281 | 1.000 |
|  | Greenhouse-Geisser | 1974168.289 | 1.000 | 1974168.289 | 122.281 | .000 | .782 | 122.281 | 1.000 |
|  | Huynh-Feldt | 1974168.289 | 1.000 | 1974168.289 | 122.281 | .000 | .782 | 122.281 | 1.000 |
|  | 下限 | 1974168.289 | 1.000 | 1974168.289 | 122.281 | .000 | .782 | 122.281 | 1.000 |
| 误差 (A) | 采用的球形度 | 548915.586 | 34 | 16144.576 |  |  |  |  |  |
|  | Greenhouse-Geisser | 548915.586 | 34.000 | 16144.576 |  |  |  |  |  |
|  | Huynh-Feldt | 548915.586 | 34.000 | 16144.576 |  |  |  |  |  |
|  | 下限 | 548915.586 | 34.000 | 16144.576 |  |  |  |  |  |
| B | 采用的球形度 | 1479154.289 | 1 | 1479154.289 | 115.315 | .000 | .772 | 115.315 | 1.000 |
|  | Greenhouse-Geisser | 1479154.289 | 1.000 | 1479154.289 | 115.315 | .000 | .772 | 115.315 | 1.000 |
|  | Huynh-Feldt | 1479154.289 | 1.000 | 1479154.289 | 115.315 | .000 | .772 | 115.315 | 1.000 |
|  | 下限 | 1479154.289 | 1.000 | 1479154.289 | 115.315 | .000 | .772 | 115.315 | 1.000 |
| 误差 (B) | 采用的球形度 | 436119.586 | 34 | 12827.047 |  |  |  |  |  |
|  | Greenhouse-Geisser | 436119.586 | 34.000 | 12827.047 |  |  |  |  |  |
|  | Huynh-Feldt | 436119.586 | 34.000 | 12827.047 |  |  |  |  |  |
|  | 下限 | 436119.586 | 34.000 | 12827.047 |  |  |  |  |  |
| C | 采用的球形度 | 306175.289 | 1 | 306175.289 | 26.234 | .000 | .436 | 26.234 | .999 |
|  | Greenhouse-Geisser | 306175.289 | 1.000 | 306175.289 | 26.234 | .000 | .436 | 26.234 | .999 |
|  | Huynh-Feldt | 306175.289 | 1.000 | 306175.289 | 26.234 | .000 | .436 | 26.234 | .999 |
|  | 下限 | 306175.289 | 1.000 | 306175.289 | 26.234 | .000 | .436 | 26.234 | .999 |
| 误差 (C) | 采用的球形度 | 396811.586 | 34 | 11670.929 |  |  |  |  |  |
|  | Greenhouse-Geisser | 396811.586 | 34.000 | 11670.929 |  |  |  |  |  |
|  | Huynh-Feldt | 396811.586 | 34.000 | 11670.929 |  |  |  |  |  |
|  | 下限 | 396811.586 | 34.000 | 11670.929 |  |  |  |  |  |
| A * B | 采用的球形度 | 1623518.004 | 1 | 1623518.004 | 118.599 | .000 | .777 | 118.599 | 1.000 |
|  | Greenhouse-Geisser | 1623518.004 | 1.000 | 1623518.004 | 118.599 | .000 | .777 | 118.599 | 1.000 |
|  | Huynh-Feldt | 1623518.004 | 1.000 | 1623518.004 | 118.599 | .000 | .777 | 118.599 | 1.000 |
|  | 下限 | 1623518.004 | 1.000 | 1623518.004 | 118.599 | .000 | .777 | 118.599 | 1.000 |
| 误差 (A*B) | 采用的球形度 | 465431.371 | 34 | 13689.158 |  |  |  |  |  |
|  | Greenhouse-Geisser | 465431.371 | 34.000 | 13689.158 |  |  |  |  |  |
|  | Huynh-Feldt | 465431.371 | 34.000 | 13689.158 |  |  |  |  |  |
|  | 下限 | 465431.371 | 34.000 | 13689.158 |  |  |  |  |  |
| A * C | 采用的球形度 | 4268901.175 | 1 | 4268901.175 | 427.624 | .000 | .926 | 427.624 | 1.000 |
|  | Greenhouse-Geisser | 4268901.175 | 1.000 | 4268901.175 | 427.624 | .000 | .926 | 427.624 | 1.000 |
|  | Huynh-Feldt | 4268901.175 | 1.000 | 4268901.175 | 427.624 | .000 | .926 | 427.624 | 1.000 |
|  | 下限 | 4268901.175 | 1.000 | 4268901.175 | 427.624 | .000 | .926 | 427.624 | 1.000 |
| 误差 (A*C) | 采用的球形度 | 339416.200 | 34 | 9982.829 |  |  |  |  |  |
|  | Greenhouse-Geisser | 339416.200 | 34.000 | 9982.829 |  |  |  |  |  |
|  | Huynh-Feldt | 339416.200 | 34.000 | 9982.829 |  |  |  |  |  |
|  | 下限 | 339416.200 | 34.000 | 9982.829 |  |  |  |  |  |
| B * C | 采用的球形度 | 1116744.604 | 1 | 1116744.604 | 122.511 | .000 | .783 | 122.511 | 1.000 |
|  | Greenhouse-Geisser | 1116744.604 | 1.000 | 1116744.604 | 122.511 | .000 | .783 | 122.511 | 1.000 |
|  | Huynh-Feldt | 1116744.604 | 1.000 | 1116744.604 | 122.511 | .000 | .783 | 122.511 | 1.000 |
|  | 下限 | 1116744.604 | 1.000 | 1116744.604 | 122.511 | .000 | .783 | 122.511 | 1.000 |
| 误差 (B*C) | 采用的球形度 | 309924.771 | 34 | 9115.434 |  |  |  |  |  |
|  | Greenhouse-Geisser | 309924.771 | 34.000 | 9115.434 |  |  |  |  |  |
|  | Huynh-Feldt | 309924.771 | 34.000 | 9115.434 |  |  |  |  |  |
|  | 下限 | 309924.771 | 34.000 | 9115.434 |  |  |  |  |  |
| A * B * C | 采用的球形度 | 46003.289 | 1 | 46003.289 | 4.625 | .039 | .120 | 4.625 | .552 |
|  | Greenhouse-Geisser | 46003.289 | 1.000 | 46003.289 | 4.625 | .039 | .120 | 4.625 | .552 |
|  | Huynh-Feldt | 46003.289 | 1.000 | 46003.289 | 4.625 | .039 | .120 | 4.625 | .552 |
|  | 下限 | 46003.289 | 1.000 | 46003.289 | 4.625 | .039 | .120 | 4.625 | .552 |
| 误差 (A*B*C) | 采用的球形度 | 338211.586 | 34 | 9947.400 |  |  |  |  |  |
|  | Greenhouse-Geisser | 338211.586 | 34.000 | 9947.400 |  |  |  |  |  |
|  | Huynh-Feldt | 338211.586 | 34.000 | 9947.400 |  |  |  |  |  |
|  | 下限 | 338211.586 | 34.000 | 9947.400 |  |  |  |  |  |
| a. 使用 alpha 的计算结果 = .05 | | | | | | | | | |

**项目内：**

| **主体内效应的检验** | | | | | | | | | |
| --- | --- | --- | --- | --- | --- | --- | --- | --- | --- |
| 度量: MEASURE_1 | | | | | | | | | |
| 源 | | III 型平方和 | df | 均方 | F | Sig. | 偏 Eta 方 | 非中心 参数 | 观测到的幂^a^ |
| A | 采用的球形度 | 1369338.785 | 1 | 1369338.785 | 137.072 | .000 | .816 | 137.072 | 1.000 |
|  | Greenhouse-Geisser | 1369338.785 | 1.000 | 1369338.785 | 137.072 | .000 | .816 | 137.072 | 1.000 |
|  | Huynh-Feldt | 1369338.785 | 1.000 | 1369338.785 | 137.072 | .000 | .816 | 137.072 | 1.000 |
|  | 下限 | 1369338.785 | 1.000 | 1369338.785 | 137.072 | .000 | .816 | 137.072 | 1.000 |
| 误差 (A) | 采用的球形度 | 309688.340 | 31 | 9989.946 |  |  |  |  |  |
|  | Greenhouse-Geisser | 309688.340 | 31.000 | 9989.946 |  |  |  |  |  |
|  | Huynh-Feldt | 309688.340 | 31.000 | 9989.946 |  |  |  |  |  |
|  | 下限 | 309688.340 | 31.000 | 9989.946 |  |  |  |  |  |
| B | 采用的球形度 | 879492.285 | 1 | 879492.285 | 82.443 | .000 | .727 | 82.443 | 1.000 |
|  | Greenhouse-Geisser | 879492.285 | 1.000 | 879492.285 | 82.443 | .000 | .727 | 82.443 | 1.000 |
|  | Huynh-Feldt | 879492.285 | 1.000 | 879492.285 | 82.443 | .000 | .727 | 82.443 | 1.000 |
|  | 下限 | 879492.285 | 1.000 | 879492.285 | 82.443 | .000 | .727 | 82.443 | 1.000 |
| 误差 (B) | 采用的球形度 | 330706.340 | 31 | 10667.946 |  |  |  |  |  |
|  | Greenhouse-Geisser | 330706.340 | 31.000 | 10667.946 |  |  |  |  |  |
|  | Huynh-Feldt | 330706.340 | 31.000 | 10667.946 |  |  |  |  |  |
|  | 下限 | 330706.340 | 31.000 | 10667.946 |  |  |  |  |  |
| C | 采用的球形度 | 150883.691 | 1 | 150883.691 | 14.876 | .001 | .324 | 14.876 | .962 |
|  | Greenhouse-Geisser | 150883.691 | 1.000 | 150883.691 | 14.876 | .001 | .324 | 14.876 | .962 |
|  | Huynh-Feldt | 150883.691 | 1.000 | 150883.691 | 14.876 | .001 | .324 | 14.876 | .962 |
|  | 下限 | 150883.691 | 1.000 | 150883.691 | 14.876 | .001 | .324 | 14.876 | .962 |
| 误差 (C) | 采用的球形度 | 314426.934 | 31 | 10142.804 |  |  |  |  |  |
|  | Greenhouse-Geisser | 314426.934 | 31.000 | 10142.804 |  |  |  |  |  |
|  | Huynh-Feldt | 314426.934 | 31.000 | 10142.804 |  |  |  |  |  |
|  | 下限 | 314426.934 | 31.000 | 10142.804 |  |  |  |  |  |
| A * B | 采用的球形度 | 1729389.379 | 1 | 1729389.379 | 203.919 | .000 | .868 | 203.919 | 1.000 |
|  | Greenhouse-Geisser | 1729389.379 | 1.000 | 1729389.379 | 203.919 | .000 | .868 | 203.919 | 1.000 |
|  | Huynh-Feldt | 1729389.379 | 1.000 | 1729389.379 | 203.919 | .000 | .868 | 203.919 | 1.000 |
|  | 下限 | 1729389.379 | 1.000 | 1729389.379 | 203.919 | .000 | .868 | 203.919 | 1.000 |
| 误差 (A*B) | 采用的球形度 | 262903.746 | 31 | 8480.766 |  |  |  |  |  |
|  | Greenhouse-Geisser | 262903.746 | 31.000 | 8480.766 |  |  |  |  |  |
|  | Huynh-Feldt | 262903.746 | 31.000 | 8480.766 |  |  |  |  |  |
|  | 下限 | 262903.746 | 31.000 | 8480.766 |  |  |  |  |  |
| A * C | 采用的球形度 | 3470536.129 | 1 | 3470536.129 | 401.152 | .000 | .928 | 401.152 | 1.000 |
|  | Greenhouse-Geisser | 3470536.129 | 1.000 | 3470536.129 | 401.152 | .000 | .928 | 401.152 | 1.000 |
|  | Huynh-Feldt | 3470536.129 | 1.000 | 3470536.129 | 401.152 | .000 | .928 | 401.152 | 1.000 |
|  | 下限 | 3470536.129 | 1.000 | 3470536.129 | 401.152 | .000 | .928 | 401.152 | 1.000 |
| 误差 (A*C) | 采用的球形度 | 268193.996 | 31 | 8651.419 |  |  |  |  |  |
|  | Greenhouse-Geisser | 268193.996 | 31.000 | 8651.419 |  |  |  |  |  |
|  | Huynh-Feldt | 268193.996 | 31.000 | 8651.419 |  |  |  |  |  |
|  | 下限 | 268193.996 | 31.000 | 8651.419 |  |  |  |  |  |
| B * C | 采用的球形度 | 736485.785 | 1 | 736485.785 | 72.832 | .000 | .701 | 72.832 | 1.000 |
|  | Greenhouse-Geisser | 736485.785 | 1.000 | 736485.785 | 72.832 | .000 | .701 | 72.832 | 1.000 |
|  | Huynh-Feldt | 736485.785 | 1.000 | 736485.785 | 72.832 | .000 | .701 | 72.832 | 1.000 |
|  | 下限 | 736485.785 | 1.000 | 736485.785 | 72.832 | .000 | .701 | 72.832 | 1.000 |
| 误差 (B*C) | 采用的球形度 | 313474.840 | 31 | 10112.092 |  |  |  |  |  |
|  | Greenhouse-Geisser | 313474.840 | 31.000 | 10112.092 |  |  |  |  |  |
|  | Huynh-Feldt | 313474.840 | 31.000 | 10112.092 |  |  |  |  |  |
|  | 下限 | 313474.840 | 31.000 | 10112.092 |  |  |  |  |  |
| A * B * C | 采用的球形度 | 81617.348 | 1 | 81617.348 | 7.863 | .009 | .202 | 7.863 | .775 |
|  | Greenhouse-Geisser | 81617.348 | 1.000 | 81617.348 | 7.863 | .009 | .202 | 7.863 | .775 |
|  | Huynh-Feldt | 81617.348 | 1.000 | 81617.348 | 7.863 | .009 | .202 | 7.863 | .775 |
|  | 下限 | 81617.348 | 1.000 | 81617.348 | 7.863 | .009 | .202 | 7.863 | .775 |
| 误差 (A*B*C) | 采用的球形度 | 321781.777 | 31 | 10380.057 |  |  |  |  |  |
|  | Greenhouse-Geisser | 321781.777 | 31.000 | 10380.057 |  |  |  |  |  |
|  | Huynh-Feldt | 321781.777 | 31.000 | 10380.057 |  |  |  |  |  |
|  | 下限 | 321781.777 | 31.000 | 10380.057 |  |  |  |  |  |
| a. 使用 alpha 的计算结果 = .05 | | | | | | | | | |

固定从句类型：(不区分抽象度)

记者看到一个穿着黑衣的小偷逃离了现场。90 4.78

记者看到一片献给孩子的爱心送到了学校。67 3.84

VS.

记者看到一个子弹击中的小偷逃离了现场。65 3.71

记者看到一片社会奉献的爱心送到了学校。60 3.62

| **组统计量** | | | | | |
| --- | --- | --- | --- | --- | --- |
|  | 凝视的句法间差异 | N | 均值 | 标准差 | 均值的标准误 |
| VAR00002 | 1.00 | 70 | 972.4571 | 247.90225 | 29.62999 |
|  | 2.00 | 70 | 1285.5429 | 154.05784 | 18.41343 |

| **独立样本检验** | | | | | | | | | | |
| --- | --- | --- | --- | --- | --- | --- | --- | --- | --- | --- |
|  | | 方差方程的 Levene 检验 | | 均值方程的 t 检验 | | | | | | |
|  |  | F | Sig. | t | df | Sig.(双侧) | 均值差值 | 标准误差值 | 差分的 95% 置信区间 | |
|  |  |  |  |  |  |  |  |  | 下限 | 上限 |
| VAR00002 | 假设方差相等 | 42.043 | .000 | -8.975 | 138 | .000 | -313.08571 | 34.88539 | -382.06473 | -244.10670 |
|  | 假设方差不相等 |  |  | -8.975 | 115.378 | .000 | -313.08571 | 34.88539 | -382.18456 | -243.98687 |

记者看到穿着黑衣的一个小偷逃离了现场。80 4.205

记者看到献给孩子的一片爱心送到了学校。89 4.52

VS.

记者看到子弹击中的一个小偷逃离了现场。75 4.15

记者看到社会奉献的一片爱心送到了学校。73 3.96

| **组统计量** | | | | | |
| --- | --- | --- | --- | --- | --- |
|  | 凝视的句法间差异 | N | 均值 | 标准差 | 均值的标准误 |
| VAR00003 | 1.00 | 70 | 1051.4714 | 103.82576 | 12.40955 |
|  | 2.00 | 70 | 870.6571 | 119.58962 | 14.29369 |

| **独立样本检验** | | | | | | | | | | |
| --- | --- | --- | --- | --- | --- | --- | --- | --- | --- | --- |
|  | | 方差方程的 Levene 检验 | | 均值方程的 t 检验 | | | | | | |
|  |  | F | Sig. | t | df | Sig.(双侧) | 均值差值 | 标准误差值 | 差分的 95% 置信区间 | |
|  |  |  |  |  |  |  |  |  | 下限 | 上限 |
| VAR00003 | 假设方差相等 | .282 | .596 | 9.552 | 138 | .000 | 180.81429 | 18.92899 | 143.38593 | 218.24265 |
|  | 假设方差不相等 |  |  | 9.552 | 135.332 | .000 | 180.81429 | 18.92899 | 143.37940 | 218.24917 |

结论：早期句法激活，且量词位置置内、外不会影响句子接受度，句法的自主

第二步 加入语义因素

**固定从句类型：**

**抽象度：**

1 A1B1C1记者看到一个穿着黑衣的小偷逃离了现场。90 4.78

A1B2C1记者看到一片献给孩子的爱心送到了学校。67 3.84

| **组统计量** | | | | | |
| --- | --- | --- | --- | --- | --- |
|  | 凝视抽象度分组 | N | 均值 | 标准差 | 均值的标准误 |
| VAR00012 | 1.00 | 35 | 747.6571 | 62.36654 | 10.54187 |
|  | 2.00 | 35 | 1197.2571 | 129.56178 | 21.89994 |

| **独立样本检验** | | | | | | | | | | |
| --- | --- | --- | --- | --- | --- | --- | --- | --- | --- | --- |
|  | | 方差方程的 Levene 检验 | | 均值方程的 t 检验 | | | | | | |
|  |  | F | Sig. | t | df | Sig.(双侧) | 均值差值 | 标准误差值 | 差分的 95% 置信区间 | |
|  |  |  |  |  |  |  |  |  | 下限 | 上限 |
| VAR00012 | 假设方差相等 | 18.588 | .000 | -18.498 | 68 | .000 | -449.60000 | 24.30511 | -498.10009 | -401.09991 |
|  | 假设方差不相等 |  |  | -18.498 | 48.954 | .000 | -449.60000 | 24.30511 | -498.44412 | -400.75588 |

2 A2B1C1记者看到穿着黑衣的一个小偷逃离了现场。80 4.31

A2B2C1记者看到献给孩子的一片爱心送到了学校。89 4.52

| **组统计量** | | | | | |
| --- | --- | --- | --- | --- | --- |
|  | 凝视抽象度分组 | N | 均值 | 标准差 | 均值的标准误 |
| VAR00013 | 1.00 | 35 | 1004.6000 | 98.86271 | 16.71085 |
|  | 2.00 | 35 | 1098.3429 | 87.06149 | 14.71608 |

| **独立样本检验** | | | | | | | | | | |
| --- | --- | --- | --- | --- | --- | --- | --- | --- | --- | --- |
|  | | 方差方程的 Levene 检验 | | 均值方程的 t 检验 | | | | | | |
|  |  | F | Sig. | t | df | Sig.(双侧) | 均值差值 | 标准误差值 | 差分的 95% 置信区间 | |
|  |  |  |  |  |  |  |  |  | 下限 | 上限 |
| VAR00013 | 假设方差相等 | .529 | .470 | -4.210 | 68 | .000 | -93.74286 | 22.26691 | -138.17579 | -49.30993 |
|  | 假设方差不相等 |  |  | -4.210 | 66.930 | .000 | -93.74286 | 22.26691 | -138.18865 | -49.29706 |

3 A2B1C2记者看到子弹击中的一个小偷逃离了现场。75 4.15

A2B2C2记者看到社会奉献的一片爱心送到了学校。73 3.96

| **组统计量** | | | | | |
| --- | --- | --- | --- | --- | --- |
|  | 凝视抽象度分组 | N | 均值 | 标准差 | 均值的标准误 |
| VAR00014 | 1.00 | 35 | 924.4571 | 111.34270 | 18.82035 |
|  | 2.00 | 35 | 816.8571 | 103.28434 | 17.45824 |

| **独立样本检验** | | | | | | | | | | |
| --- | --- | --- | --- | --- | --- | --- | --- | --- | --- | --- |
|  | | 方差方程的 Levene 检验 | | 均值方程的 t 检验 | | | | | | |
|  |  | F | Sig. | t | df | Sig.(双侧) | 均值差值 | 标准误差值 | 差分的 95% 置信区间 | |
|  |  |  |  |  |  |  |  |  | 下限 | 上限 |
| VAR00014 | 假设方差相等 | .354 | .554 | 4.192 | 68 | .000 | 107.60000 | 25.67091 | 56.37449 | 158.82551 |
|  | 假设方差不相等 |  |  | 4.192 | 67.620 | .000 | 107.60000 | 25.67091 | 56.36927 | 158.83073 |

4 A1B1C2记者看到一个子弹击中的小偷逃离了现场。65 3.71（接受度无差异）

A1B2C2记者看到一片社会奉献的爱心送到了学校。60 3.62

| **组统计量** | | | | | |
| --- | --- | --- | --- | --- | --- |
|  | 凝视抽象度分组 | N | 均值 | 标准差 | 均值的标准误 |
| VAR00015 | 1.00 | 35 | 1212.6857 | 158.24121 | 26.74765 |
|  | 2.00 | 35 | 1358.4000 | 110.43928 | 18.66765 |

| **独立样本检验** | | | | | | | | | | |
| --- | --- | --- | --- | --- | --- | --- | --- | --- | --- | --- |
|  | | 方差方程的 Levene 检验 | | 均值方程的 t 检验 | | | | | | |
|  |  | F | Sig. | t | df | Sig.(双侧) | 均值差值 | 标准误差值 | 差分的 95% 置信区间 | |
|  |  |  |  |  |  |  |  |  | 下限 | 上限 |
| VAR00015 | 假设方差相等 | 4.529 | .037 | -4.467 | 68 | .000 | -145.71429 | 32.61775 | -210.80199 | -80.62658 |
|  | 假设方差不相等 |  |  | -4.467 | 60.771 | .000 | -145.71429 | 32.61775 | -210.94249 | -80.48608 |

**回视：**

**被试内主效应：**

| **描述性统计量** | | | |
| --- | --- | --- | --- |
|  | 均值 | 标准 偏差 | N |
| A1B1C1 | 1395.1143 | 127.75638 | 35 |
| A1B1C2 | 1563.9143 | 178.35364 | 35 |
| A1B2C1 | 1475.7714 | 145.57252 | 35 |
| A1B2C2 | 1602.1714 | 156.01084 | 35 |
| A2B1C1 | 1473.1429 | 97.23687 | 35 |
| A2B1C2 | 1496.9143 | 133.20779 | 35 |
| A2B2C1 | 1400.5429 | 113.59845 | 35 |
| A2B2C2 | 1515.1429 | 133.15982 | 35 |

Table. main effects and interactive effects of sources in Experiment 3眼动指标

| **Source(Type)** | **首次** | | | **凝视** |  | |  |  |  |  |
| --- | --- | --- | --- | --- | --- | --- | --- | --- | --- | --- |
|  | **F** | ***p*** | **η^2^_p_** | **F** | ***p*** | **η^2^_p_** | **F** |  | ***p*** | **η^2^_p_** |
| **A(IMN/OMN)** | F_1_= 3.154 | .085 | .085 | F_1_=122.281 | .000 | .782 | F_1_= | 8.807 | .005 | .206 |
|  | F_2_= 3.865 | .058 | .111 | F_2_=137.072 | .000 | .816 | F_2_= | .980 | .330 | .031 |
| **B(CN/AN)** | F_1_= .880 | .355 | .025 | F_1_=115.315 | .000 | .772 | F_1_= | 1.257 | .270 | .036 |
|  | F_2_= .174 | .679 | .006 | F_2_=82.443 | .000 | .727 | F_2_= | 8.153 | .008 | .208 |
| **C(SRC/ORC)** | F_1_= 10.644 | .003 | .238 | F_1_=26.234 | .000 | .436 | F_1_= | 47.320 | .000 | .582 |
|  | F_2_= 6.836 | .014 | .181 | F_2_=14.876 | .001 | .324 | F_2_= | 83.412 | .000 | .729 |
| **A * B** | F_1_= 5.966 | .020 | .149 | F_1_=118.599 | .000 | .777 | F_1_= | 7.204 | .011 | .175 |
|  | F_2_= 3.057 | .090 | .090 | F_2_=203.919 | .000 | .868 | F_2_= | 7.571 | .010 | .196 |
| **A * C** | F_1_= .023 | .880 | .001 | F_1_=427.624 | .000 | .926 | F_1_= | 5.094 | .031 | .130 |
|  | F_2_= .088 | .769 | .003 | F_2_=401.152 | .000 | .928 | F_2_= | 6.709 | .014 | .178 |
| **B * C** | F_1_= 1.467 | .234 | .041 | F_1_=122.511 | .000 | .783 | F_1_= | .547 | .465 | .016 |
|  | F_2_= .605 | .443 | .019 | F_2_=72.832 | .000 | .701 | F_2_= | .237 | .630 | .008 |
| **A * B * C** | F_1_= 5.530 | .025 | .140 | F_1_=4.625 | .039 | .120 | F_1_= | 3.761 | .061 | .100 |
|  | F_2_= 4.867 | .035 | .136 | F_2_=7.863 | .009 | .202 | F_2_= | 1.056 | .312 | .033 |

F(1), df is 1,34; F(2), df is 1, 31 as is illustrated in table XXX 接受度反应时表格.

| **主体内效应的检验** | | | | | | | | | |
| --- | --- | --- | --- | --- | --- | --- | --- | --- | --- |
| 度量: MEASURE_1 | | | | | | | | | |
| 源 | | III 型平方和 | df | 均方 | F | Sig. | 偏 Eta 方 | 非中心 参数 | 观测到的幂^a^ |
| A | 采用的球形度 | 100056.604 | 1 | 100056.604 | 8.807 | .005 | .206 | 8.807 | .822 |
|  | Greenhouse-Geisser | 100056.604 | 1.000 | 100056.604 | 8.807 | .005 | .206 | 8.807 | .822 |
|  | Huynh-Feldt | 100056.604 | 1.000 | 100056.604 | 8.807 | .005 | .206 | 8.807 | .822 |
|  | 下限 | 100056.604 | 1.000 | 100056.604 | 8.807 | .005 | .206 | 8.807 | .822 |
| 误差 (A) | 采用的球形度 | 386274.771 | 34 | 11361.023 |  |  |  |  |  |
|  | Greenhouse-Geisser | 386274.771 | 34.000 | 11361.023 |  |  |  |  |  |
|  | Huynh-Feldt | 386274.771 | 34.000 | 11361.023 |  |  |  |  |  |
|  | 下限 | 386274.771 | 34.000 | 11361.023 |  |  |  |  |  |
| B | 采用的球形度 | 18225.289 | 1 | 18225.289 | 1.257 | .270 | .036 | 1.257 | .193 |
|  | Greenhouse-Geisser | 18225.289 | 1.000 | 18225.289 | 1.257 | .270 | .036 | 1.257 | .193 |
|  | Huynh-Feldt | 18225.289 | 1.000 | 18225.289 | 1.257 | .270 | .036 | 1.257 | .193 |
|  | 下限 | 18225.289 | 1.000 | 18225.289 | 1.257 | .270 | .036 | 1.257 | .193 |
| 误差 (B) | 采用的球形度 | 492871.586 | 34 | 14496.223 |  |  |  |  |  |
|  | Greenhouse-Geisser | 492871.586 | 34.000 | 14496.223 |  |  |  |  |  |
|  | Huynh-Feldt | 492871.586 | 34.000 | 14496.223 |  |  |  |  |  |
|  | 下限 | 492871.586 | 34.000 | 14496.223 |  |  |  |  |  |
| C | 采用的球形度 | 822430.804 | 1 | 822430.804 | 47.320 | .000 | .582 | 47.320 | 1.000 |
|  | Greenhouse-Geisser | 822430.804 | 1.000 | 822430.804 | 47.320 | .000 | .582 | 47.320 | 1.000 |
|  | Huynh-Feldt | 822430.804 | 1.000 | 822430.804 | 47.320 | .000 | .582 | 47.320 | 1.000 |
|  | 下限 | 822430.804 | 1.000 | 822430.804 | 47.320 | .000 | .582 | 47.320 | 1.000 |
| 误差 (C) | 采用的球形度 | 590930.571 | 34 | 17380.311 |  |  |  |  |  |
|  | Greenhouse-Geisser | 590930.571 | 34.000 | 17380.311 |  |  |  |  |  |
|  | Huynh-Feldt | 590930.571 | 34.000 | 17380.311 |  |  |  |  |  |
|  | 下限 | 590930.571 | 34.000 | 17380.311 |  |  |  |  |  |
| A * B | 采用的球形度 | 131372.232 | 1 | 131372.232 | 7.204 | .011 | .175 | 7.204 | .741 |
|  | Greenhouse-Geisser | 131372.232 | 1.000 | 131372.232 | 7.204 | .011 | .175 | 7.204 | .741 |
|  | Huynh-Feldt | 131372.232 | 1.000 | 131372.232 | 7.204 | .011 | .175 | 7.204 | .741 |
|  | 下限 | 131372.232 | 1.000 | 131372.232 | 7.204 | .011 | .175 | 7.204 | .741 |
| 误差 (A*B) | 采用的球形度 | 619985.643 | 34 | 18234.872 |  |  |  |  |  |
|  | Greenhouse-Geisser | 619985.643 | 34.000 | 18234.872 |  |  |  |  |  |
|  | Huynh-Feldt | 619985.643 | 34.000 | 18234.872 |  |  |  |  |  |
|  | 下限 | 619985.643 | 34.000 | 18234.872 |  |  |  |  |  |
| A * C | 采用的球形度 | 107604.004 | 1 | 107604.004 | 5.094 | .031 | .130 | 5.094 | .592 |
|  | Greenhouse-Geisser | 107604.004 | 1.000 | 107604.004 | 5.094 | .031 | .130 | 5.094 | .592 |
|  | Huynh-Feldt | 107604.004 | 1.000 | 107604.004 | 5.094 | .031 | .130 | 5.094 | .592 |
|  | 下限 | 107604.004 | 1.000 | 107604.004 | 5.094 | .031 | .130 | 5.094 | .592 |
| 误差 (A*C) | 采用的球形度 | 718172.371 | 34 | 21122.717 |  |  |  |  |  |
|  | Greenhouse-Geisser | 718172.371 | 34.000 | 21122.717 |  |  |  |  |  |
|  | Huynh-Feldt | 718172.371 | 34.000 | 21122.717 |  |  |  |  |  |
|  | 下限 | 718172.371 | 34.000 | 21122.717 |  |  |  |  |  |
| B * C | 采用的球形度 | 10260.804 | 1 | 10260.804 | .547 | .465 | .016 | .547 | .111 |
|  | Greenhouse-Geisser | 10260.804 | 1.000 | 10260.804 | .547 | .465 | .016 | .547 | .111 |
|  | Huynh-Feldt | 10260.804 | 1.000 | 10260.804 | .547 | .465 | .016 | .547 | .111 |
|  | 下限 | 10260.804 | 1.000 | 10260.804 | .547 | .465 | .016 | .547 | .111 |
| 误差 (B*C) | 采用的球形度 | 638208.071 | 34 | 18770.826 |  |  |  |  |  |
|  | Greenhouse-Geisser | 638208.071 | 34.000 | 18770.826 |  |  |  |  |  |
|  | Huynh-Feldt | 638208.071 | 34.000 | 18770.826 |  |  |  |  |  |
|  | 下限 | 638208.071 | 34.000 | 18770.826 |  |  |  |  |  |
| A * B * C | 采用的球形度 | 77655.604 | 1 | 77655.604 | 3.761 | .061 | .100 | 3.761 | .470 |
|  | Greenhouse-Geisser | 77655.604 | 1.000 | 77655.604 | 3.761 | .061 | .100 | 3.761 | .470 |
|  | Huynh-Feldt | 77655.604 | 1.000 | 77655.604 | 3.761 | .061 | .100 | 3.761 | .470 |
|  | 下限 | 77655.604 | 1.000 | 77655.604 | 3.761 | .061 | .100 | 3.761 | .470 |
| 误差 (A*B*C) | 采用的球形度 | 701992.271 | 34 | 20646.832 |  |  |  |  |  |
|  | Greenhouse-Geisser | 701992.271 | 34.000 | 20646.832 |  |  |  |  |  |
|  | Huynh-Feldt | 701992.271 | 34.000 | 20646.832 |  |  |  |  |  |
|  | 下限 | 701992.271 | 34.000 | 20646.832 |  |  |  |  |  |
| a. 使用 alpha 的计算结果 = .05 | | | | | | | | | |

**项目内主效应：**

| **主体内效应的检验** | | | | | | | | | |
| --- | --- | --- | --- | --- | --- | --- | --- | --- | --- |
| 度量: MEASURE_1 | | | | | | | | | |
| 源 | | III 型平方和 | df | 均方 | F | Sig. | 偏 Eta 方 | 非中心 参数 | 观测到的幂^a^ |
| A | 采用的球形度 | 17539.691 | 1 | 17539.691 | .980 | .330 | .031 | .980 | .160 |
|  | Greenhouse-Geisser | 17539.691 | 1.000 | 17539.691 | .980 | .330 | .031 | .980 | .160 |
|  | Huynh-Feldt | 17539.691 | 1.000 | 17539.691 | .980 | .330 | .031 | .980 | .160 |
|  | 下限 | 17539.691 | 1.000 | 17539.691 | .980 | .330 | .031 | .980 | .160 |
| 误差 (A) | 采用的球形度 | 554806.934 | 31 | 17896.998 |  |  |  |  |  |
|  | Greenhouse-Geisser | 554806.934 | 31.000 | 17896.998 |  |  |  |  |  |
|  | Huynh-Feldt | 554806.934 | 31.000 | 17896.998 |  |  |  |  |  |
|  | 下限 | 554806.934 | 31.000 | 17896.998 |  |  |  |  |  |
| B | 采用的球形度 | 55607.535 | 1 | 55607.535 | 8.153 | .008 | .208 | 8.153 | .790 |
|  | Greenhouse-Geisser | 55607.535 | 1.000 | 55607.535 | 8.153 | .008 | .208 | 8.153 | .790 |
|  | Huynh-Feldt | 55607.535 | 1.000 | 55607.535 | 8.153 | .008 | .208 | 8.153 | .790 |
|  | 下限 | 55607.535 | 1.000 | 55607.535 | 8.153 | .008 | .208 | 8.153 | .790 |
| 误差 (B) | 采用的球形度 | 211446.090 | 31 | 6820.842 |  |  |  |  |  |
|  | Greenhouse-Geisser | 211446.090 | 31.000 | 6820.842 |  |  |  |  |  |
|  | Huynh-Feldt | 211446.090 | 31.000 | 6820.842 |  |  |  |  |  |
|  | 下限 | 211446.090 | 31.000 | 6820.842 |  |  |  |  |  |
| C | 采用的球形度 | 749198.441 | 1 | 749198.441 | 83.412 | .000 | .729 | 83.412 | 1.000 |
|  | Greenhouse-Geisser | 749198.441 | 1.000 | 749198.441 | 83.412 | .000 | .729 | 83.412 | 1.000 |
|  | Huynh-Feldt | 749198.441 | 1.000 | 749198.441 | 83.412 | .000 | .729 | 83.412 | 1.000 |
|  | 下限 | 749198.441 | 1.000 | 749198.441 | 83.412 | .000 | .729 | 83.412 | 1.000 |
| 误差 (C) | 采用的球形度 | 278439.184 | 31 | 8981.909 |  |  |  |  |  |
|  | Greenhouse-Geisser | 278439.184 | 31.000 | 8981.909 |  |  |  |  |  |
|  | Huynh-Feldt | 278439.184 | 31.000 | 8981.909 |  |  |  |  |  |
|  | 下限 | 278439.184 | 31.000 | 8981.909 |  |  |  |  |  |
| A * B | 采用的球形度 | 102920.660 | 1 | 102920.660 | 7.571 | .010 | .196 | 7.571 | .760 |
|  | Greenhouse-Geisser | 102920.660 | 1.000 | 102920.660 | 7.571 | .010 | .196 | 7.571 | .760 |
|  | Huynh-Feldt | 102920.660 | 1.000 | 102920.660 | 7.571 | .010 | .196 | 7.571 | .760 |
|  | 下限 | 102920.660 | 1.000 | 102920.660 | 7.571 | .010 | .196 | 7.571 | .760 |
| 误差 (A*B) | 采用的球形度 | 421402.965 | 31 | 13593.644 |  |  |  |  |  |
|  | Greenhouse-Geisser | 421402.965 | 31.000 | 13593.644 |  |  |  |  |  |
|  | Huynh-Feldt | 421402.965 | 31.000 | 13593.644 |  |  |  |  |  |
|  | 下限 | 421402.965 | 31.000 | 13593.644 |  |  |  |  |  |
| A * C | 采用的球形度 | 81260.629 | 1 | 81260.629 | 6.709 | .014 | .178 | 6.709 | .709 |
|  | Greenhouse-Geisser | 81260.629 | 1.000 | 81260.629 | 6.709 | .014 | .178 | 6.709 | .709 |
|  | Huynh-Feldt | 81260.629 | 1.000 | 81260.629 | 6.709 | .014 | .178 | 6.709 | .709 |
|  | 下限 | 81260.629 | 1.000 | 81260.629 | 6.709 | .014 | .178 | 6.709 | .709 |
| 误差 (A*C) | 采用的球形度 | 375471.996 | 31 | 12112.000 |  |  |  |  |  |
|  | Greenhouse-Geisser | 375471.996 | 31.000 | 12112.000 |  |  |  |  |  |
|  | Huynh-Feldt | 375471.996 | 31.000 | 12112.000 |  |  |  |  |  |
|  | 下限 | 375471.996 | 31.000 | 12112.000 |  |  |  |  |  |
| B * C | 采用的球形度 | 3045.660 | 1 | 3045.660 | .237 | .630 | .008 | .237 | .076 |
|  | Greenhouse-Geisser | 3045.660 | 1.000 | 3045.660 | .237 | .630 | .008 | .237 | .076 |
|  | Huynh-Feldt | 3045.660 | 1.000 | 3045.660 | .237 | .630 | .008 | .237 | .076 |
|  | 下限 | 3045.660 | 1.000 | 3045.660 | .237 | .630 | .008 | .237 | .076 |
| 误差 (B*C) | 采用的球形度 | 398011.965 | 31 | 12839.096 |  |  |  |  |  |
|  | Greenhouse-Geisser | 398011.965 | 31.000 | 12839.096 |  |  |  |  |  |
|  | Huynh-Feldt | 398011.965 | 31.000 | 12839.096 |  |  |  |  |  |
|  | 下限 | 398011.965 | 31.000 | 12839.096 |  |  |  |  |  |
| A * B * C | 采用的球形度 | 13820.941 | 1 | 13820.941 | 1.056 | .312 | .033 | 1.056 | .169 |
|  | Greenhouse-Geisser | 13820.941 | 1.000 | 13820.941 | 1.056 | .312 | .033 | 1.056 | .169 |
|  | Huynh-Feldt | 13820.941 | 1.000 | 13820.941 | 1.056 | .312 | .033 | 1.056 | .169 |
|  | 下限 | 13820.941 | 1.000 | 13820.941 | 1.056 | .312 | .033 | 1.056 | .169 |
| 误差 (A*B*C) | 采用的球形度 | 405601.684 | 31 | 13083.925 |  |  |  |  |  |
|  | Greenhouse-Geisser | 405601.684 | 31.000 | 13083.925 |  |  |  |  |  |
|  | Huynh-Feldt | 405601.684 | 31.000 | 13083.925 |  |  |  |  |  |
|  | 下限 | 405601.684 | 31.000 | 13083.925 |  |  |  |  |  |
| a. 使用 alpha 的计算结果 = .05 | | | | | | | | | |

固定从句类型：(不区分抽象度)

记者看到一个穿着黑衣的小偷逃离了现场。90 4.78

记者看到一片献给孩子的爱心送到了学校。67 3.84

VS.

记者看到一个子弹击中的小偷逃离了现场。65 3.71

记者看到一片社会奉献的爱心送到了学校。60 3.62

| **组统计量** | | | | | |
| --- | --- | --- | --- | --- | --- |
|  | 回视的句法间差异 | N | 均值 | 标准差 | 均值的标准误 |
| VAR00009 | 1.00 | 70 | 1435.4429 | 141.89664 | 16.95989 |
|  | 2.00 | 70 | 1583.0429 | 167.44858 | 20.01393 |

| **独立样本检验** | | | | | | | | | | |
| --- | --- | --- | --- | --- | --- | --- | --- | --- | --- | --- |
|  | | 方差方程的 Levene 检验 | | 均值方程的 t 检验 | | | | | | |
|  |  | F | Sig. | t | df | Sig.(双侧) | 均值差值 | 标准误差值 | 差分的 95% 置信区间 | |
|  |  |  |  |  |  |  |  |  | 下限 | 上限 |
| VAR00009 | 假设方差相等 | 1.244 | .267 | -5.626 | 138 | .000 | -147.60000 | 26.23348 | -199.47155 | -95.72845 |
|  | 假设方差不相等 |  |  | -5.626 | 134.382 | .000 | -147.60000 | 26.23348 | -199.48391 | -95.71609 |

记者看到穿着黑衣的一个小偷逃离了现场。80 4.205

记者看到献给孩子的一片爱心送到了学校。89 4.52

VS.

记者看到子弹击中的一个小偷逃离了现场。75 4.15

记者看到社会奉献的一片爱心送到了学校。73 3.96

| **组统计量** | | | | | |
| --- | --- | --- | --- | --- | --- |
|  | 回视的句法间差异 | N | 均值 | 标准差 | 均值的标准误 |
| VAR00010 | 1.00 | 70 | 1436.8429 | 111.15111 | 13.28510 |
|  | 2.00 | 70 | 1506.0286 | 132.53350 | 15.84078 |

| **独立样本检验** | | | | | | | | | | |
| --- | --- | --- | --- | --- | --- | --- | --- | --- | --- | --- |
|  | | 方差方程的 Levene 检验 | | 均值方程的 t 检验 | | | | | | |
|  |  | F | Sig. | t | df | Sig.(双侧) | 均值差值 | 标准误差值 | 差分的 95% 置信区间 | |
|  |  |  |  |  |  |  |  |  | 下限 | 上限 |
| VAR00010 | 假设方差相等 | 2.126 | .147 | -3.346 | 138 | .001 | -69.18571 | 20.67424 | -110.06497 | -28.30646 |
|  | 假设方差不相等 |  |  | -3.346 | 133.938 | .001 | -69.18571 | 20.67424 | -110.07593 | -28.29549 |

第二步：

**固定从句类型：**

**抽象度：**

1 A1B1C1记者看到一个穿着黑衣的小偷逃离了现场。90 4.78

A1B2C1记者看到一片献给孩子的爱心送到了学校。67 3.84

| **组统计量** | | | | | |
| --- | --- | --- | --- | --- | --- |
|  | 回视抽象度分组 | N | 均值 | 标准差 | 均值的标准误 |
| VAR00027 | 1.00 | 35 | 1395.1143 | 127.75638 | 21.59477 |
|  | 2.00 | 35 | 1475.7714 | 145.57252 | 24.60625 |

| **独立样本检验** | | | | | | | | | | |
| --- | --- | --- | --- | --- | --- | --- | --- | --- | --- | --- |
|  | | 方差方程的 Levene 检验 | | 均值方程的 t 检验 | | | | | | |
|  |  | F | Sig. | t | df | Sig.(双侧) | 均值差值 | 标准误差值 | 差分的 95% 置信区间 | |
|  |  |  |  |  |  |  |  |  | 下限 | 上限 |
| VAR00027 | 假设方差相等 | .269 | .606 | -2.464 | 68 | .016 | -80.65714 | 32.73838 | -145.98556 | -15.32873 |
|  | 假设方差不相等 |  |  | -2.464 | 66.873 | .016 | -80.65714 | 32.73838 | -146.00550 | -15.30879 |

2 A2B1C1记者看到穿着黑衣的一个小偷逃离了现场。80 4.31

A2B2C1记者看到献给孩子的一片爱心送到了学校。89 4.52

| **组统计量** | | | | | |
| --- | --- | --- | --- | --- | --- |
|  | 回视抽象度分组 | N | 均值 | 标准差 | 均值的标准误 |
| VAR00028 | 1.00 | 35 | 1473.1429 | 97.23687 | 16.43603 |
|  | 2.00 | 35 | 1400.5429 | 113.59845 | 19.20164 |

| **独立样本检验** | | | | | | | | | | |
| --- | --- | --- | --- | --- | --- | --- | --- | --- | --- | --- |
|  | | 方差方程的 Levene 检验 | | 均值方程的 t 检验 | | | | | | |
|  |  | F | Sig. | t | df | Sig.(双侧) | 均值差值 | 标准误差值 | 差分的 95% 置信区间 | |
|  |  |  |  |  |  |  |  |  | 下限 | 上限 |
| VAR00028 | 假设方差相等 | 1.824 | .181 | 2.872 | 68 | .005 | 72.60000 | 25.27541 | 22.16371 | 123.03629 |
|  | 假设方差不相等 |  |  | 2.872 | 66.419 | .005 | 72.60000 | 25.27541 | 22.14196 | 123.05804 |

3 A2B1C2记者看到子弹击中的一个小偷逃离了现场。75 4.15

A2B2C2记者看到社会奉献的一片爱心送到了学校。73 3.96

| **组统计量** | | | | | |
| --- | --- | --- | --- | --- | --- |
|  | 回视抽象度分组 | N | 均值 | 标准差 | 均值的标准误 |
| VAR00029 | 1.00 | 35 | 1496.9143 | 133.20779 | 22.51623 |
|  | 2.00 | 35 | 1515.1429 | 133.15982 | 22.50812 |

| **独立样本检验** | | | | | | | | | | |
| --- | --- | --- | --- | --- | --- | --- | --- | --- | --- | --- |
|  | | 方差方程的 Levene 检验 | | 均值方程的 t 检验 | | | | | | |
|  |  | F | Sig. | t | df | Sig.(双侧) | 均值差值 | 标准误差值 | 差分的 95% 置信区间 | |
|  |  |  |  |  |  |  |  |  | 下限 | 上限 |
| VAR00029 | 假设方差相等 | .233 | .631 | -.573 | 68 | .569 | -18.22857 | 31.83702 | -81.75836 | 45.30121 |
|  | 假设方差不相等 |  |  | -.573 | 68.000 | .569 | -18.22857 | 31.83702 | -81.75836 | 45.30121 |

4 A1B1C2记者看到一个子弹击中的小偷逃离了现场。65 3.71（接受度无差异）

A1B2C2记者看到一片社会奉献的爱心送到了学校。60 3.62

| **组统计量** | | | | | |
| --- | --- | --- | --- | --- | --- |
|  | 回视抽象度分组 | N | 均值 | 标准差 | 均值的标准误 |
| VAR00030 | 1.00 | 35 | 1563.9143 | 178.35364 | 30.14727 |
|  | 2.00 | 35 | 1602.1714 | 156.01084 | 26.37064 |

| **独立样本检验** | | | | | | | | | | |
| --- | --- | --- | --- | --- | --- | --- | --- | --- | --- | --- |
|  | | 方差方程的 Levene 检验 | | 均值方程的 t 检验 | | | | | | |
|  |  | F | Sig. | t | df | Sig.(双侧) | 均值差值 | 标准误差值 | 差分的 95% 置信区间 | |
|  |  |  |  |  |  |  |  |  | 下限 | 上限 |
| VAR00030 | 假设方差相等 | .117 | .733 | -.955 | 68 | .343 | -38.25714 | 40.05332 | -118.18230 | 41.66802 |
|  | 假设方差不相等 |  |  | -.955 | 66.817 | .343 | -38.25714 | 40.05332 | -118.20793 | 41.69364 |
